# Endometrial whole-slide images dataset for detection of malignancy in endometrial biopsies

**DOI:** 10.1093/gigascience/giaf147

**Published:** 2025-12-05

**Authors:** Mahnaz Mohammadi, Christina Fell, Sarah Bell, Gareth Bryson, Sheeba Syed, Prakash Konanahalli, David Harris-Birtill, Ognjen Arandjelovic, Clare Orange, Prishma Shahi, In Hwa Um, James D Blackwood, David J Harrison

**Affiliations:** School of Medicine, University of St Andrews, North Haugh, St Andrews, KY16 9TF, United Kingdom; School of Medicine, University of St Andrews, North Haugh, St Andrews, KY16 9TF, United Kingdom; Department of Pathology, Queen Elizabeth University Hospital, Glasglow, G51 4TF, United Kingdom; Department of Pathology, Queen Elizabeth University Hospital, Glasglow, G51 4TF, United Kingdom; Department of Pathology, Queen Elizabeth University Hospital, Glasglow, G51 4TF, United Kingdom; Department of Pathology, Queen Elizabeth University Hospital, Glasglow, G51 4TF, United Kingdom; School of Computer Science, University of St Andrews, North Haugh, St Andrews, KY16 9SX, United Kingdom; School of Computer Science, University of St Andrews, North Haugh, St Andrews, KY16 9SX, United Kingdom; School of Medicine, University of St Andrews, North Haugh, St Andrews, KY16 9TF, United Kingdom; Department of Pathology, Queen Elizabeth University Hospital, Glasglow, G51 4TF, United Kingdom; School of Medicine, University of St Andrews, North Haugh, St Andrews, KY16 9TF, United Kingdom; School of Medicine, University of St Andrews, North Haugh, St Andrews, KY16 9TF, United Kingdom; Pathology, Division of Laboratory Medicine, Royal Infirmary of Edinburgh, Edinburgh, EH16 4SA, United Kingdom; School of Medicine, University of St Andrews, North Haugh, St Andrews, KY16 9TF, United Kingdom; School of Medicine, University of St Andrews, North Haugh, St Andrews, KY16 9TF, United Kingdom; Pathology, Division of Laboratory Medicine, Royal Infirmary of Edinburgh, Edinburgh, EH16 4SA, United Kingdom

**Keywords:** endometrium, whole-slide imaging, endometrial cancer, endometrial hyperplasia, endometrial carcinoma, digital slide repository, image analysis, image segmentation, histopathology, deep learning, machine learning

## Abstract

**Background:**

Whole-slide imaging enables the digitization of entire histological slides at a high resolution, allowing pathologists and researchers to analyze tissue samples digitally rather than through traditional microscopy. This technology has become increasingly valuable in pathology for research, education, and clinical diagnostics. Endometrial biopsy is very common, often being undertaken to exclude noncancerous disease. This means that most cases do not contain cancer, and the challenge is to accurately and efficiently exclude serious pathology rather than simply make a diagnosis of malignancy. A well-curated, expert-annotated, endometrial whole-slide dataset covering a spread of cancer and noncancer diagnoses will support machine learning applications in automated diagnosis, facilitate research into the pathology of endometrial cancer, and serve as an educational resource for medical professionals.

**Results:**

We introduce a newly constructed, large-scale dataset of endometrial biopsy specimens, comprising 2,909 whole-slide images in iSyntax format, each accompanied by a corresponding annotation file in JSON format. Each whole-slide image is labeled with a primary class label representing its final diagnosis and a subcategory label providing further details within that diagnostic class. These class labels are critical for machine learning applications, as they enable the development of artificial intelligence models capable of distinguishing between different types of endometrial abnormalities, improving automated classification, and guiding clinical decision-making.

**Conclusions:**

Constructing and curating a high-quality endometrial whole-slide dataset requires significant effort to ensure accurate annotations, data integrity, and patient privacy protection. However, the availability of a well-annotated dataset with detailed class labels is crucial for advancing digital pathology. Such a resource can enhance diagnostic accuracy, support personalized treatment strategies, and ultimately improve outcomes for patients with endometrial cancer and other endometrial conditions.

## Data Description

The endometrial dataset described in this article includes a total of 2,909 hematoxylin and eosin (H&E)–stained whole-slide images (WSIs) from NHS Greater Glasgow and Clyde Biorepository and Pathology with a total of 3.6 TB storage. This dataset was originally created as part of the Industrial Centre for the Industrial Centre for Artificial Intelligence Research in Digital Diagnostics (iCAIRD) [1] with the aim to automatically sort histopathology WSIs of endometrial biopsy specimens into 1 of 3 categories: “malignant,” “other or benign,” or “insufficient.” This would allow prioritization of malignant slides within the pathologists’ workload and reduce the time to diagnosis for patients with cancer.

### Context

As the demand for artificial intelligence (AI) services continues to grow, so does the need for high-quality datasets. Data are the key component of any machine learning (ML) and deep learning projects. The quality of data is as important as the quantity; hence, data preparation and understanding is one of the most important and time-consuming tasks of the ML project life cycle.

Machine learning in health care can be used for better diagnosis using ML-enabled tools to analyze medical reports and images. The use of AI in clinical practice aids pathologists in many ways. Techniques like digital image analysis and machine learning are excellent in predicting cancer outcomes. These AI models can help with pathological diagnoses and train pathologists to identify areas of interest in tissue samples.

Endometrial cancer is a type of cancer that originates in the lining of the uterus, which is called the endometrium. It is one of the most common forms of cancer that affects the female reproductive system. The endometrium is the tissue that undergoes changes throughout the menstrual cycle and is shed during menstruation.

Using AI for the detection of endometrial cancer has shown promising results in recent research and clinical applications. AI techniques, such as ML and deep learning, can be applied to medical imaging and clinical data to aid in early detection and accurate diagnosis of endometrial cancer.

A recent review of AI in gynecological cancers [2] found 13 papers for endometrial cancer, out of which only 1 paper used H&E WSIs from endometrial biopsy specimens [3]. In this study, a convolutional neural network (CNN) was trained on patches of size $640\times 640$ pixels extracted from the regions annotated by pathologists as normal or malignant. CNNs differentiated patches as endometrial adenocarcinoma and 3 benign classes (normal, endometrial polyp, and endometrial hyperplasia) and achieved 93.5% accuracy on the binary classification task and 78.0% sensitivity. The results presented in this article are at the patch level only, and no slide-level classification has been reported.

A endometrial cancer H&E slides dataset, the Clinical Proteomic Tumor Analysis Consortium (CPTAC) [4], is available from the cancer imaging archive, consisting of pathology slides along with genomics data and radiology images. The 3 studies that used CPTAC aimed to predict the same information as genetic sequencing [5] or illustrate features in H&E slides that could identify different cancer variants [6, 7] and hence allow more personalized treatment.

A weakly supervised learning method used this endometrial dataset for WSI diagnosis and interpretability. Interpretability methods, including attention heatmapping, feature visualization, and a novel end-to-end saliency mapping, were applied to identify distinct morphologies learned by the model and build an understanding of its behavior [8]. The reported results in this article show slide-level validation and test accuracies over 85% and 87%, respectively. This dataset also has been used for detecting malignancy using AI in a recent article [9]. In this article, a fully supervised CNN model was trained to automatically sort endometrial biopsy images into “malignant,” “other or benign,” or “insufficient” tissue classes with the aim to allow prioritization of these slides in a queue for pathologist review and hence reduce time to diagnosis for patients with cancer. The final model was able to accurately classify 90% of all slides correctly and 97% of slides in the malignant class; this accuracy is good enough to allow prioritization of the workload. The code and trained model for this study are available at [10].

## Methods

### Data collection

**Figure 1:**
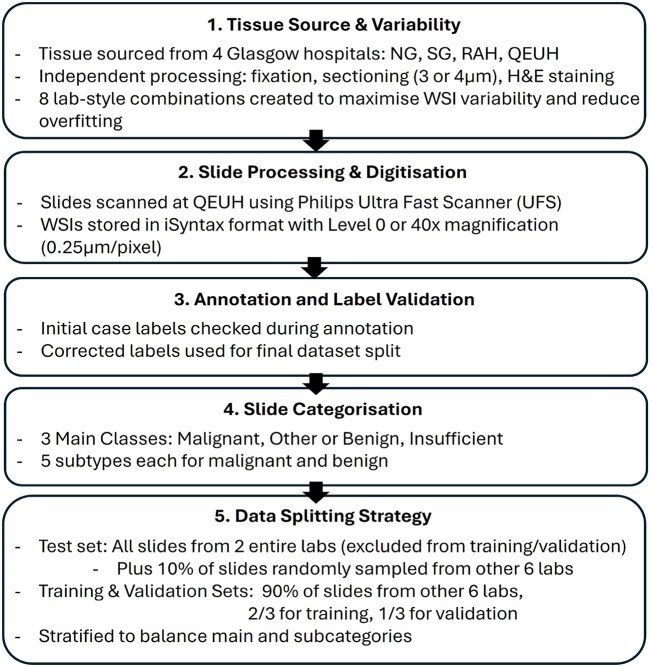
Flowchart of data collection and data split.

The tissue blocks for this study originate from the Glasgow Royal Infirmary (NG), Southern General Hospital (SG), Royal Alexandria Hospital (RAH), and Queen Elizabeth University Hospital (QEUH) (all in Glasgow, Scotland), each with independent tissue handling, including fixation and tissue processing. New tissue sections were cut from the tissue blocks at 1 of 2 different thicknesses (3 or 4 microns) and then stained with 1 of 4 different H&E protocols. Together, these combinations gave 8 different labs maximizing WSI variance and thereby decreasing the likelihood of overfitting to any 1 lab (combination of tissue processing, cutting, and staining protocol) (Fig. 1).

### Data split to train and test sets

**Figure 2:**
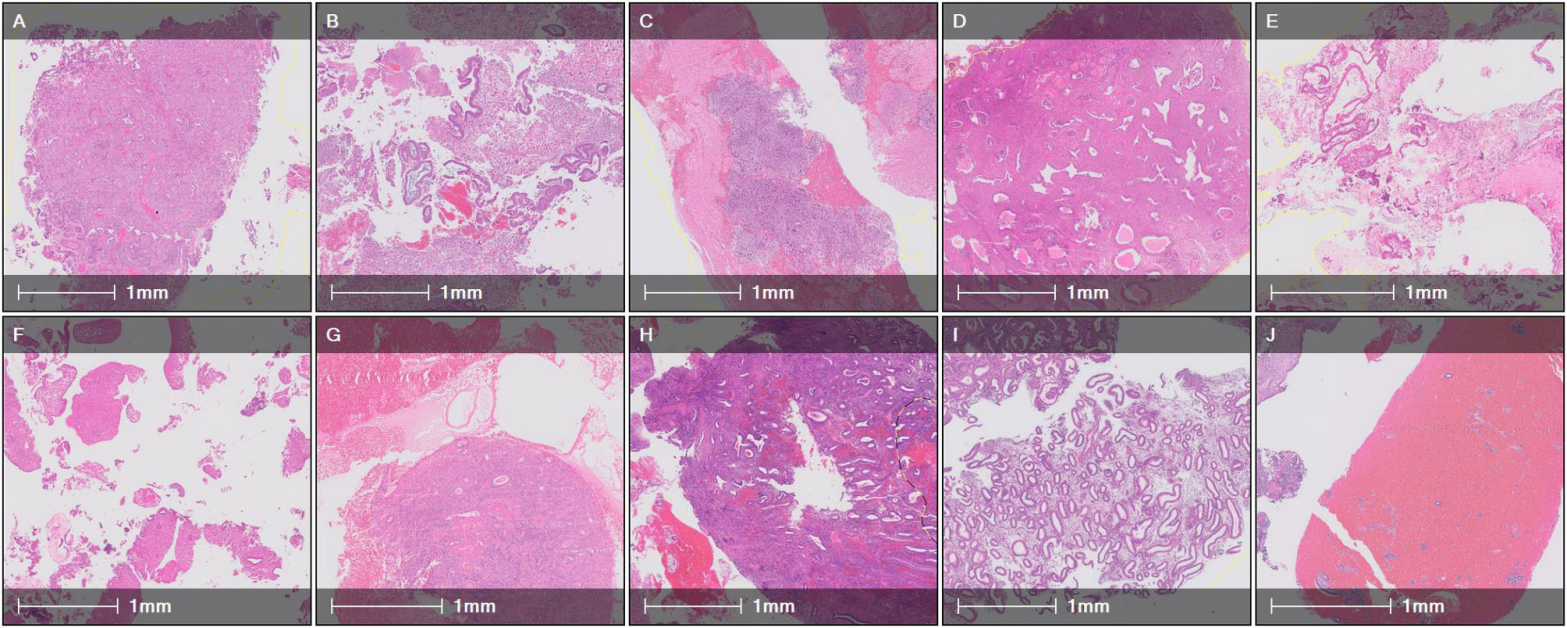
Example of subcategory of malignant and other or benign cases. (A) Malignant—adenocarcinoma. (B) Malignant—carcinosarcoma. (C) Malignant—sarcoma. (D) Malignant—hyperplasia with atypia. (E) Malignant—other. (F) Other or benign—hormonal. (G) Other or benign—inactive atrophic. (H) Other or benign—proliferative. (I) Other or benign—secretory. (J) Other or benign—menstrual.

The slides were split into training, validation, and test sets. The samples had examples of 5 “malignant” subcategories, 5 “other or benign” subcategories, and a category “insufficient,” where there was insufficient tissue to make a diagnosis. Hyperplasia with atypia was included in the “malignant” category as it is a high-risk preinvasive lesion that is important to detect (Fig. 2).

The test set contained the complete groups of slides for 2 of the labs, and these slides were not part of the training and validation sets. The test set then also contained a randomly selected 10% of the slides from the other 6 labs. The remaining 90% of the slides, from the other 6 labs, were used for the training and validation sets. Two-thirds of these slides were selected randomly for the training set, and the rest were used for the validation set. The splits into the test, validation, and training sets were checked to see that there was a balance of the categories and subcategories across the sets. These splits were calculated based on the case labels associated with the samples recorded in the system. During the annotation process, these labels were doubled checked, and in approximately 5% of the cases, the final label associated with the scanned slide was different. This could be because the new slice taken from the sample did not show the same pathology as the original or that the original label was incorrectly recorded. The corrected labels postannotation were the labels that were used for training and testing. This means the final numbers of slides of each type may not match the original percentages described above. The distribution of data over train, validation, and test sets is shown in Table 1.

**Table 1:** Distribution of samples in training, validation, and test sets for the endometrial dataset

Category	Subcategory	Training	Validation	Test	Total
Malignant	Adenocarcinoma	243	113	162	518
	Carcinosarcoma	37	18	28	83
	Sarcoma	11	6	8	25
	Hyperplasia with atypia	106	53	67	226
	Other	4	1	3	8
Total		401	191	268	860
Other or benign	Hormonal	158	79	115	352
	Inactive atrophic	170	90	133	393
	Proliferative	184	81	116	381
	Secretory	176	91	116	383
	Menstrual	159	84	111	354
Total		847	425	595	1,867
Insufficient	Insufficient	90	44	48	182

All slides were then scanned at QEUH and saved as WSIs. The WSIs are hundreds of thousands of pixels in height and width at the highest magnification and are too large to read into memory. Dedicated WSI formats allow access to either small parts of the image at the highest magnification or the whole image at lower magnifications. For this study, slides were scanned using a Philips Ultra Fast Scanner and stored in the iSyntax file format. The most detailed view in the WSI is level 0, or 40× magnification, where the length of a side of 1 pixel in the image is 0.25 $\mathrm{\mu }$m. Higher levels represent lower magnifications in a pyramid, where each level is a power of 2 smaller than the previous (Fig. 1).

### Annotation process

The scanned slides were annotated by a mix of experienced biomedical scientists and pathologists from NHS Greater Glasgow and Clyde. The work of the biomedical scientists was reviewed and approved by a pathologist before use. The annotations took place using the QuPath software [11], and the isyntax [12] files were converted to OME-Tiff files using a Glencoe software converter [13] prior to annotation. The code utilized for this conversion is publicly available from Zenodo [10].

**Figure 3:**
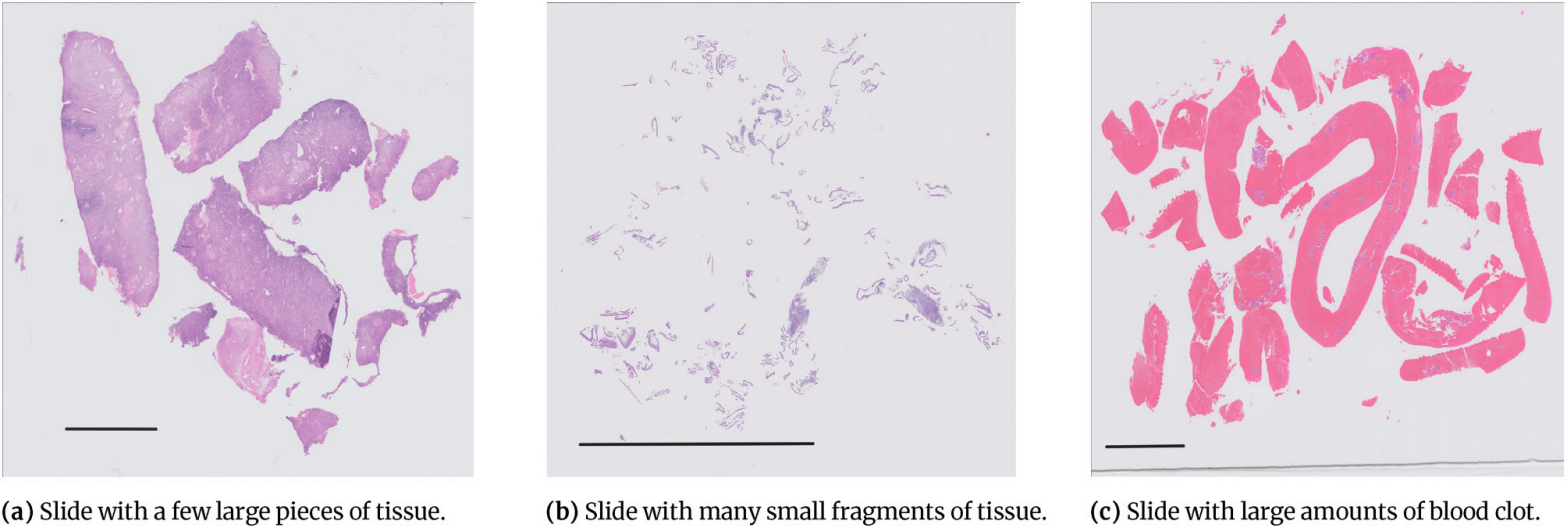
Examples of slides with different amounts and presentation of tissue. Scale bar = 5 mm.

Annotation of endometrial slides is complicated due to the structure of the tissue present on the slides. Some of the slides contained a small number of large contiguous pieces of tissue (Fig. 3A), where only annotating the malignant areas is straightforward. However, some of the slides contained a very large number of small fragments of tissue (Fig. 3B). These slides would require the pathologists to annotate separately many small bits of tissue on slides where nearly all the tissue was malignant. In addition, some slides contained a very large amount of blood or mucus with no diagnostic value (Fig. 3C). Therefore, it was decided that annotating blood and mucus either as a separate class or as part of the “other or benign” class would be time prohibitive, and an alternative approach was needed [9].

The widely used method for annotating H&E slides takes the approach that only the area of interest is annotated, and the rest of tissue is considered normal tissue and therefore is not annotated. Due to the structural complexity of the endometrial slides mentioned above, it was decided to take a different approach for annotating these slides.

The annotation approach taken for endometrial WSIs gives an overall class to the slide, and then to only annotate parts of the slide that differed from the overall class. The classes used for annotation were “malignant” and “other or benign.” Slides categorized as “insufficient” lack specific tissue types, so this term was not used as an annotation class. Annotators were not required to denote the areas of tissue on the slide as tissue detection was applied as part of the preprocessing algorithm. Hence, a large number of the annotation files were blank as everything on the slide was from the overall class with no other annotation required.

**Figure 4:**
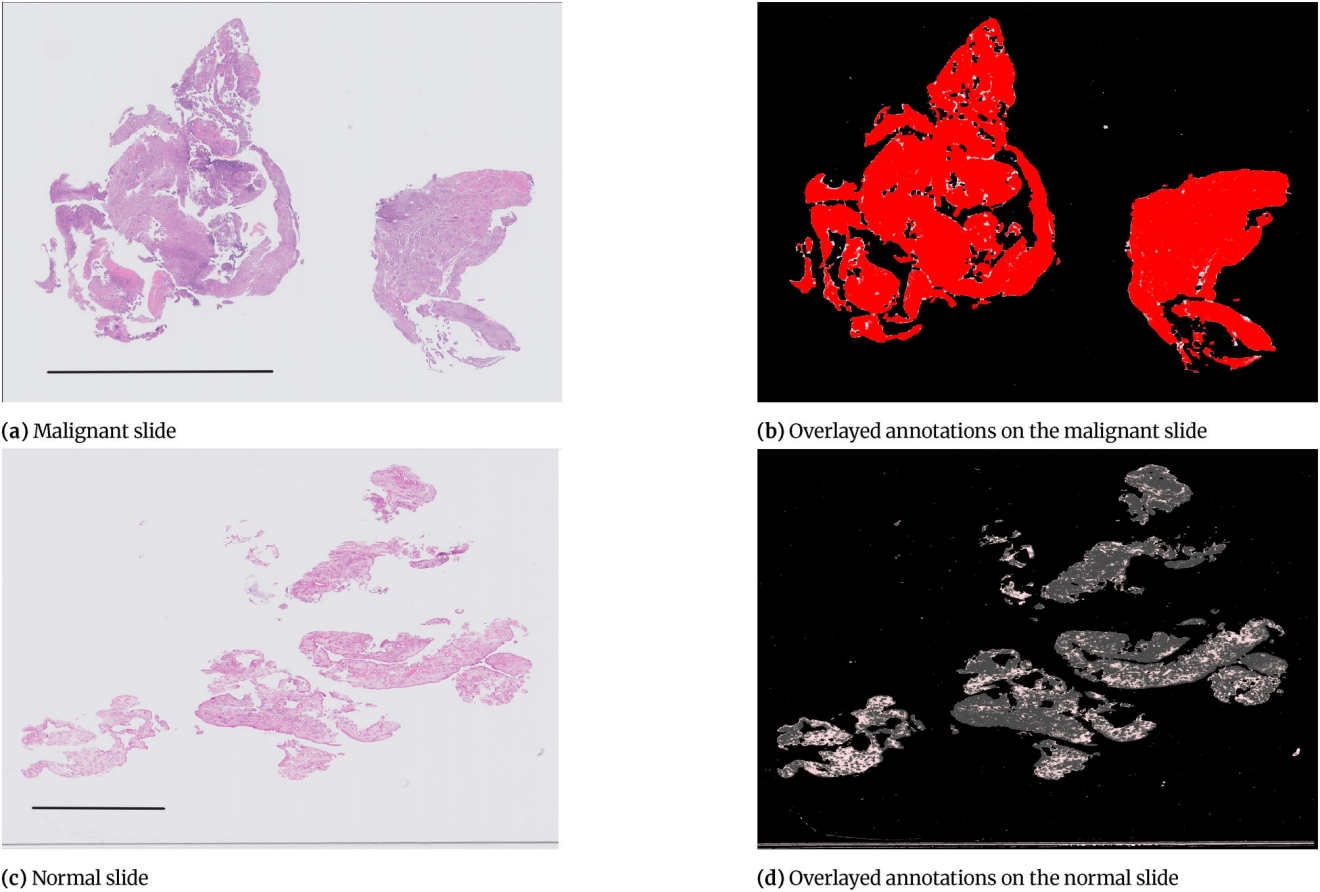
Examples of slides where all tissue on the slide is of the same category. Scale bar = 5 mm. 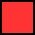 Malignant. 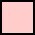 Blood or mucus. 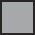 Normal tissue. 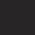 Background.

Figure 4 shows examples of endometrial slides where all the tissue on the slide is of the overall category assigned to that slide, and therefore the annotation files for them are blank as no annotation was needed for them. Tissue detection or background separation and blood and mucus detection are then applied to the slide in the preprocessing stage. To detect the tissue and separate it from the background, a thumbnail image of the slide at level 5 is created. Figure 3C shows how multiple tissue areas are saved as separate images in iSyntax format to reduce the file size. In the thumbnails, the missing areas between these images are pure black pixels. Any pixels in the thumbnail that are pure black are converted to pure white. The image is then converted to grayscale, and as the background is predominately white, any values of greater than 0.85 are considered background. Next, a closing transform and a hole-filling morphological operation are applied; the operations improve the amount of tissue captured around edges and holes. The mask created by the tissue detection algorithm for the slide shown in Fig. 4A is shown in Fig. 4C. When tissue detection is combined with the annotation (Fig. 4b), it gives the areas of the slide as “malignant” or “other or benign” tissue, as shown in Fig. 4D [9].

**Figure 5:**
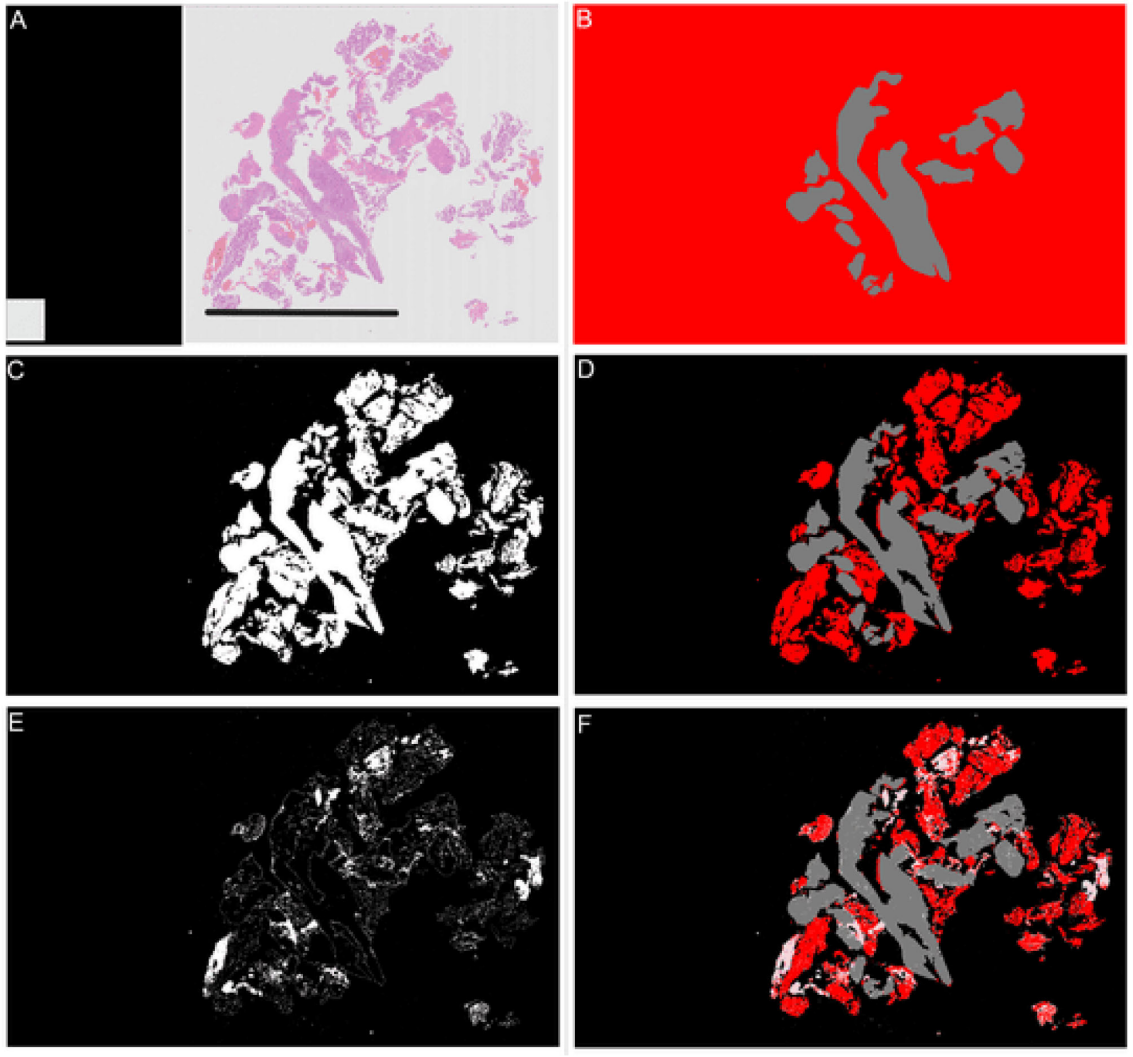
Examples of all stages in slide annotation and detection of tissue. Scale bar = 5 mm. (A) Thumbnail of “malignant” slide where some tissue is “other or benign.” (B) Annotation for “malignant” slide where some tissue is “other or benign.” (C) Mask showing areas detected as tissue in white; background is shown in black. (D) Combined annotation and tissue detection. (E) Calculated mask showing areas detected as “blood or mucus” in white; anything that is not “blood or mucus” is shown as black. (F) Combined annotation, tissue, and “blood or mucus” detection. 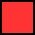 Malignant. 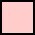 Blood or mucus. 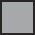 Normal tissue. 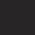 Background.

The second stage is to identify any blood or mucus on the slide. Blood and mucus detection is carried out on a pixel-by-pixel basis. Each of the red, green, and blue (RGB) channels is considered separately. A Gaussian filter with a kernel size of 2 is applied. Then a texture filter is applied to each channel both with and without the Gaussian filter to give a total of 12 different features for each pixel (raw pixel value, Gaussian filtered value, texture filter on raw, and texture filter on Gaussian filter for each of 3 channels). A random forest model was trained using a small subset of images with detailed annotations to determine the difference between “blood or mucus” and “tissue” pixels. The trained blood and mucus detection model was then applied to each image to identify “blood or mucus.” For the slide shown in Fig. 5A, the areas detected as “blood or mucus” are shown in Fig. 5E. When this is combined with the tissue detection and annotations, it gives the areas of the slide as “malignant” or “other or benign,” as shown in Fig. 5 [9].

**Figure 6:**
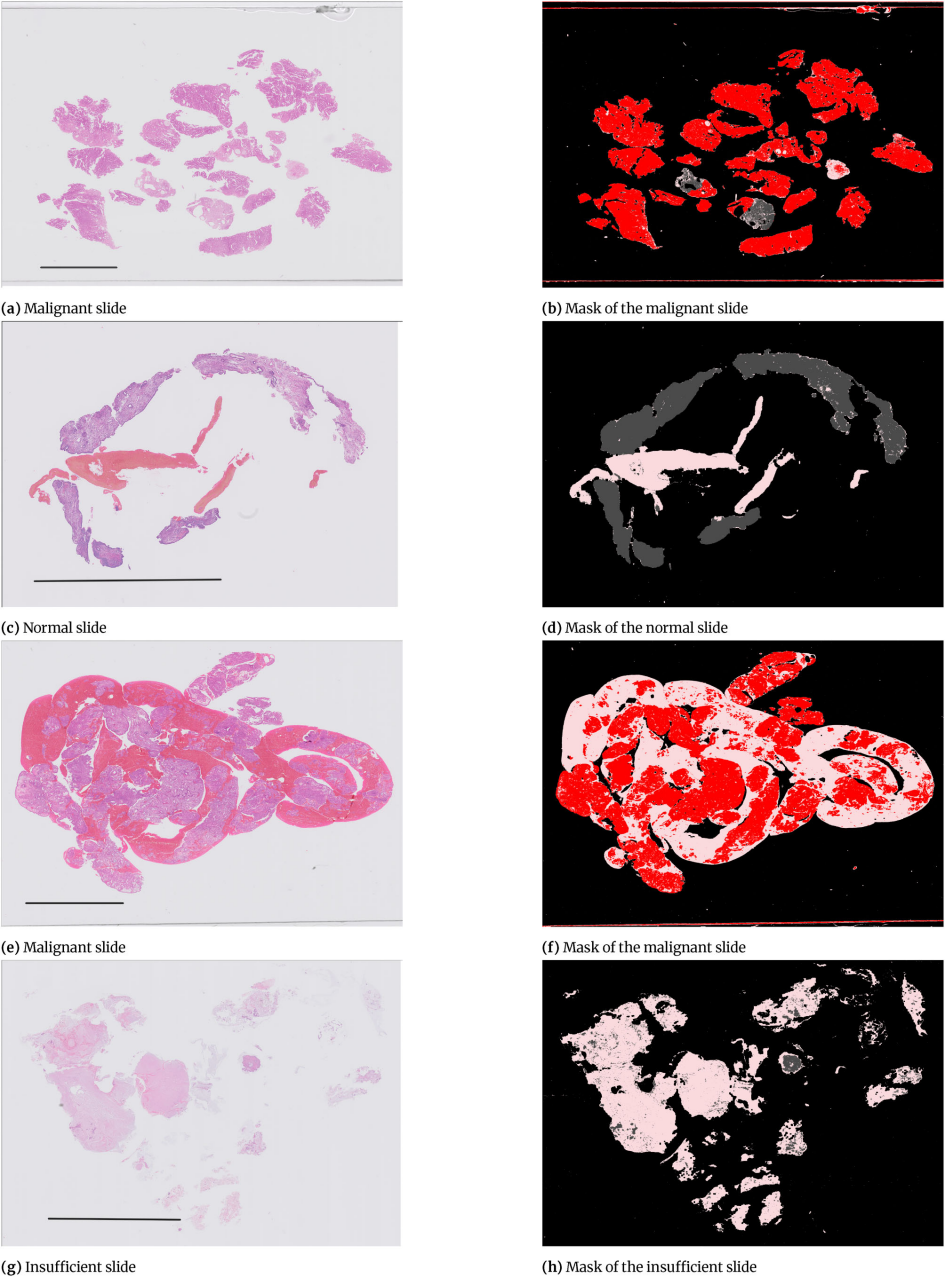
Examples of slides and their masks after applying overlaying annotations and applying tissue and blood or mucus detection stages to the slide. Scale bar = 5 mm. 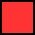 Malignant. 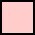 Blood or mucus. 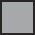 Normal tissue. 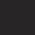 Background.

Figure 6 shows examples of slides where different categories are present on the slide. In these examples, the background and blood and mucus area are detected later in tissue detection and blood or mucus detection stages.

### Interobserver variability in annotation

Disagreements can occur among pathologists when categorizing slides. To assess this variability, 3 pathologists independently annotated a subset of 295 test slides. Their agreement was measured using Cohen’s κ statistic, with the arithmetic mean of all observer pairs reported [9]. Despite strong concordance, some inconsistencies were observed. The most frequent category-level disagreements were between “insufficient” and “other benign,” while at the subcategory level, differences arose mainly between “insufficient” and “inactive/atrophic,” as well as “hyperplasia with atypia” and “adenocarcinoma” within the malignant class. These disagreements are visualized in the confusion matrices [9].

## Data Validation and Quality Control

The images submitted were obtained directly from cases undergoing clinical histopathological diagnosis and were subject to rigorous scrutiny by the specialist team of diagnostic histopathologists who undertook the manual annotations of selected features. The annotations were added afterward as a separate exercise, not linked to clinical diagnosis. The gold standard was the pathologists’ diagnosis and, where there was discrepancies, by consensus review.

Using the H&E endometrial WSI dataset and their annotations, ML algorithms can be applied to assist in various aspects of cervical health analysis. Data collection and preprocessing is the first step in illustrating how ML algorithms can utilize these data.

### Extracting nuclear morphological features using Indica Halo AI

**Figure 7:**
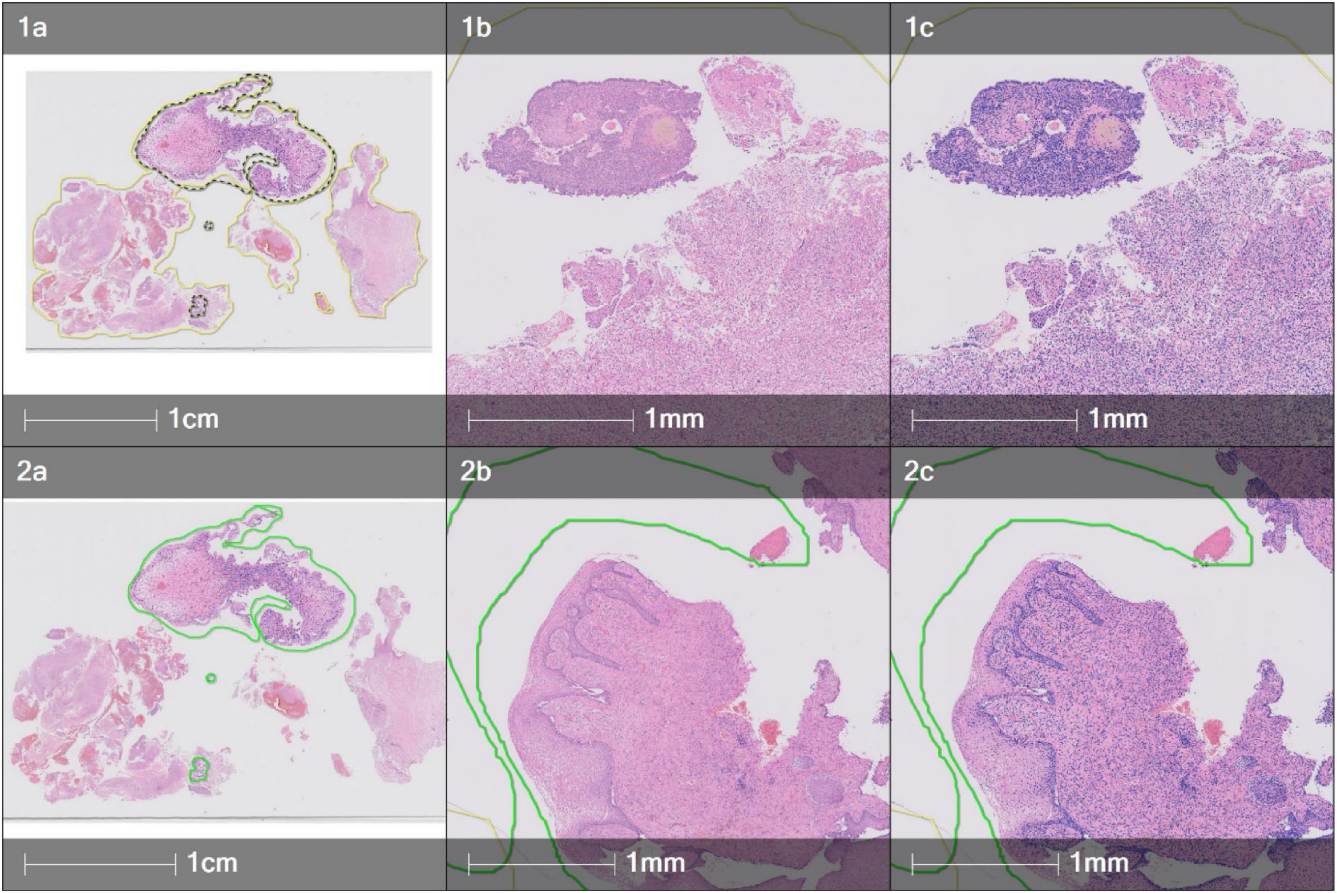
Example of segmented nuclei (colored blue) in 2 different annotations in the same patient using the Indica HALO AI platform. (1a) Annotation of the malignant area (yellow line), having excluded the normal cervix (dotted yellow annotation). (2a) Annotation of normal cervix (green line). (1b, 2b) Higher magnification of 1a and 2a, respectively. (1c, 2c) Multiplex immunohistochemistry analysis algorithm was used to segment individual nuclei (blue nuclei mask) and to extract their morphological features within annotations.

WSI images were imported into Indica HALO and HALO AI (v.3.6.4134), along with corresponding annotation files created in QuPath by pathologists. A nuclei segmentation classifier, underpinned by advanced deep learning neural network algorithms, was trained with examples from multiple different cases (Fig. 7). An analysis algorithm, Multiplex IHC v.3.2.3, was utilized to segment individual nuclei to extract nuclear morphological features such as area, perimeter, and roundness within the annotation (Fig. 5). The tabular data from the individual nuclear morphological features, along with their x and y coordinates, was exported into CSV file format.

## Reuse potential

The endometrium can display a wide range of histological appearances with overlapping features, which makes the diagnosis of various lesions complex and specifically distinguishing between premalignant and malignant conditions challenging. The diverse presentation of symptoms of endometrial abnormalities may be attributed to different underlying conditions, making accurate diagnosis based solely on clinical presentation impossible. This dataset includes a wide range of endometrial WSIs containing a wide spectrum of histological conditions that have been annotated by pathologists and can be used for training AI-based algorithms to identify the endometrial abnormalities and to detect slides with malignant tissue to allow prioritization of these slides in a queue for pathologist review and hence reduce time to diagnosis for patients with cancer. Moreover, nuclear morphological features such as area, perimeter, and roundness may enhance accuracy in distinguishing between premalignant and malignant. Furthermore, one problem in diagnostic pathology is how to deal with small samples that may not be representative or provide conclusive evidence for the pathologist. On occasion, this may necessitate a report that states “tissue insufficient for diagnosis,” which in turn may lead to a further sample being sought. This is a potential distressing and painful procedure for the patient. Being more confident about defining an inadequate sample will help workflow, ensure safety, and minimize unnecessary discomfort to the patient.

This dataset was utilized to generate 3 models distinguishing 3-class classification, “malignant,” “other or benign,” and “insufficient,” WSIs [9]. Among these, the CNN achieved the highest accuracy in identifying malignant cases, with classification accuracy ranging from 89.8% to 92.1% depending on whether any tissue patches or majority-tissue patches were used. However, its overall accuracy (85.2%–90.8%) was slightly lower than that of the random forest model, which yielded the highest overall accuracy but underperformed in correctly identifying malignant cases. XGBoost provided a balanced performance, intermediate between the CNN and random forest classifiers. These results, presented in detail in the original publication [9], offer a benchmark for future method development and validation.

## Ethical Approval

Ethics approval for the study was granted by NHS Greater Glasgow and the Clyde Biorepository and Pathology Tissue Resource (REC reference 16/WS/0207) on 4 April 2019. Biorepository approval was obtained (application number 511).

Local approval was obtained from the School of Computer Science Ethics Committee, acting on behalf of the University Teaching and Research Ethics Committee (UTREC) [approval code CS15840].

## Abbreviations

AI: artificial intelligence; CPTAC: Clinical Proteomic Tumor Analysis Consortium; CNN: convolutional neural network; H&E: hematoxylin and eosin; iCAIRD: Industrial Centre for Artificial Intelligence Research in Digital Diagnostics; ML: machine learning; NG: Glasgow Royal Infirmary; QEUH: Queen Elizabeth University Hospital; RAH: Royal Alexandria Hospital; SG: Southern General Hospital; TB: tetabytes; WSI: whole-slide image.

## Competing Interests

The authors declare that they have no competing interests.

## Funding

This work is supported by the Industrial Centre for AI Research in digital Diagnostics (iCAIRD), which is funded by Innovate UK on behalf of UK Research and Innovation (UKRI) [project number: 104690] and in part by Chief Scientist Office, Scotland.

## Author Contributions

M.M. wrote the manuscript and supervised data preprocessing, together with C.F. and I.H.U. P.S. imported annotations in Indica Halo AI platform and measured nuclear morphological features. G.B. initiated the project, and S.B., S.S., and P.K. annotated the whole-slide images. D.H.B. and O.A. supervised machine learning experiments. C.O. arranged data release from Glasgow Biorepository. J.B. oversaw governance procedures, established digital pathology services, and supervised data deidentification and release. D.H. obtained funding, reviewed results, and helped to draft the manuscript. All authors have seen and approved the manuscript.

## Supplementary Material

giaf147_Authors_Response_To_Reviewer_Comments_Original_Submission

giaf147_Authors_Response_To_Reviewer_Comments_Revision_1

giaf147_GIGA-D-24-00211_Original_Submission

giaf147_GIGA-D-24-00211_Revision_1

giaf147_GIGA-D-24-00211_Revision_2

giaf147_Reviewer_1_Report_Original_Submission Sonali Andani -- 7/8/2024

giaf147_Reviewer_2_Report_Original_Submission Liansheng Wang -- 8/3/2024

giaf147_Reviewer_2_Report_Revision_1 Liansheng Wang -- 4/4/2025

## Data Availability

All endometrial whole-slide images and their annotation files, binary masks, and a metadata file (2,909 images in iSyntax format, 2,909 annotation files in JSON format, 2,909 binary masks in PNG format, and a metadata file in CSV format) and the morphological features extracted from them in Halo are openly available in the BioImage Archive [S-BIAD1199] [14].
