# Supplementary material for: Endometrial whole-slide images dataset for detection of malignancy in endometrial biopsies
Source: Gigascience. 2025 Dec 5;14:giaf147. doi: 10.1093/gigascience/giaf147 (PMC12751089; doi:10.1093/gigascience/giaf147)
Supplement: giaf147_GIGA-D-24-00211_Revision_1 [file giaf147_giga-d-24-00211_revision_1.pdf]

GigaScience

Endometrial Whole Slide Images Dataset for Detection of malignancy in endometrial biopsies

--Manuscript Draft--

Manuscript Number:	GIGA-D-24-00211R1
Full Title:	Endometrial Whole Slide Images Dataset for Detection of malignancy in endometrial biopsies
Article Type:	Data Note
Funding Information:	Innovate UK (104690) — Prof David James Harrison
Abstract:	Background: Whole slide imaging (WSI) enables the digitisation of entire histological slides at high resolution, allowing pathologists and researchers to analyse tissue samples digitally rather than through traditional microscopy. This technology has become increasingly valuable in pathology for research, education, and clinical diagnostics. Endometrial biopsy is very common, often being undertaken to exclude non-cancerous disease. Thismeans thatmost cases do not contain cancer, and the challenge is to accurately and efficiently exclude serious pathology rather than simply make a diagnosis of malignancy. A well-curated, expert-annotated, endometrial whole slide dataset covering a spread of cancer and non-cancer diagnoses will support machine learning applications in automated diagnosis, facilitate research into the pathology of endometrial cancer, and serve as an educational resource formedical professionals. Results: We introduce a newly constructed, large-scale dataset of endometrial biopsies, comprising 2,909 whole slide images in iSyntax format, each accompanied by a corresponding annotation file in JSON format. Each whole slide image is labelled with a primary class label representing its final diagnosis and a sub-category label providing further details within that diagnostic class. These class labels are critical for machine learning applications, as they enable the development of AImodels capable of distinguishing between different types of endometrial abnormalities, improving automated classification, and guiding clinical decision-making. Conclusions: Constructing and curating a high-quality endometrial whole slide dataset requires significant effort to ensure accurate annotations, data integrity, and patient privacy protection. However, the availability of a well-annotated dataset with detailed class labels is crucial for advancing digital pathology. Such a resource can enhance diagnostic accuracy, support personalized treatment strategies, and ultimately improve outcomes for patients with endometrial cancer and other endometrial conditions.
Corresponding Author:	In Hwa Um, Ph.D.<br>St Andrews University<br>St Andrews, Scotland UNITED KINGDOM
Corresponding Author Secondary Information:	
Corresponding Author's Institution:	St Andrews University
Corresponding Author's Secondary Institution:	
First Author:	In Hwa Um, Ph.D.
First Author Secondary Information:	
Order of Authors:	In Hwa Um, Ph.D.
	Mahnaz Mohammadi
	Christina Fell
	Sarah Bell
	Gareth Bryson
	Sheeba Syed
	Prakash Konanahalli

Powered by Editorial Manager® and ProduXion Manager® from Aries Systems Corporation

	David Harris-Birtill
	Ognjen Arandjelovic
	Clare Orange
	Prishma Shahi
	David James Harrison
	James D Blackwood
**Order of Authors Secondary Information:**	

Response to Reviewers:

Reviewer #1:

1.File format: The most common formats for WSIs are ".svs" and ".tif" and the most common python library used is "OpenSlide". Openslide does not support "isyntax" file format (reference: https://eur01.safelinks.protection.outlook.com/?url=https%3A%2F%2Fopenslide.org%2Fapi%2Fpython%2F&data=05%7C02%7Cihu%40st-andrews.ac.uk%7C0510f47c971843087ded08dcb5449a05%7Cf85626cb0da849d3aa5864ef678ef01a%7C0%7C0%7C638584553449646841%7CUnknown%7CTWFpbGZsb3d8eyJWIjoiMC4wLjAwMDAiLCJQIjoiV2luMzIiLCJBTiI6Ik1haWwiLCJXVCI6Mn0%3D%7C0%7C%7C%7C&sdata=u4y5wFU52ot0TNyDujqU%2BzIinmlKEptlzOQLcH7e9dg%3D&reserved=0). In order to make sure the EC dataset reaches a vast audience, it is recommended to convert to one of the file formats supported by OpenSlide library.

Glencoe software was used to convert iSyntax format to ome-tiff, as described in the text. More information on this process can be found at the following link: https://www.glencoesoftware.com/blog/2019/12/09/converting-whole-slide-images-to-OME-TIFF.html
In preliminary discussions with Dr. Armit before submission, we acknowledged that while the iSYNTAX format can pose certain challenges, it is still possible to convert these images successfully. Additionally, some researchers seek access to Philips-originated images to address and overcome the relative difficulty of working with this format. This highlights the importance of maintaining accessibility to such datasets, as they contribute to the broader goal of improving interoperability and image analysis in the research community.

2.Metadata: In order for the community to use this dataset, it would be important to include metadata such as age, survival data, stage, response to treatment, if any treatment given, molecular class or any available metadata. For reference, the authors could use the metadata available for TCGA datasets as a reference.

We do not have this information. Sex is obviously female, but in almost every case included the diagnosis would have no consequences for long term clinical outcome. The nature of the conditions being diagnosed and studies means that no molecular genetic testing would be carried out routinely. TCGA has detailed studies of cancer, but many of the cases in our cohort are not cancerous, which is precisely why we believe this is a useful, unique addition to publicly available data.

3.Highlighting the utility of datasets: A missing piece in the manuscript is to showcase the real utility of the dataset. This could include comparison with other publicly available EC datasets. What does the model performance look like in terms of metrics such as F1 score, precision, recall. If the nuclei features are included, what would the performance look like.

Preliminary results can be found in our cited primary research paper (Fell et al, “Detection of malignancy in whole slide images of endometrial cancer biopsies using artificial intelligence”, https:// doi.org/10.1371/journal.pone.0282577). In this study, we developed and reported an AI model designed to assist in the classification of whole slides images. The model categorises slides into, “malignant, “other or benign” and “insufficient” at the slide level. Additionally, the AI generates heatmaps to highlight specific regions withing the slides that are likely malignant, providing valuable insights for pathologists.
We have not directly compared the performance differences between this AI model

and nuclear morphological features. However, we believe that referencing this paper, along with the additional tabular data on nuclear morphological features in reference 14 (https://www.ebi.ac.uk/biostudies/bioimages/studies/S-BIAD1199?query=endometrial), should offer valuable insights since this is the only large study with histological images of endometrial biopsies. These resources can help researchers using the dataset to design their own analyses, formulate hypotheses, and conduct experiments to explore potential relationships between AI-driven classification and nuclear morphology-based assessment.
Regarding model performance metrics, they are detailed in the publication. The study provides a baseline evaluation of the model’s diagnostic capabilities, which can serve a foundation for future comparative analyses with other publicly available endometrial cancer (EC) datasets.

4.Data distribution: It would be good if authors provided the class label and data split for each of the eight different labs.
This table shows the data split for eight different laboratories.

StainingSiteIDcategoprytrainvalidtest
1other_benign1576726
1malignant733110
1insufficient1292
2other_benign1467426
2malignant723012
2insufficient2180
3other_benign1447525
3malignant613210
3insufficient1752
4other_benign1387823
4malignant743712
4insufficient1483
5other_benign1367023
5malignant633310
5insufficient1372
6other_benign00222
6malignant00107
6insufficient0021
7other_benign1336722
7malignant642911
7insufficient1682
8other_benign00236
8malignant00100
8insufficient0018

5.In case authors don't already plan to do it, it would be good if the trained blood and mucus detection model is also made available.

The complete process is comprehensively described in Fell et al. which we have cited, “Detection of malignancy in whole slide images of endometrial cancer biopsies using artificial intelligence”, https:// doi.org/10.1371/journal.pone.0282577. Additionally, the relevant codes supporting this work are publicly available on Zenodo (https://zenodo.org/record/7674764), and this is cited in reference 10.

6.Table 1 currently includes the distribution of samples. Since one patient can possibly have more than one sample, it would be good to include distribution of patients as well.

There is only one sample per patient.

7.Under "annotation process", the authors mention a mix of experts were used for annotations. How many annotators were exactly used for the task?

Four histopathologically trained biomedical scientists and four histopathologists annotated biopsies. All histopathologists who annotated biopsies are registered with GMC and Royal College of Pathologists, with subspecialty expertise in gynaecological pathology. Each participating pathologist has a minimum of 5-10 years of post-

Powered by Editorial Manager® and ProduXion Manager® from Aries Systems Corporation

specialist training experience, with some having over 15 years of expertise in gynaecological pathology. To ensure high-quality and standardised annotations, pathologists reviewed a detailed annotation protocol based on internationally recognised guidelines (WHO classification of tumours, 5th Edition).

8.Abstract: The abstract could highlight the class labels and their importance

Abstract has been improved as requested.

9.It would be good to add "scale" example for 1mm for all images in figures 1-4.
It has been done as requested.

Reviewer #2:

10.A detailed summary of existing databases related to endometrial cancer should be provided, including but not limited to the TCGA UCEC. Specifically, it is important to highlight the advantages or contributions of the database mentioned in this paper compared to the existing datasets.
Our primary research paper (Fell et al, “Detection of malignancy in whole slide images of endometrial cancer biopsies using artificial intelligence”, https://doi.org/10.1371/journal.pone.0282577) discusses a publicly available endometrial cancer histology image dataset (https://doi.org/10.7937/k9/tcia.2018.3r3juisw). However, this comprises only 250 cases, whereas our dataset has 2909 cases. There is no comparable dataset covering a wide range of endometrial pathology available as far as we know.
11.It would be beneficial to present the data collection procedure and data split details using a flow chart or a table. In the current manuscript, these two sections are not easy to understand.

Figure has been added as requested.

12.More details about this database should be provided. For instance, include information on the distribution of the proportion of annotated areas in the entire slide, as well as the racial, gender, and age distribution of the whole slide images.
The tissue on the slide has been fully annotated by expert pathologists. Patients were obviously female and predominantly Caucasian, but the ethics permission does not include identifiers such as age and race.
13.In Table 1, please clarify what the subcategory “Other” refers to. Additionally, explain the potential uses for slides labelled as “insufficient”.
One problem in diagnostic pathology is how to deal with small samples that may not be representative or provide conclusive evidence for the pathologist. On occasion this may necessitate a report that states “tissue insufficient for diagnosis”, which in turn may lead to a further sample being sought. This is a potential distressing and painful procedure for the patient. Being more confident about defining an inadequate sample will help workflow, ensure safety and minimise unnecessary discomfort.
14.It would be better to explain what deep learning tasks this database can be utilized for, such as patch classification, whole slide image classification and segmentation. Additionally, briefly describe how to process the annotations for each corresponding task.
The database can be used for whole slide image classification or patch classification. Some slides contain a mix of classes and can be used for segmentation.
Section 2.3 of our primary research paper (Fell et al, “Detection of malignancy in whole slide images of endometrial cancer biopsies using artificial intelligence”, https://doi.org/10.1371/journal.pone.0282577) which is cited as reference 9 describes how to process annotations.
15.To validate the proposed database, benchmark results should be provided. For example, patch classification results using CNN models and WSI classification results using a multiple instance learning model.
This was described in the original research paper (Fell et al, “Detection of malignancy in whole slide images of endometrial cancer biopsies using artificial intelligence”, https://doi.org/10.1371/journal.pone.0282577).

16.It would be better to show typical regions for each subcategory and briefly summarize their differences.

Powered by Editorial Manager® and ProduXion Manager® from Aries Systems Corporation

	Figure has been added as requested.<br><br>17.In Table 1, the largest number of these subcategory has 518 slides, while the smallest number is only 8. The authors should explain why the number of WSIs in each category is unbalanced.<br>The cases were obtained from a biorepository and reflect the mix of conditions received by the laboratories. Rare things are rare, and so the number of cases is limited! The preponderance of common conditions lends itself to our argument in new abstract that a major challenge in dealing with rising workload in endometrial pathology is filtering out common conditions with confidence rather than necessarily focussing on diagnosing rare conditions.
**Additional Information:**	
**Question**	**Response**
Are you submitting this manuscript to a special series or article collection?	No
**Experimental design and statistics**<br><br>Full details of the experimental design and statistical methods used should be given in the Methods section, as detailed in our Minimum Standards Reporting Checklist. Information essential to interpreting the data presented should be made available in the figure legends.<br><br>Have you included all the information requested in your manuscript?	Yes
**Resources**<br><br>A description of all resources used, including antibodies, cell lines, animals and software tools, with enough information to allow them to be uniquely identified, should be included in the Methods section. Authors are strongly encouraged to cite Research Resource Identifiers (RRIDs) for antibodies, model organisms and tools, where possible.<br><br>Have you included the information requested as detailed in our Minimum Standards Reporting Checklist?	Yes
**Availability of data and materials**	Yes

All datasets and code on which the conclusions of the paper rely must be either included in your submission or deposited in publicly available repositories (where available and ethically appropriate), referencing such data using a unique identifier in the references and in the “Availability of Data and Materials” section of your manuscript.<br><br>Have you have met the above requirement as detailed in our Minimum Standards Reporting Checklist?	

LaTeX - Main Document (TeX file)

Click here to access/download;LaTeX - Main Document (TeX file);main endo final.tex

```
This is pdfTeX, Version 3.141592653-2.6-1.40.26 (TeX Live 2024)
(preloaded format=pdflatex 2024.8.2)  14 FEB 2025 04:38
entering extended mode
 restricted \write18 enabled.
 %&-line parsing enabled.
**"main endo final.tex"
(./main endo final.tex
LaTeX2e <2024-06-01> patch level 2
L3 programming layer <2024-05-27>

! LaTeX Error: File `oup-contemporary.cls' not found.

Type X to quit or <RETURN> to proceed,
or enter new name. (Default extension: cls)

Enter file name:
! Emergency stop.
<read *>

l.11 ^^M

*** (cannot \read from terminal in nonstop modes)


Here is how much of TeX's memory you used:
 19 strings out of 473583
 490 string characters out of 5732343
 1925908 words of memory out of 5000000
 23012 multiletter control sequences out of 15000+600000
 558069 words of font info for 36 fonts, out of 8000000 for 9000
 1141 hyphenation exceptions out of 8191
 19i,0n,29p,108b,17s stack positions out of
10000i,1000n,20000p,200000b,200000s
!  ==> Fatal error occurred, no output PDF file produced!
```

Manuscript

Click here to access/download;Manuscript;Endometrial_Data_Note_GigaSci

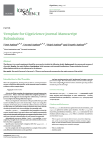

GigaScience, 20xx, 1–8

doi: xx.xxxx/xxxx
Manuscript in Preparation
Data Note

DATA NOTE

Endometrial Whole Slide Images Dataset for Detection of malignancy in endometrial biopsies

Mahnaz Mohammadi[1,*,§], Christina Fell[1,*], Sarah Bell[2,†], Gareth Bryson[2,†], Sheeba Syed[2,†], Prakash Konanahalli[2,†], David Harris-Birtill[4,*], Ognjen Arandjelovic[4,§], Clare Orange[1,2,*], Prishma Shahi[1,*], In Hwa Um[1,3,§], James D Blackwood[1,*] and David J Harrison[1,3,*]

[1]School of Medicine, University of St Andrews, North Haugh, KY16 9TF, United Kingdom and [2]Department of Pathology, Queen Elizabeth University Hospital, Govan Road, G51 4TF, Glasgow, United Kingdom and [3]Pathology, Division of Laboratory Medicine, Royal Infirmary of Edinburgh, Old Dalkeith Road, EH16 4SA, United Kingdom and [4]School of Computer Science, University of St Andrews, North Haugh, KY16 9SX, United Kingdom

[*]mm459, cmf21, dcchb, oa7, celo1, ps289, ihu, jdb20, david.harrison@st-andrews.ac.uk
[†]Sarah.Bell, Gareth.Bryson, sheeba.syed, prakash.konanahalli@ggc.scot.nhs.uk
[§]Corresponding author: mahnaz.mohammadi0@gmail.com, ihu@st-andrews.ac.uk

Abstract

Background: Whole slide imaging (WSI) enables the digitisation of entire histological slides at high resolution, allowing pathologists and researchers to analyse tissue samples digitally rather than through traditional microscopy. This technology has become increasingly valuable in pathology for research, education, and clinical diagnostics. Endometrial biopsy is very common, often being undertaken to exclude non-cancerous disease. This means that most cases do not contain cancer, and the challenge is to accurately and efficiently exclude serious pathology rather than simply make a diagnosis of malignancy. A well-curated, expert-annotated, endometrial whole slide dataset covering a spread of cancer and non-cancer diagnoses will support machine learning applications in automated diagnosis, facilitate research into the pathology of endometrial cancer, and serve as an educational resource for medical professionals. **Results:** We introduce a newly constructed, large-scale dataset of endometrial biopsies, comprising 2,909 whole slide images in iSyntax format, each accompanied by a corresponding annotation file in JSON format. Each whole slide image is labelled with a primary class label representing its final diagnosis and a sub-category label providing further details within that diagnostic class. These class labels are critical for machine learning applications, as they enable the development of AI models capable of distinguishing between different types of endometrial abnormalities, improving automated classification, and guiding clinical decision-making. **Conclusions:** Constructing and curating a high-quality endometrial whole slide dataset requires significant effort to ensure accurate annotations, data integrity, and patient privacy protection. However, the availability of a well-annotated dataset with detailed class labels is crucial for advancing digital pathology. Such a resource can enhance diagnostic accuracy, support personalized treatment strategies, and ultimately improve outcomes for patients with endometrial cancer and other endometrial conditions.

Key words: Endometrium; whole slide imaging; endometrial cancer; endometrial hyperplasia; endometrial carcinoma; digital slide repository; image analysis; image segmentation; histopathology; deep learning; machine learning.

Data Description

The endometrial dataset described in this paper, includes a total of 2909 H&E stained WSIs from NHS Greater Glasgow and

Compiled on: February 14, 2025.
Draft manuscript prepared by the author.

Clyde Biorepository and Pathology with a total of 3.6 TB storage. This dataset was originally created as part of Industrial Centre for iCAIRD [1] with the aim to automatically sort histopathology whole slide images of endometrial biopsies into one of three categories, "malignant", "other or benign" or "insufficient". This would allow prioritisation of malignant slides within the pathologists' workload and reduce the time to diagnosis for patients with cancer.

Context

As the demand for Artificial Intelligence (AI) services continues to grow, so does the need for high-quality datasets. Data is the key component of any Machine Learning (ML) and deep learning projects. The quality of data is as important as the quantity and hence data preparation and understanding is one of the most important and time-consuming tasks of the Machine Learning project life cycle.

Machine learning in healthcare can be used for better diagnosis using ML-enabled tools to analyse medical reports and images. The use of AI in clinical practice aid pathologists in many ways. Techniques like digital image analysis and machine learning are excellent in predicting cancer outcomes. These AI models can help with pathological diagnosis and train pathologists to identify areas of interest in tissue samples.

Endometrial cancer is a type of cancer that originates in the lining of the uterus, which is called the endometrium. It is one of the most common forms of cancer that affects the female reproductive system. The endometrium is the tissue that undergoes changes throughout the menstrual cycle and is shed during menstruation.

Using AI for the detection of endometrial cancer has shown promising results in recent research and clinical applications. AI techniques, such as machine learning and deep learning, can be applied to medical imaging and clinical data to aid in early detection and accurate diagnosis of endometrial cancer.

A recent review of artificial intelligence in gynecological cancers [2] found 13 papers for endometrial cancer, out of which only one paper used H&E WSIs from endometrial biopsies [3]. In this paper a CNN was trained on patches of size 640×640 pixels extracted from the regions annotated by pathologists as normal or malignant. Convolutional Neural Network (CNN) differentiated patches as endometrial adenocarcinoma and 3 benign classes, normal, endometrial polyp, and endometrial hyperplasia and achieved 93.5% accuracy on the binary classification task and 78.0% sensitivity. The results presented in this paper are at the patch level only and no slide level classification has been reported.

A endometrial cancer H&E slides dataset, CPTAC [4] is available on cancer imaging archive, consisting of pathology slides along with genomics data and radiology images. The three studies that used CPTAC aimed to predict the same information as genetic sequencing [5] or illustrate features in H&E slides that could identify different cancer variants [6, 7] and hence allow more personalised treatment.

A weakly supervised learning method used this endometrial dataset for whole slide image diagnosis and interpretability. Interpretability methods including attention heatmapping, feature visualisation, and a novel end-to-end saliency-mapping, were applied to identify distinct morphologies learned by the model and build an understanding of its behaviour [8]. The reported results in this article shows slide level validation and test accuracies over 85% and 87% respectively. This dataset also has been used for detection of malignancy using AI in a recent article [9]. In this article, a fully supervised CNN model was trained to automatically sort endometrial biopsy images into "malignant", "other or benign" or "insufficient" tissue classes with the aim to allow prioritisation of these slides in a queue for pathologist review and hence reduce time to diagnosis for patients with cancer. The final model was able to accurately classify 90% of all slides correctly and 97% of slides in the malignant class; this accuracy is good enough to allow prioritisation of the workload. The code and trained model for this paper is available at [10].

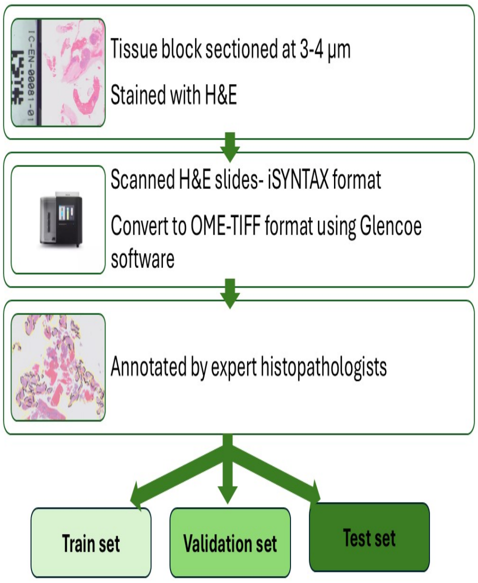

Figure 1. Flow chart of Data Collection and Data Split

Methods

Data collection

The tissue blocks for this study originate from Glasgow Royal Infirmary (NG), Southern General Hospital (SG), Royal Alexandria Hospital (RAH) and Queen Elizabeth University Hospital (QEUH) (all in Glasgow, Scotland) each with independent tissue handling including fixation and tissue processing. New tissue sections were cut from the tissue blocks at one of two different thicknesses (3 or 4 microns) and then stained with one of four different H&E protocols. Together, these combinations gave eight different labs maximising WSI variance and thereby decreasing the likelihood of overfitting to any one lab (combination of tissue processing, cutting and staining protocol) ((Figure 1).

Data split to train and test sets

The slides were split into training, validation, and test sets. The samples had examples of five "malignant" subcategories, five "other or benign" subcategories, and a category "insufficient", where there was insufficient tissue to make a diagnosis. Hyperplasia with atypia was included in the "malignant" category as it is a high risk pre-invasive lesion which it is important to detect (Figure 2).

The test set contained the complete groups of slides for two of the labs and these slides were not part of the training and validation sets. The test set then also contained a randomly selected 10% of the slides from the other 6 labs. The remaining 90% of the slides, from the other 6 labs were used for the training and validation sets. Two thirds of these slides were selected randomly for the training set and the rest were used for the validation set. The splits into the test, validation, and training sets were checked to see that there was a balance of the categories and subcategories across the sets. These splits were calculated based on the case labels associated with the samples recorded in the system. During the annotation process these labels were doubled checked and in approximately 5% of the cases the final label associated with the scanned slide was different. This could be because the new slice taken from the sample did not show the same pathology as the original or that the original label

Table 1. Distribution of samples in training, validation, and test sets for endometrial dataset.

Category	SubCategory	Training	Validation	Test	Total
Malignant	- Adenocarcinoma	243	113	162	518
	- Carcinosarcoma	37	18	28	83
	- Sarcoma	11	6	8	25
	- Hyperplasia with atypia	106	53	67	226
	- Other	4	1	3	8
	Total	401	191	268	860
Other or benign	- Hormonal	158	79	115	352
	- Inactive atrophic	170	90	133	393
	- Proliferative	184	81	116	381
	- Secretory	176	91	116	383
	- Menstrual	159	84	111	354
	Total	847	425	595	1867
Insufficient	- Insufficient	90	44	48	182

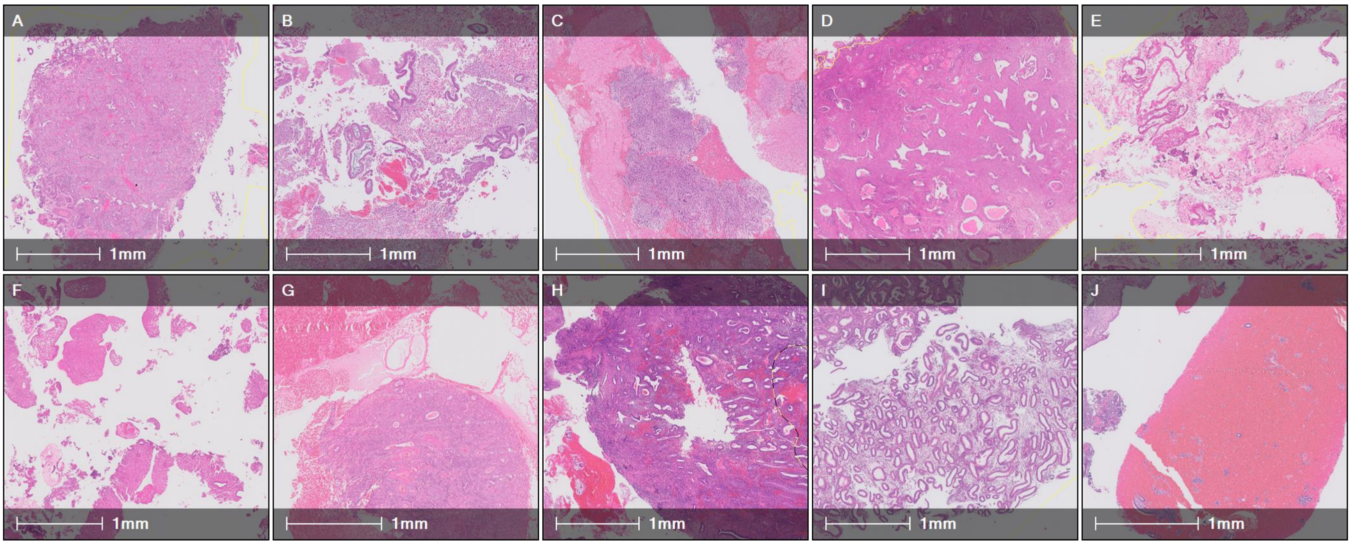

Figure 2. Example of subcategory of malignant and other or benign cases
A. Malignant-Adenocarcinoma, B. Malignant-Carcinosarcoma, C. Malignant-Sarcoma, D. Malignant-Hyperplasia with atypia, E. Malignant-Other, F. Other or Benign-Hormonal, G. Other or Benign-Inactive atrophic, H. Other or Benign-Proliferative, I. Other or Benign-Secretory, J. Other or Benign-Menstrual

was incorrectly recorded. The corrected labels post annotation were the labels that were used for training and testing. This means the final numbers of slides of each type may not match the original percentages described above. The distribution of data over train, validation and test sets is shown in table 1.

All slides were then scanned at QEUH and saved as Whole Slide Images (WSIs). The WSIs are hundreds of thousands of pixels in height and width at the highest magnification and are too large to read into memory. Dedicated WSI formats allow access to either small parts of the image at the highest magnification or the whole image at lower magnifications. For this study, slides were scanned using a Phillips Ultra Fast Scanner (UFS) and stored in the iSyntax file format. The most detailed view in the WSI is level 0, or 40x magnification where the length of a side of 1 pixel in the image is 0.25μm. Higher levels represent lower magnifications in a pyramid where each level is a power of 2 smaller than the previous (Figure 1).

Annotation process

The scanned slides were annotated by a mix of experienced biomedical scientists and pathologists from NHS Greater Glasgow and Clyde. The work of the biomedical scientists was reviewed and approved by a pathologist before use. The annotations took place using the QuPath software [11] the isyntax [12] files were converted to OME-Tiff files using a Glencoe software converter [13] prior to annotation.

Annotation endometrial slides is complicated due the structure of the tissue present on the slides. Some of the slides contained a small number of large contiguous pieces of tissue (Fig 3a), where only annotating the malignant areas is straight forward. However, some of the slides contained a very large number of small fragments of tissue (Fig 3b). These slides would require the pathologists to annotate separately many small bits of tissue on slides where nearly all the tissue was malignant. In addition, some slides contained a very large amount of blood or mucus with no diagnostic value (Fig 3c). Therefore, it was decided that annotating blood and mucus either as a separate class or as part of the "other or benign" class would be time prohibitive and an alternative approach was needed. The widely used method for annotating H&E slides takes the approach that only the area of interest is annotated and the rest of tissue is considered as normal tissue and therefore is not annotated. Due to the structures complexity of the endometrial slides mentioned above, it was decided to take a different approach for annotating these slides.

The annotation approach taken for endometrial WSIs gives an overall class to the slide, and then to only annotate parts of the slide that differed from the overall class. The classes used for annotation were "malignant" and "other or benign". Although there are slides categorised as "insufficient", these slides are characterised by a lack of tissue rather than a specific type of tissue, so "insufficient" was not used as an annotation class. Annotators were not required to denote the areas of tissue on the slide as tissue detection was applied as part of the pre-processing algorithm. Hence, a large number of the annotation files were blank as everything on the

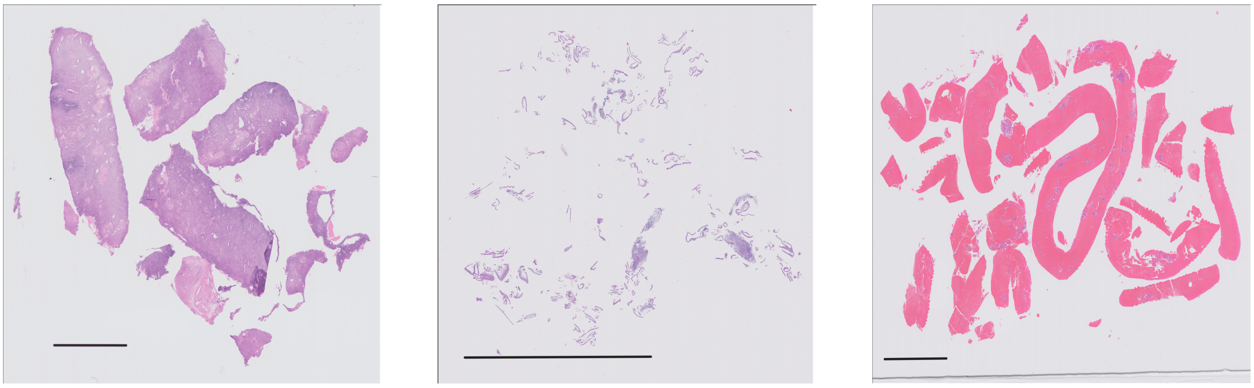

(a) Slide with a few large pieces of tissue. **(b)** Slide with many small fragments of tissue. **(c)** Slide with large amounts of blood clot.

Figure 3. Examples of slides with different amounts and presentation of tissue (Scale bar = 5mm).

slide was from the overall class with no other annotation required.

Fig 4 shows examples of endometrial slides where all the tissue on the slide is of the overall category assigned to that slide and therefore the annotation files for them are blank as no annotation was needed for them. Tissue detection or background separation and blood and mucus detection are then applied to the slide in preprocessing stage. To detect the tissue and separate it from the background, a thumbnail image of the slide at level 5 is created. Fig 3c shows how multiple tissue areas are saved as separate images in iSyntax format to reduce the file size. In the thumbnails the missing areas between these images are pure black pixels. Any pixels in the thumbnail that are pure black are converted to pure white. The image was then converted to greyscale and as the background is predominately white any values of greater than 0.85 were considered to be background. Next a closing transform and a hole filling morphological operation are applied, the operations improve the amount of tissue captured around edges and holes. The mask created by the tissue detection algorithm for the slide shown in Fig 4a is shown in Fig 4c when when tissue detection is combined with the annotation (Fig 4b) it gives the areas of the slide as "malignant" or "other or benign" tissue as shown in Fig 4d.

The second stage is to identify any blood or mucus on the slide. Blood and mucus detection is carried out on a pixel by pixel basis. Each of the red, green and blue (RGB) channels are considered separately. A Gaussian filter with a kernel size of 2 is applied. Then a texture filter is applied to each channel both with and without the Gaussian filter to give a total of 12 different features for each pixel (raw pixel value, Gaussian filtered value, texture filter on raw, texture filter on Gaussian filter, for each of 3 channels). A random forest model was trained using a small subset of images with detailed annotations to determine the difference between "blood or mucus" and "tissue" pixels. The trained blood and mucus detection model was then applied to each image to identify "blood or mucus". For the slide shown in Fig 5A the areas detected as "blood or mucus" are shown in Fig 5E when this is combined with the tissue detection and annotations it gives the areas of the slide as "malignant" or "other or benign" as shown in Fig 5.

Fig 6 shows examples of slides where different categories are present on the slide. In these examples the background and blood and mucus area are detected later in tissue detection and blood or mucus detection stages.

Inter-observer variability in annotation

Disagreements can occur among pathologists when categorizing slides. To assess this variability, three pathologists independently annotated a subset of 295 test slides. Their agreement was measured using Cohen's kappa statistic, with the arithmetic mean of all observer pairs reported [9]. Despite strong concordance, some inconsistencies were observed. The most frequent category-level disagreements were between "insufficient" and "other benign", while at the subcategory level, differences arose mainly between "insufficient" and "inactive/atrophic", as well as "hyperplasia with atypia" and "adenocarcinoma" within the malignant class. These disagreements are visualized in the confusion matrices [9].

Data Validation and quality control

The images submitted were obtained directly from cases undergoing clinical histopathological diagnosis and were subject to rigorous scrutiny by the specialist team of diagnostic histopathologists who undertook the manual annotations of selected features. The annotations were added afterwards as a separate exercise, not linked to clinical diagnosis. The gold standard was the pathologists' diagnosis and where there was discrepancies, by consensus review.

Using H&E endometrial WSI dataset and their annotations, ML algorithms can be applied to assist in various aspects of cervical health analysis. Data collection and preprocessing is the first step in illustrating how ML algorithms can utilise this data.

Extracting nuclear morphological features using Indica Halo AI

WSI images were imported into Indica HALO and HALO AI (v.3.6.4134), along with corresponding annotation files created in QuPath by pathologists. A nuclei segmentation classifier, underpinned by advanced deep learning neural network algorithms, was trained with examples from multiple different cases 7. An analysis algorithm, Multiplex IHC v.3.2.3 was utilised to segment individual nuclei to extract nuclear morphological features such as area, perimeter, and roundness within the annotation (Figure 5). The tabular data from the individual nuclear morphological features, along with their x and y coordinates, was exported into CSV file format.

Re-use potential

The endometrium can display a wide range of histological appearances with overlapping features which makes the diagnosis of various lesions complex and specifically distinguishing between premalignant and malignant conditions challenging. The diverse presentation of symptoms of endometrial abnormalities may be attributed to different underlying conditions, making accurate diagnosis based solely on clinical presentation impossible. This dataset includes a wide range of endometrial whole slide images containing a wide spectrum of histological conditions that have been annotated by pathologists and can be used for training AI based algorithms to identify the endometrial abnormalities and to detect slides with malignant tissue to allow prioritisation of these slides in a queue for pathologist review and hence reduce time to diagnosis for patients

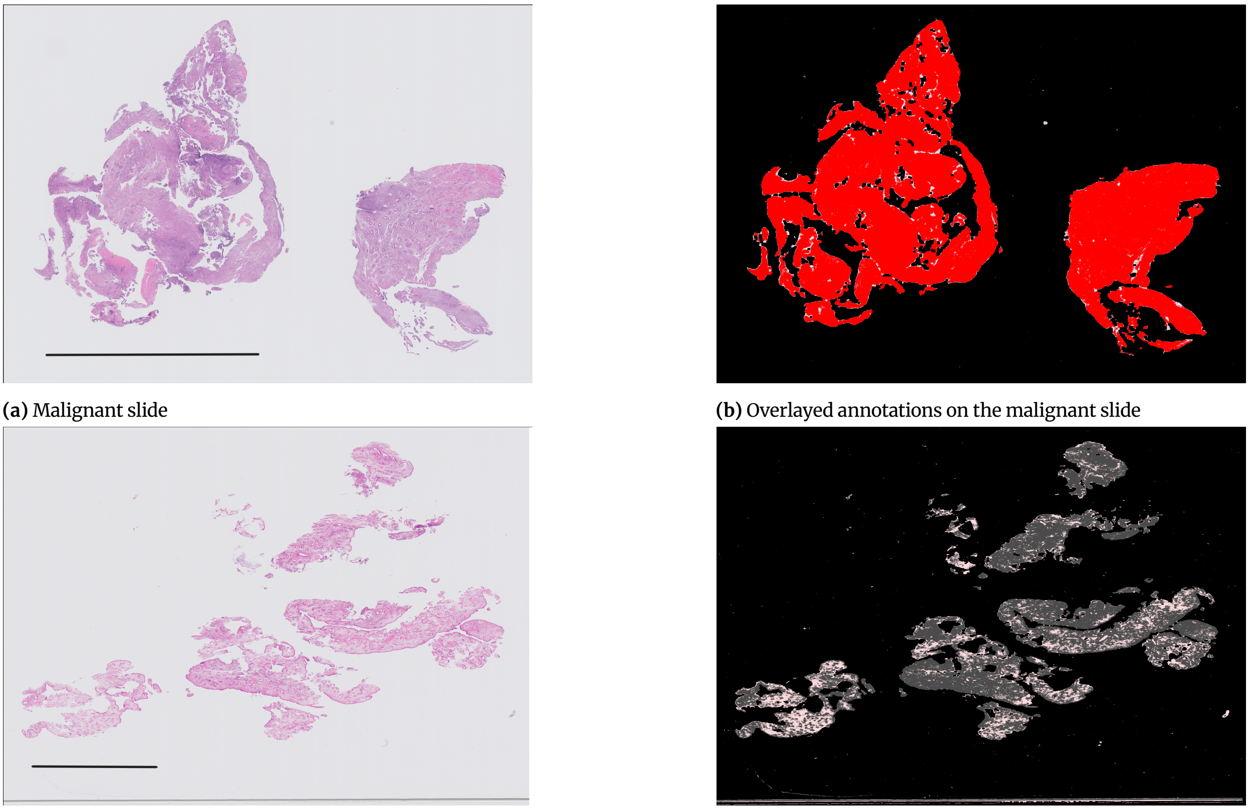

(a) Malignant slide

(b) Overlayed annotations on the malignant slide

(c) Normal slide

(d) Overlayed annotations on the normal slide

Figure 4. Examples of slides where all tissue on the slide is of the same category. (Scale bar = 5mm)

Malignant blood or mucus Normal Tissue Background

with cancer. Moreover, nuclear morphological features such as area, perimeter, and roundness may enhance accuracy in distinguishing between pre-malignant and malignant. Furthermore, one problem in diagnostic pathology is how to deal with small samples that may not be representative nor provide conclusive evidence for the pathologist. On occasion this may necessitate a report that states "tissue insufficient for diagnosis", which in turn may lead to a further sample being sought. This is a potential distressing and painful procedure for the patient. Being more confident about defining an inadequate sample will help workflow, ensure safety and minimise unnecessary discomfort to the patient.

Ethical Approval

- Ethics approval for the study was granted by NHS Greater Glasgow and Clyde Biorepository and Pathology Tissue Resource (REC reference 16/WS/0207) on 4th April 2019.
- Biorepository approval was obtained (application number 511)
- Local approval was obtained from the School of Computer Science Ethics Committee, acting on behalf of the University Teaching and Research Ethics Committee (UTREC) [Approval code-CS15840].

Data availability

All endometrial whole slide images, their annotation files, binary masks and a metadata file (2909 images in iSyntax format, 2909 annotation files in JSON format, 2909 binary masks in PNG format and a metadata file in CSV format) and the morphological features extracted from them in Halo are openly available in the GigaScience repository, GigaDB [S-BIAD1199] [14].

Declarations

List of abbreviations

- Artificial Intelligence (AI)
- Machine Learning (ML)
- Whole Slide Image (WSI)
- Convolutional Neural Network (CNN)
- Clinical Proteomic Tumor Analysis Consortium (CPTAC)
- Teta Bytes (TB)
- Industrial Centre for Artificial Intelligence Research in Digital Diagnostics (iCAIRD)
- Quantitative Pathology (QuPath)

Consent for publication

Not applicable.

Competing Interests

The authors declare that they have no competing interests.

Funding

This work is supported by the Industrial Centre for AI Research in digital Diagnostics (iCAIRD) which is funded by Innovate UK on behalf of UK Research and Innovation (UKRI) [project number: 104690], and in part by Chief Scientist Office, Scotland.

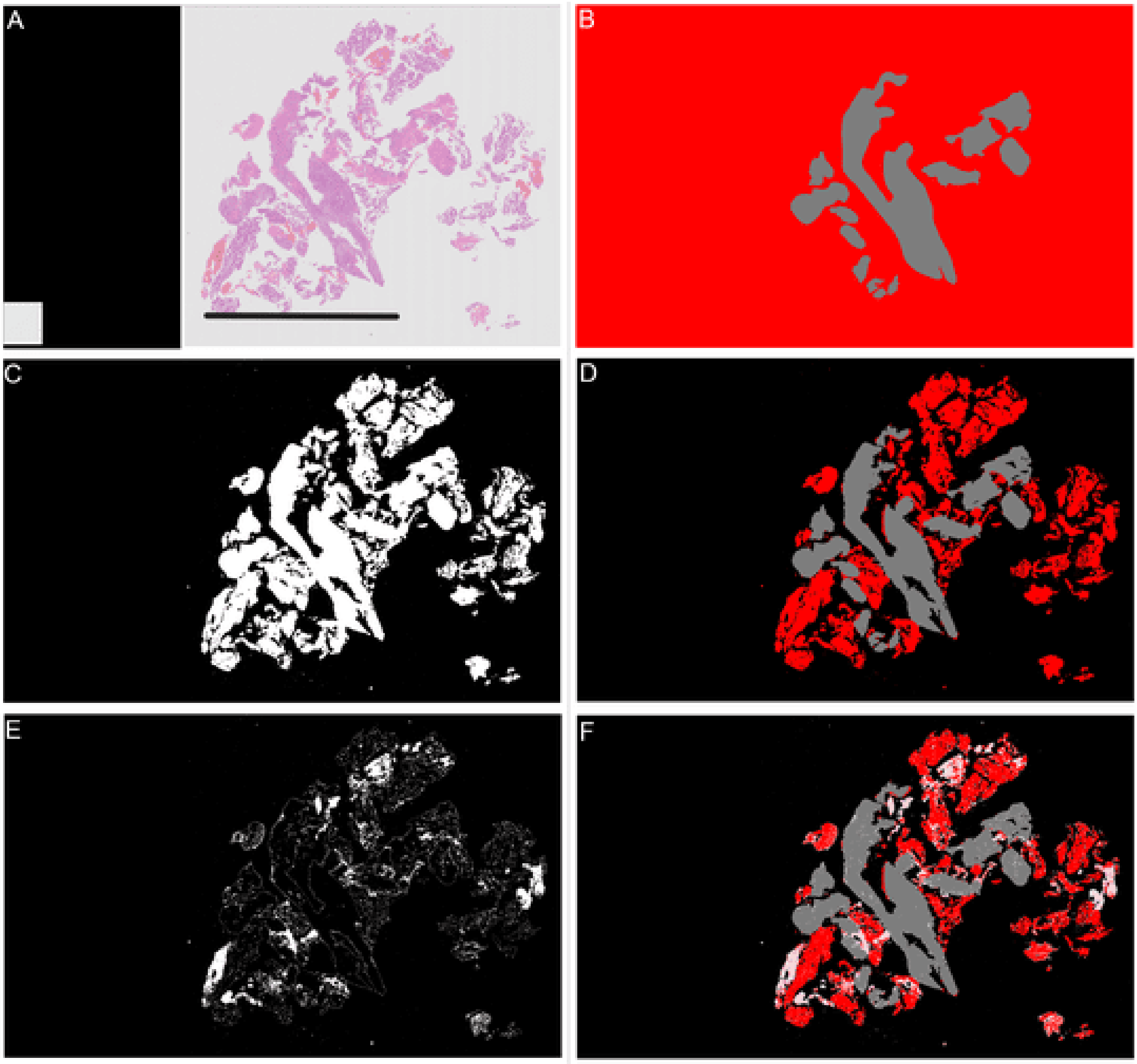

Figure 5. Examples of all stages in slide annotation and detection of tissue. (Scale bar = 5mm)
(A) Thumbnail of "malignant" slide where some tissue is "other or benign". (B) Annotation for "malignant" slide where some tissue is "other or benign". (C) Mask showing areas detected as tissue in white, background is shown in black. (D) Combined annotation and tissue detection. (E) Calculated mask showing areas detected as "blood or mucus" in white, anything that is not "blood or mucus" is shown as black. (F) Combined annotation, tissue and "blood or mucus" detection.
Malignant blood or mucus Normal Tissue Background

Author's Contributions

Mahnaz Mohammadi wrote the manuscript and supervised data preprocessing, together with Christina Fell and In Hwa Um.
Prishma Shahi imported annotations in Indica Halo AI platform and measured nuclear morphological features.
Gareth Bryson initiated the project, and Sarah Bell, Sheeba Syed, and Prakash Konanahalli annotated the whole slide images. David Harris Birtill and Ognjen Arandjelovic supervised machine learning experiments.
Clare Orange arranged data release from Glasgow Biorepository. James Blackwood oversaw governance procedures, established digital pathology services and supervised data de-identification and release. David Harrison is Director of iCAIRD, obtained funding, reviewed results and helped to draft the manuscript. All authors have seen and approved the manuscript.

Acknowledgements

We acknowledge the support of NHS Research Scotland (NRS) Greater Glasgow and Clyde Biorepository. We acknowledge the support of the biomedical scientists, Tim Prosser, Lucy Irving, Jennifer Campbell and Jennifer Faulkner, from the Pathology Department, NHS Greater Glasgow and Clyde for technical support.

Authors' information

MM and CF hold a PhD degree and are data scientists in the School of Medicine, University of St Andrews.
PS is a research technician at the School of Medicine, University of St Andrews.
GB is a Consultant Pathologist and Clinical Director for Laboratory

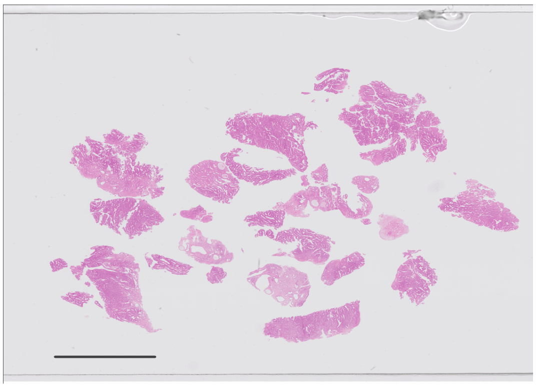

(a) Malignant slide

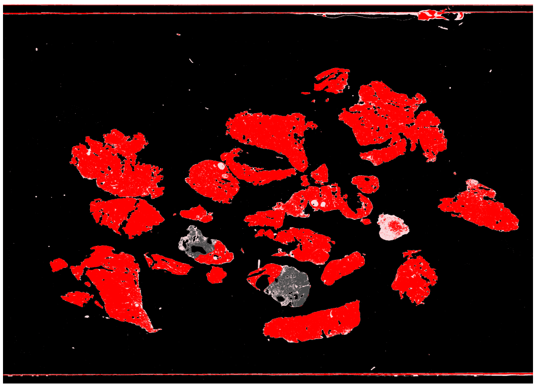

(b) Mask of the malignant slide

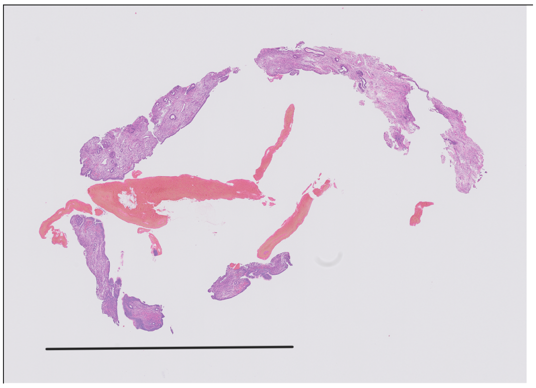

(c) Normal slide

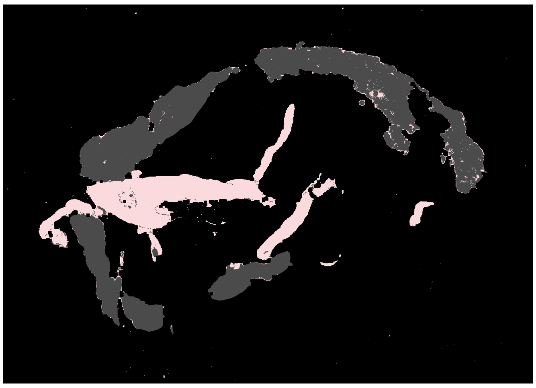

(d) Mask of the normal slide

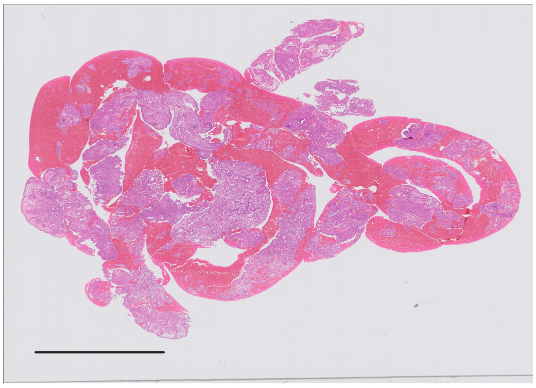

(e) Malignant slide

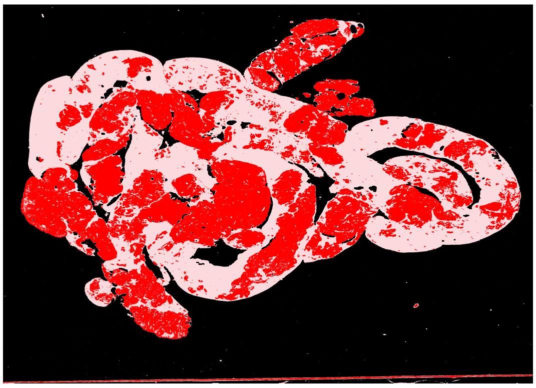

(f) Mask of the malignant slide

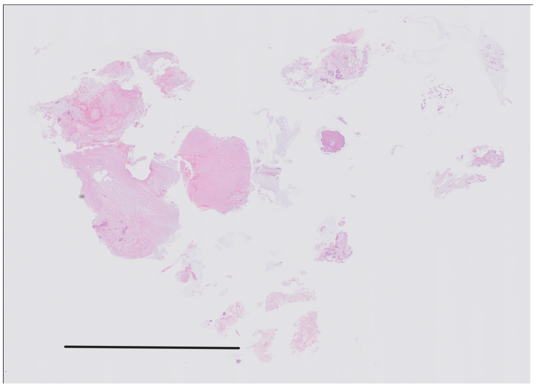

(g) Insufficient slide

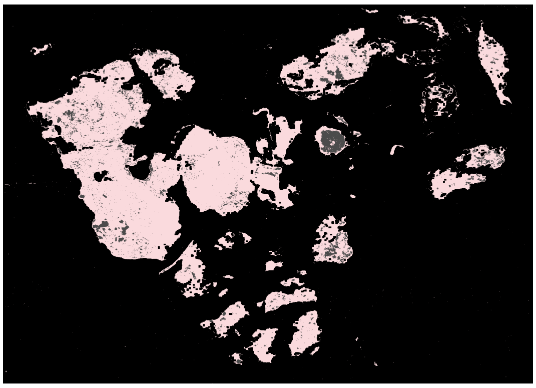

(h) Mask of the insufficient slide

Figure 6. Examples of slides and their masks after applying overlaying annotations and applying tissue and blood or mucus detection stages to the slide. (Scale bar = 5mm)

■ Malignant □ blood or mucus ■ Normal Tissue ■ Background

Medicine at the Queen Elizabeth University Hospital, Glasgow. SB, PK and SS are consultant gynaecological pathologists at Queen Elizabeth University Hospital, NHS Greater Glasgow and Clyde, UK. OA and DHB are Reader and Senior Lecturer respectively in Computer Science, University of St Andrews.
IHU is a postdoctoral research fellow in pathology AI in the Univer-

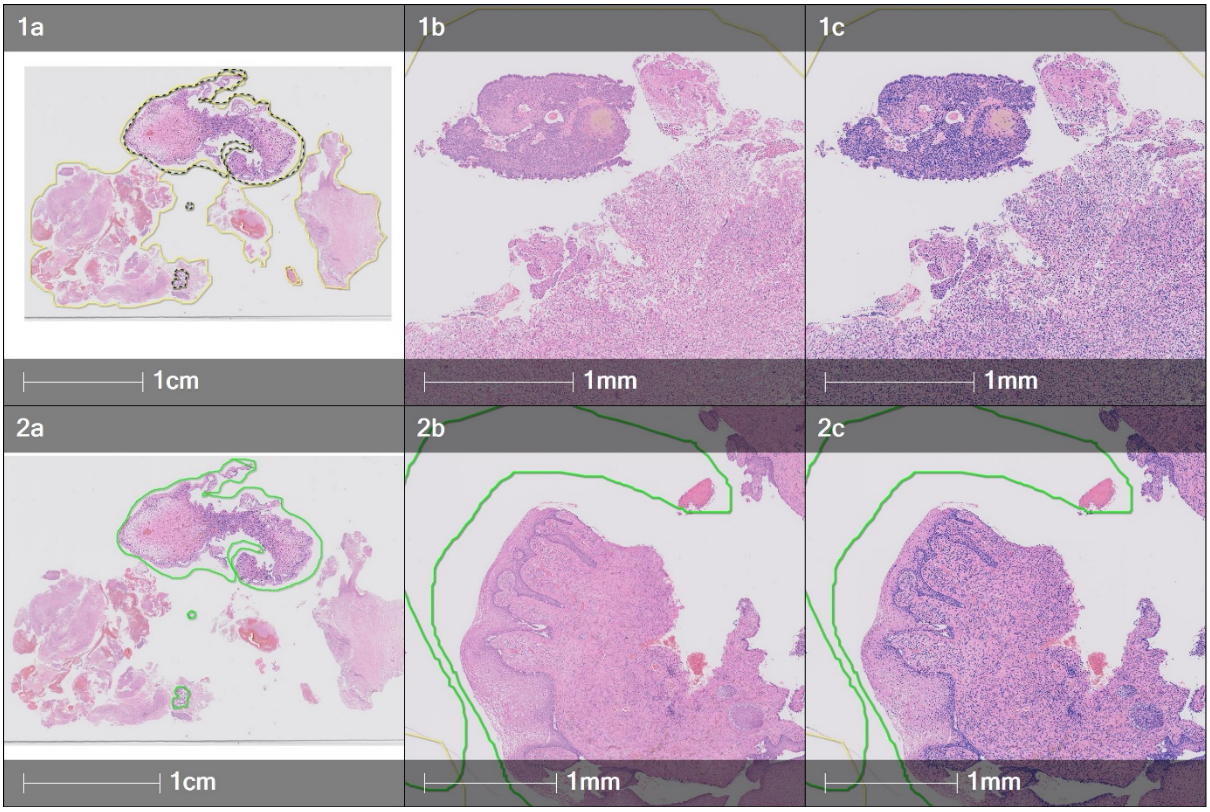

Figure 7. Example of segmented nuclei (colored blue) in two different annotations in the same patient using Indica HALO AI platform
(1a) Annotation of the malignant area (yellow line), having excluded the normal cervix (dotted yellow annotation). (2a) Annotation of normal cervix (green line). (1b, 2b) Higher magnification of 1a and 2a, respectively. (1c, 2c) Multiplex IHC analysis algorithm was used to segment individual nuclei (Blue nuclei mask) and to extract their morphological features within annotations.

sity of St Andrews. CO is Biorepository Manager in NHS Greater Glasgow and Clyde, and a doctoral candidate in the University of St Andrews.
JDB is Chief Technical Officer of iCAIRD and Innovation Fellow in University of St Andrews. DJH is Professor of Pathology at the University of St Andrews, and Director of iCAIRD.

References

1. Gynaecological Cancer AI.;. https://icaird.com/wp9-gynaecological-cancers/.
2. Akazawa M, Hashimoto K. Artificial intelligence in gynecologic cancers: Current status and future challenges–A systematic review. Artificial Intelligence in Medicine 2021;120:102164.
3. Sun H, Zeng X, Xu T, Peng G, Ma Y. Computer-aided diagnosis in histopathological images of the endometrium using a convolutional neural network and attention mechanisms. IEEE journal of biomedical and health informatics 2019;24(6):1664–1676.
4. Consortium NCICPTA, et al. Radiology data from the clinical proteomic tumor analysis consortium lung squamous cell carcinoma [cptac-lscc] collection [data set]. Cancer Imaging Archive 2018;.
5. Hong R, Liu W, DeLair D, Razavian N, Fenyö D. Predicting endometrial cancer subtypes and molecular features from histopathology images using multi-resolution deep learning models. Cell Reports Medicine 2021;2(9).
6. Wang T, Lu W, Yang F, Liu L, Dong Z, Tang W, et al. Microsatellite instability prediction of uterine corpus endometrial carcinoma based on H&E histology whole-slide imaging. In: 2020 IEEE 17th international symposium on biomedical imaging (ISBI) IEEE; 2020. p. 1289–1292.
7. Fremond S, Andani S, Koelzer VH, et al. Interpretable deep learning predicts the molecular endometrial cancer classification from H&E images: a combined analysis of the PORTEC randomized clinical trials. SSRN 2022;(4144537).
8. Mohammadi M, Cooper J, Arandelović O, Fell C, Morrison D, Syed S, et al. Weakly supervised learning and interpretability for endometrial whole slide image diagnosis. Experimental Biology and Medicine 2022;247(22):2025–2037.
9. Fell C, Mohammadi M, Morrison D, Arandjelović O, Syed S, Konanahalli P, et al. Detection of malignancy in whole slide images of endometrial cancer biopsies using artificial intelligence. Plos one 2023;18(3):e0282577.
10. Fell C, Mohammadi M, Morrison D. StAndrewsMedTech/icairdpath-public: Release for publication 2023 Feburary;https://zenodo.org/record/7674764.
11. Bankhead P, Loughrey MB, Fernández JA, Dombrowski Y, McArt DG, Dunne PD, et al. QuPath: Open source software for digital pathology image analysis. Scientific reports 2017;7(1):1–7.
12. Hulsken DB, iSyntax – your format for pathology images; 2016. https://www.openpathology.philips.com/isyntax.
13. Mellisa Linkert, Chris Allan, Converting Whole Slide Images to OME-TIFF: A New Workflow; 2019. https://www.glencoesoftware.com/blog/2019/12/09/converting-whole-slide-images-to-OME-TIFF.html, Last accessed on 2022-08-12.
14. In Hwa Um CFDMSBGBSSPKCOPSDHDHB Mahnaz Mohammadi, Arandelovic O, Endometrial Whole Slide Images Dataset; 2024. https://www.ebi.ac.uk/biostudies/bioimages/studies/S-BIAD1199.

Click here to access/download;Figure;Fig1.png

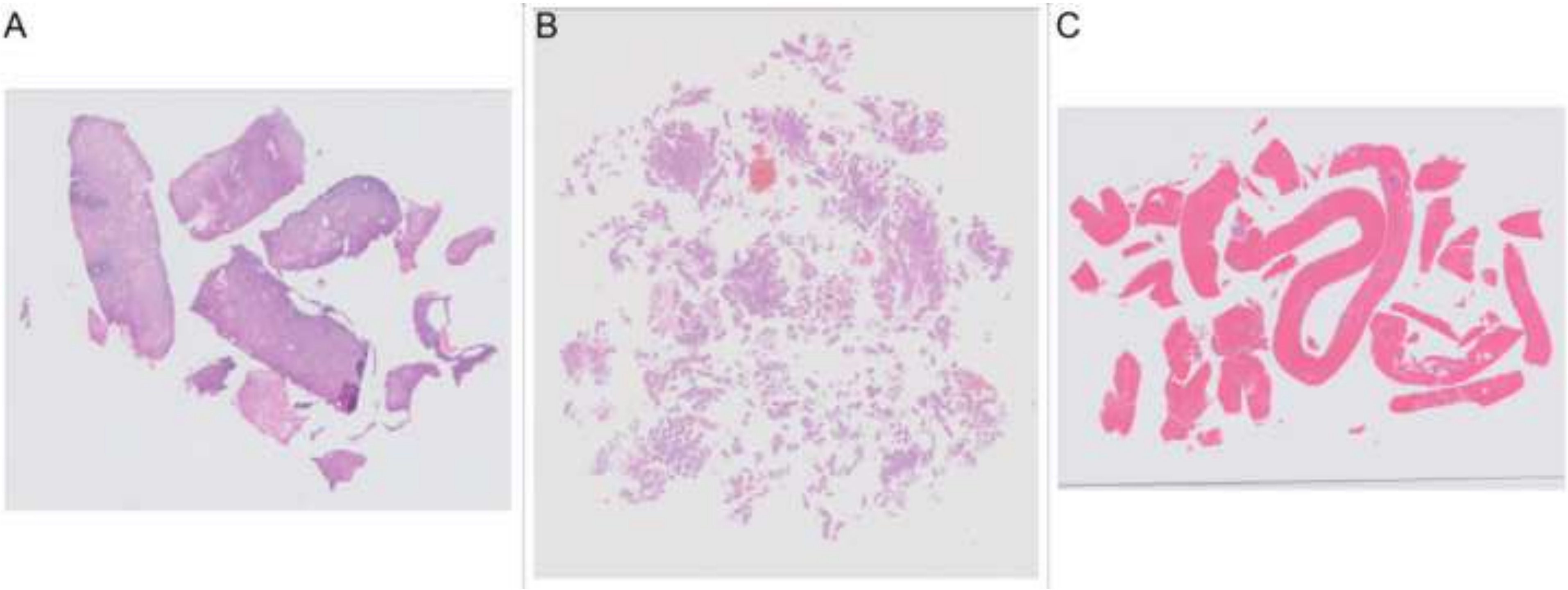

Click here to access/download;Figure;halo_endo_features.jpg

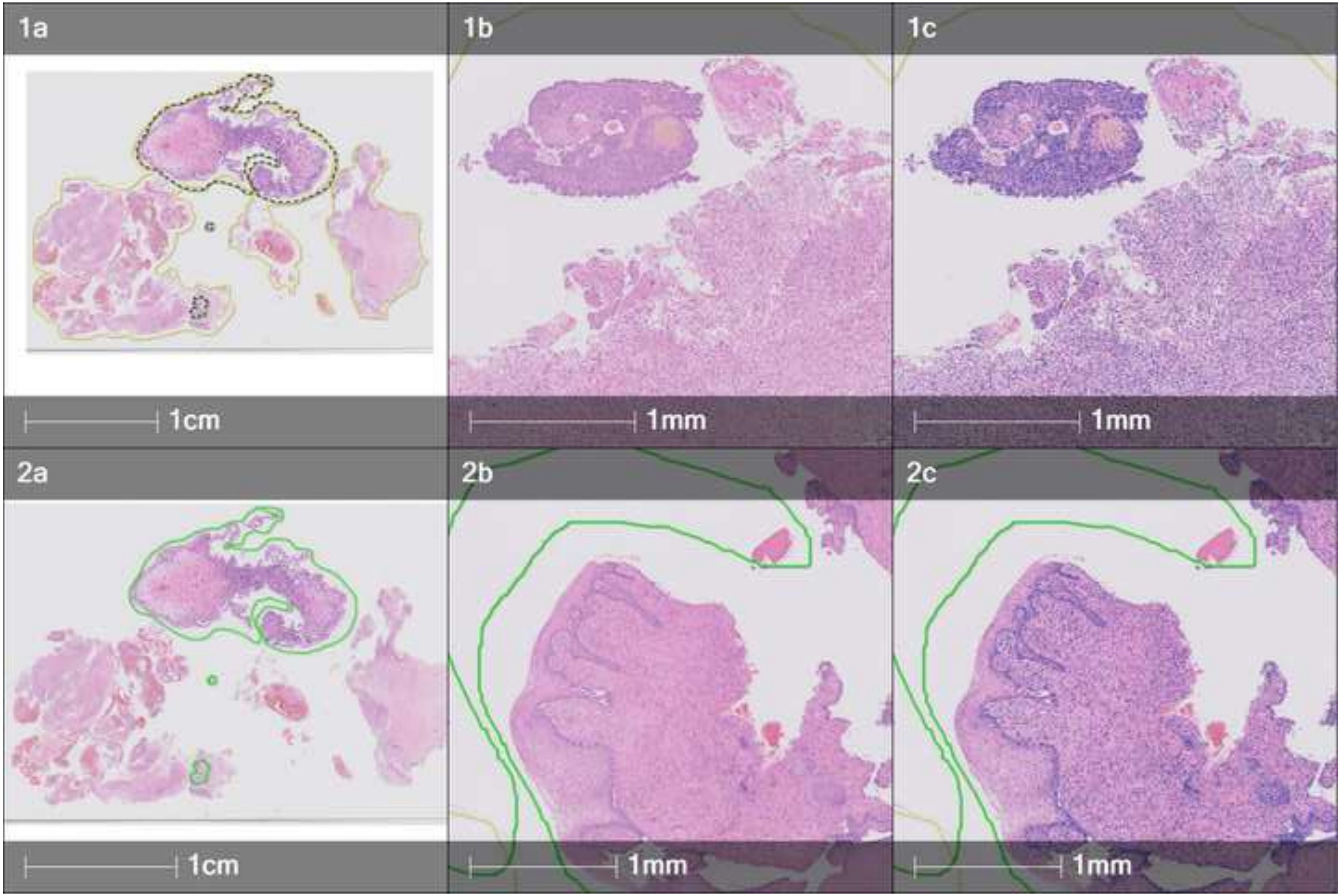

Click here to access/download;Figure;IC-EN-00020-01_mask.png

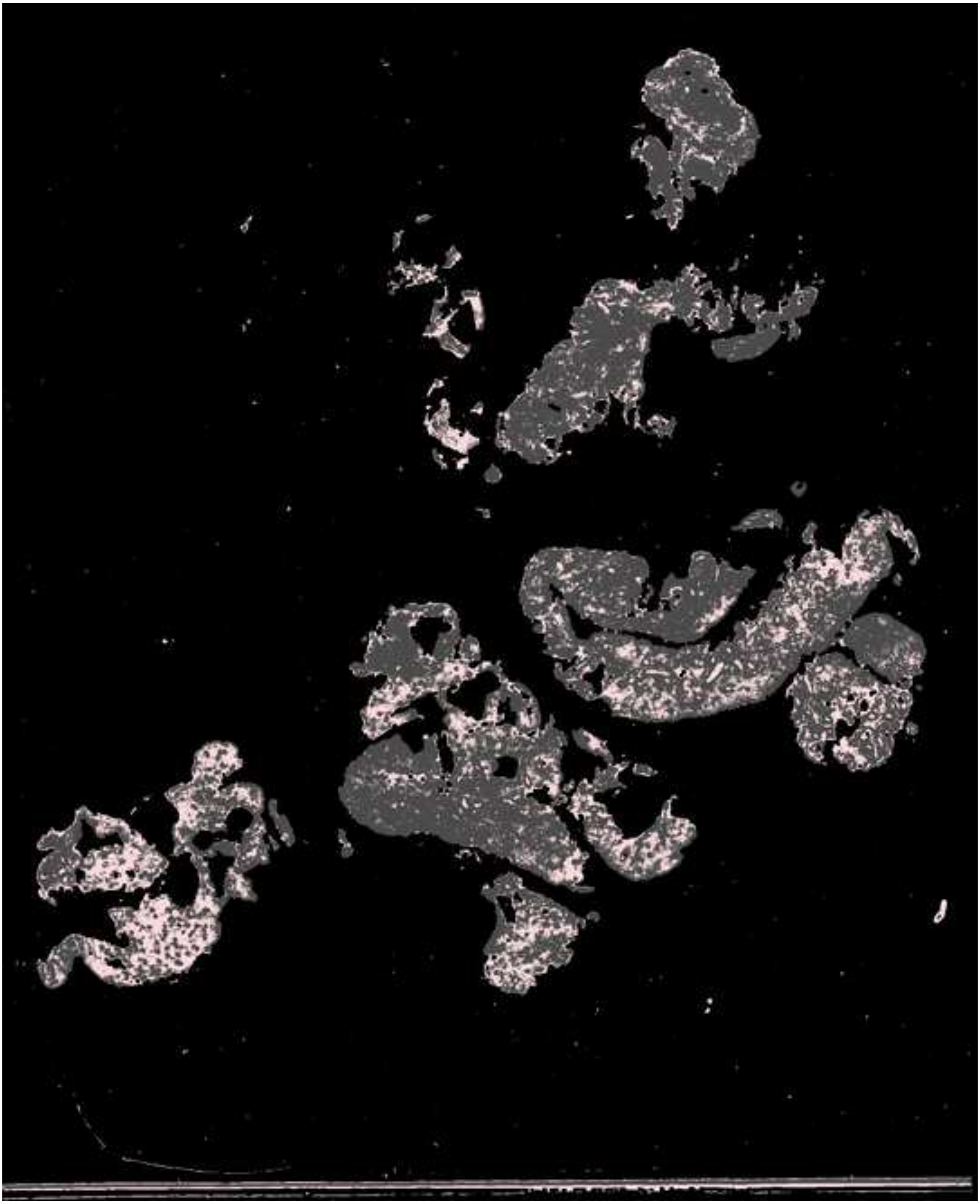

Click here to access/download;Figure;IC-EN-00027-01_mask.png

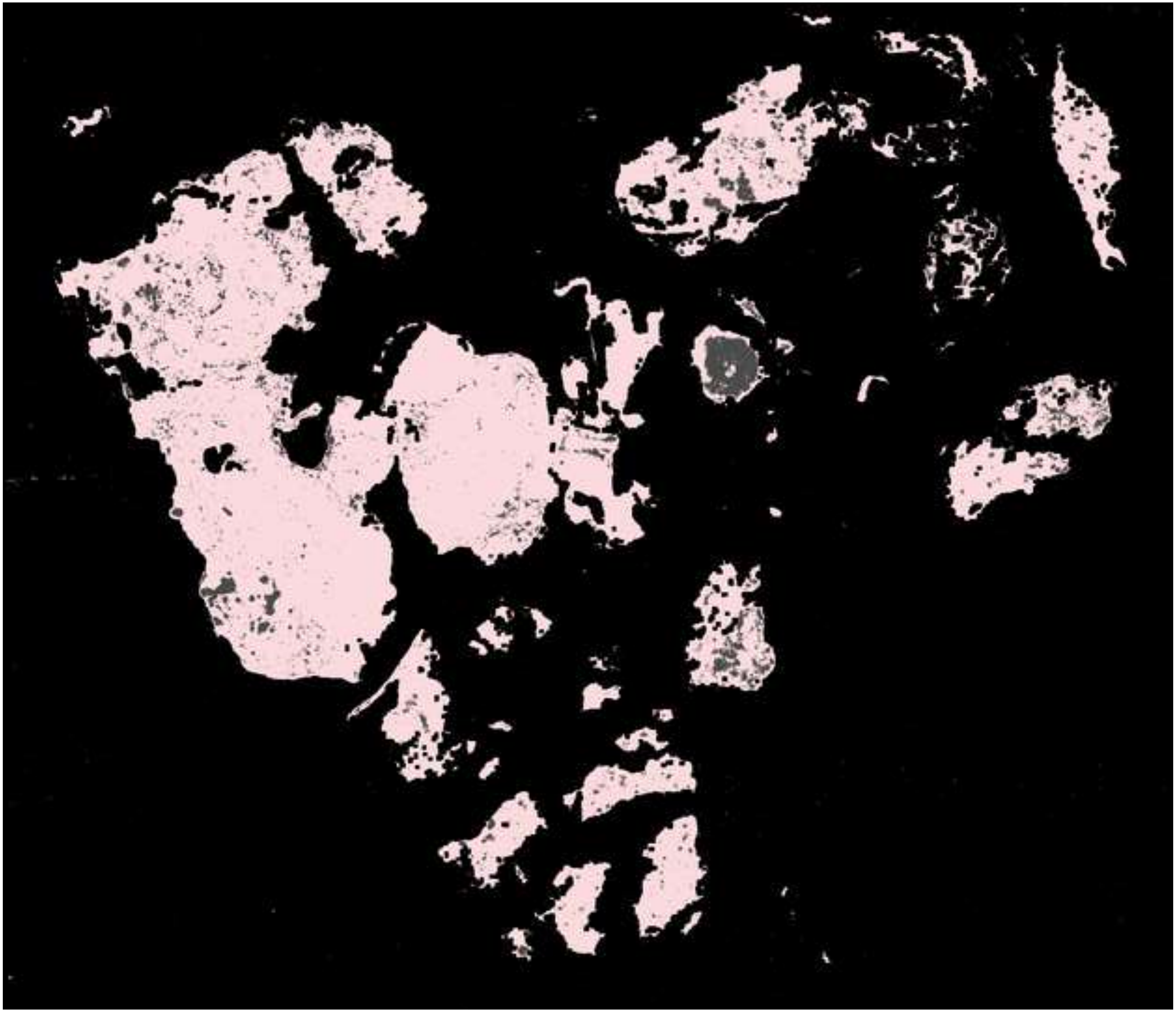

Click here to access/download;Figure;IC-EN-00029-01_mask.png

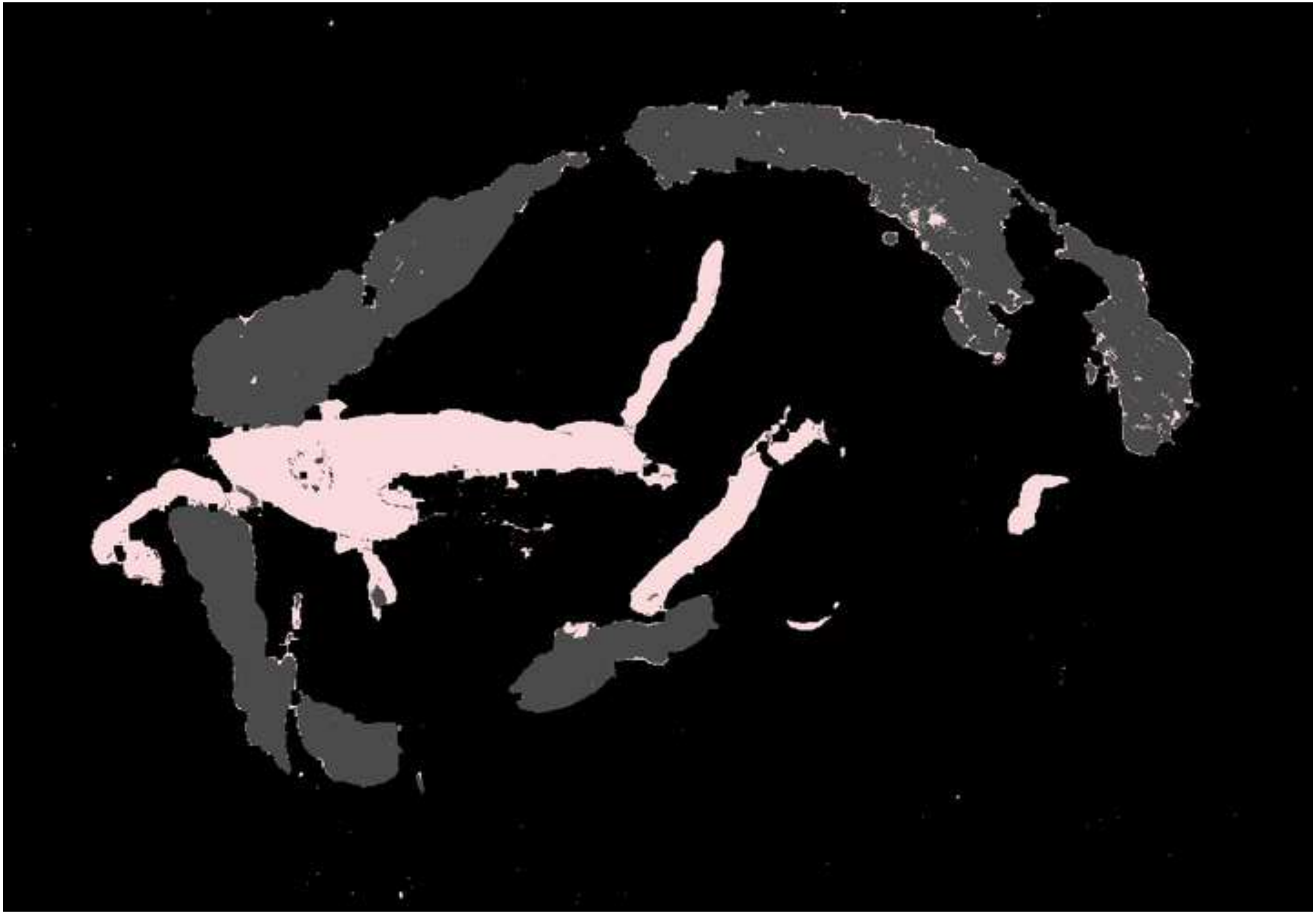

Click here to access/download;Figure;IC-EN-00033-01_mask.png

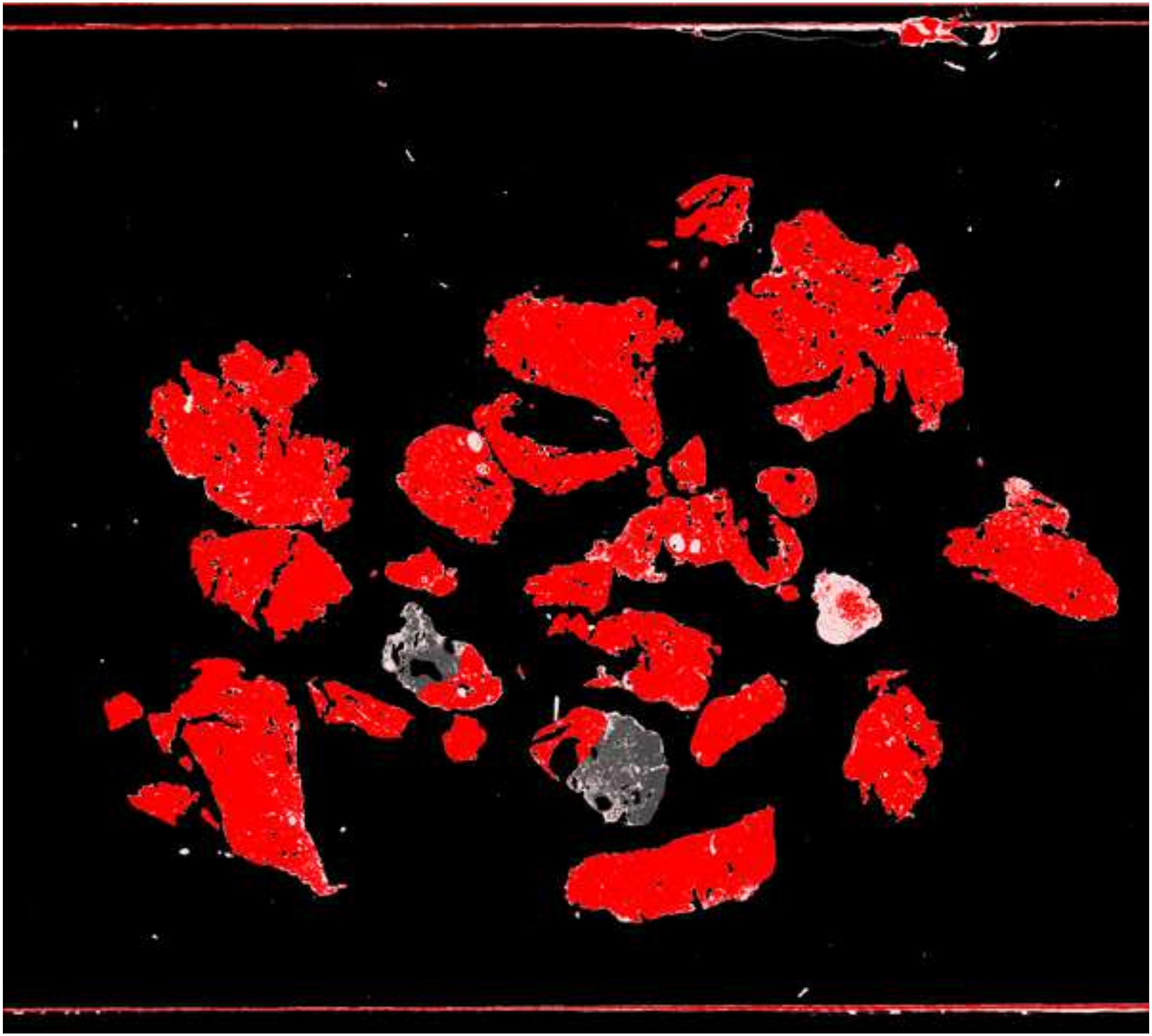

Click here to access/download;Figure;IC-EN-00040-01_mask.png

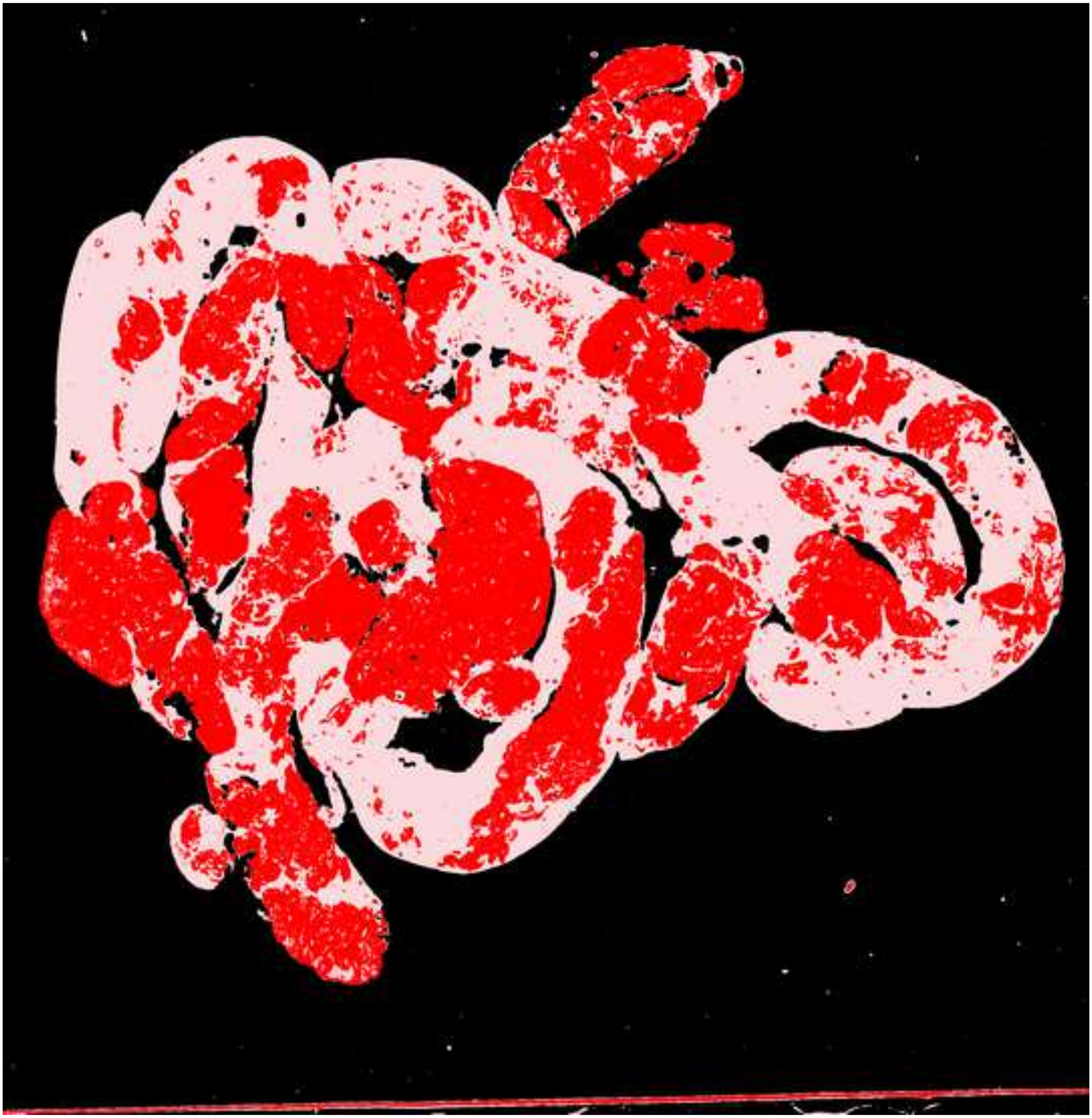

Click here to access/download;Figure;IC-EN-00889-01_mask.png

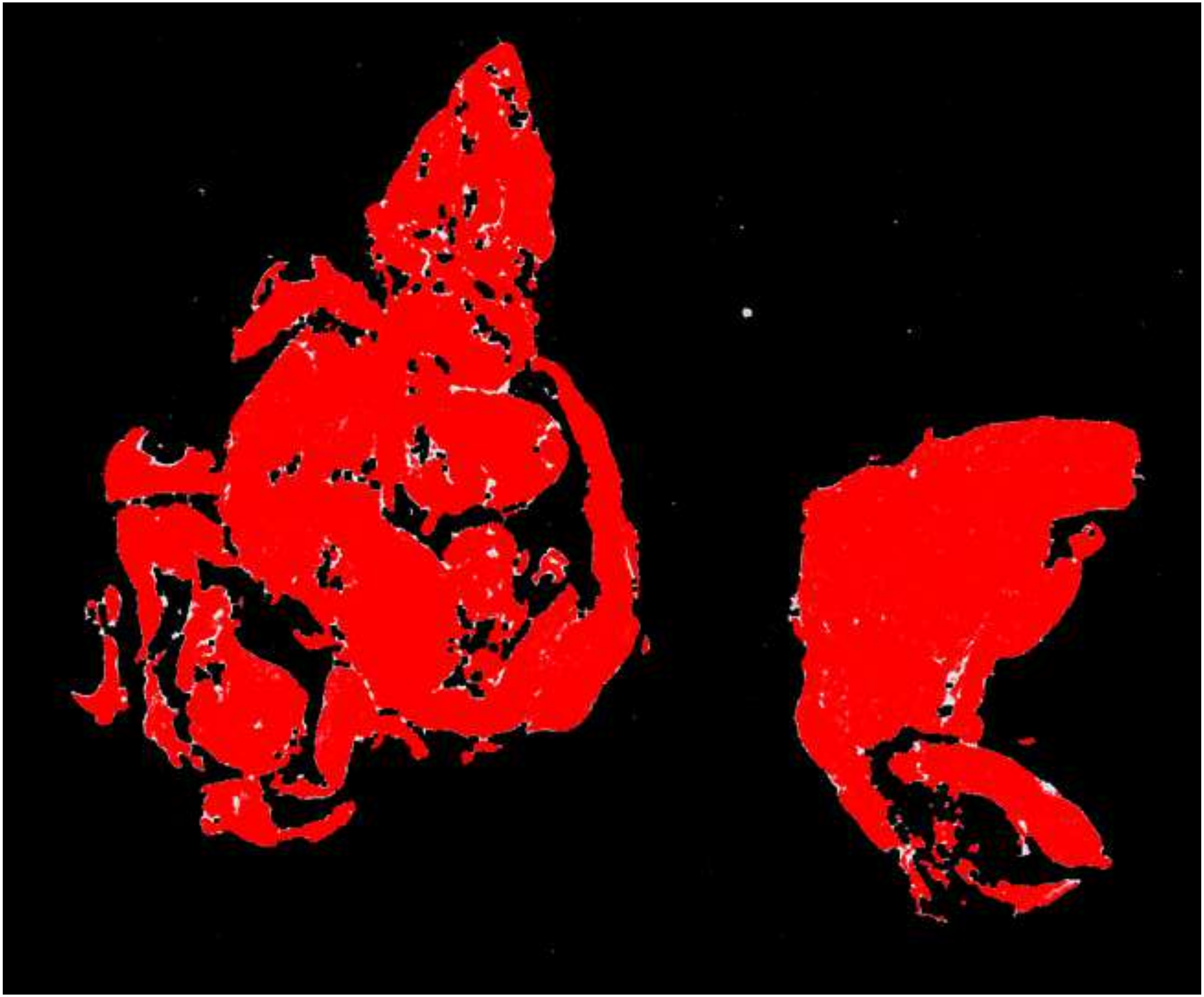

Click here to access/download;Figure;endo flow chart.jpg

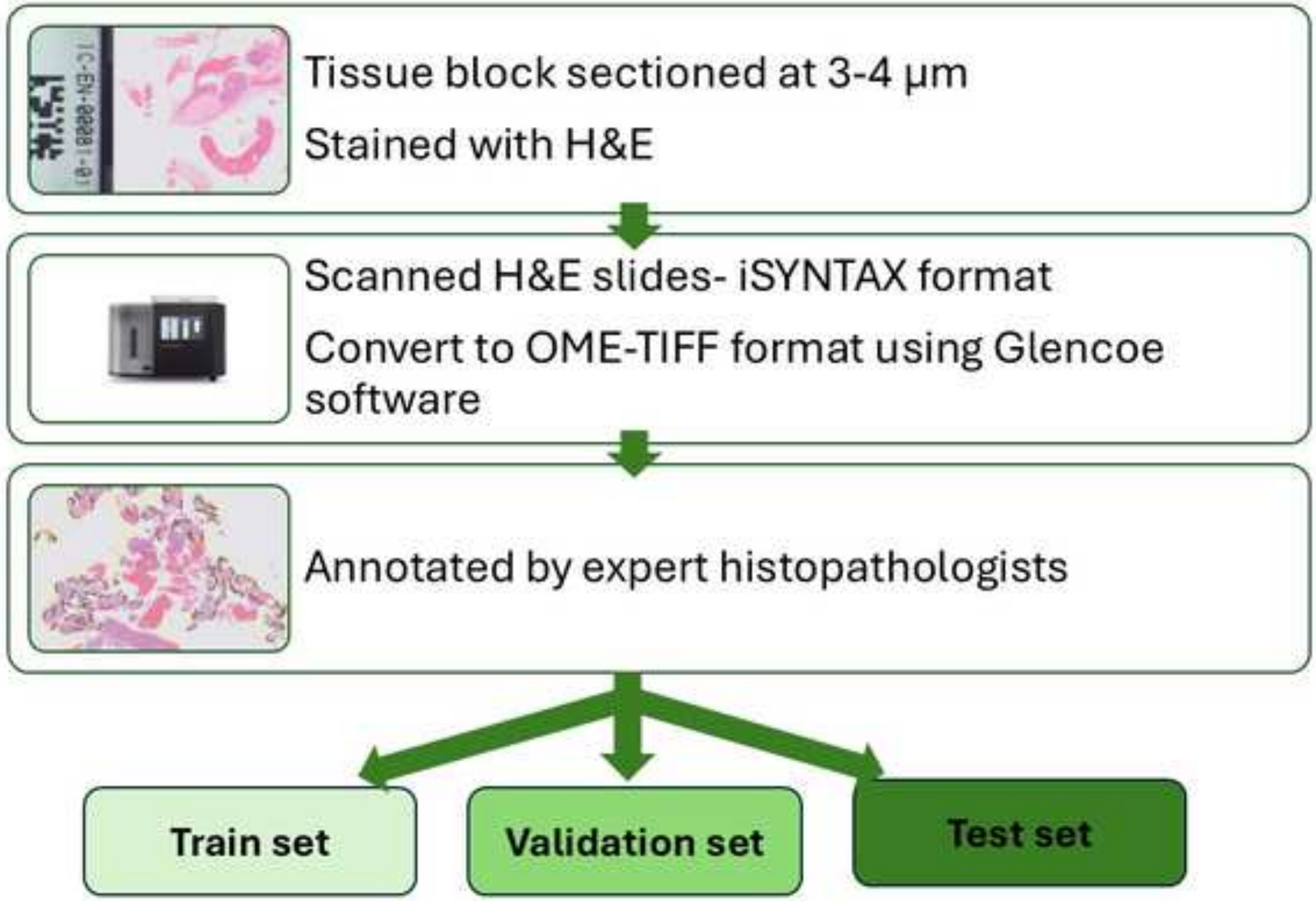

Click here to access/download;Figure;endometrial_subcategorry-smaller7.jpg

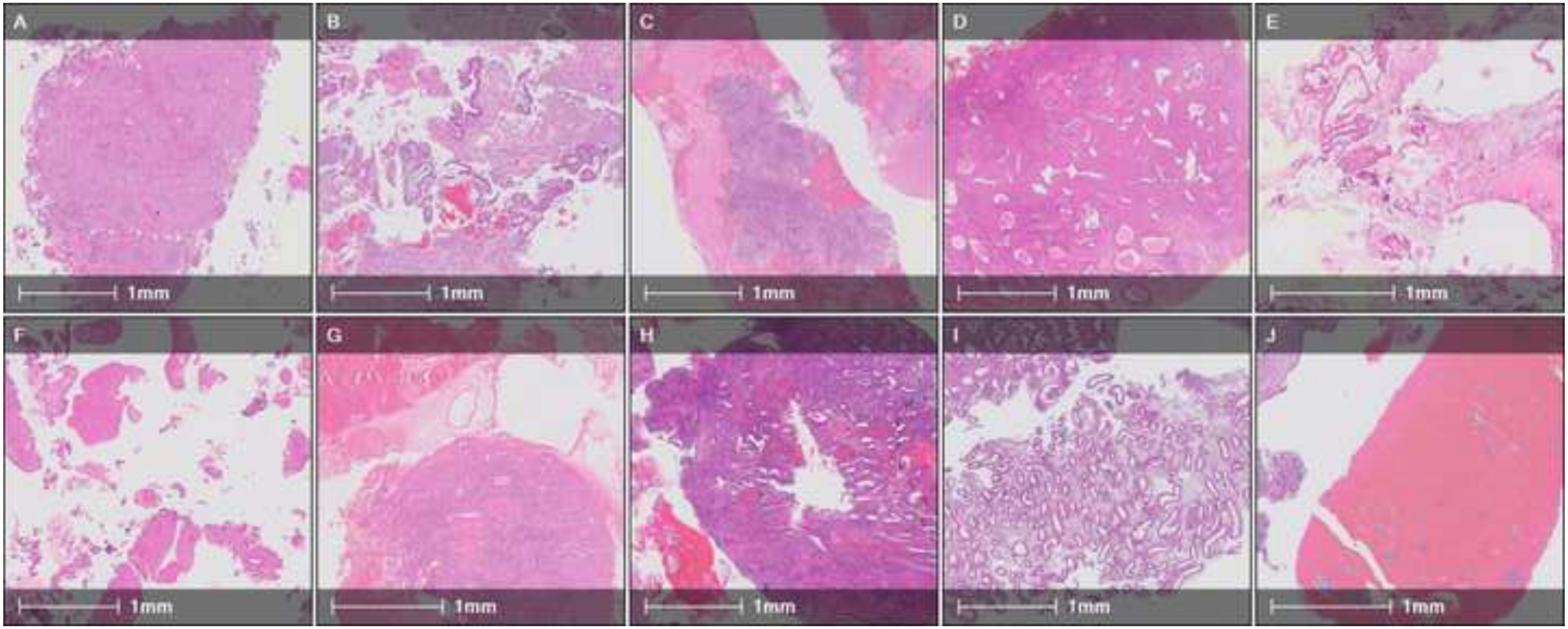

Click here to access/download;Figure;Fig3.png

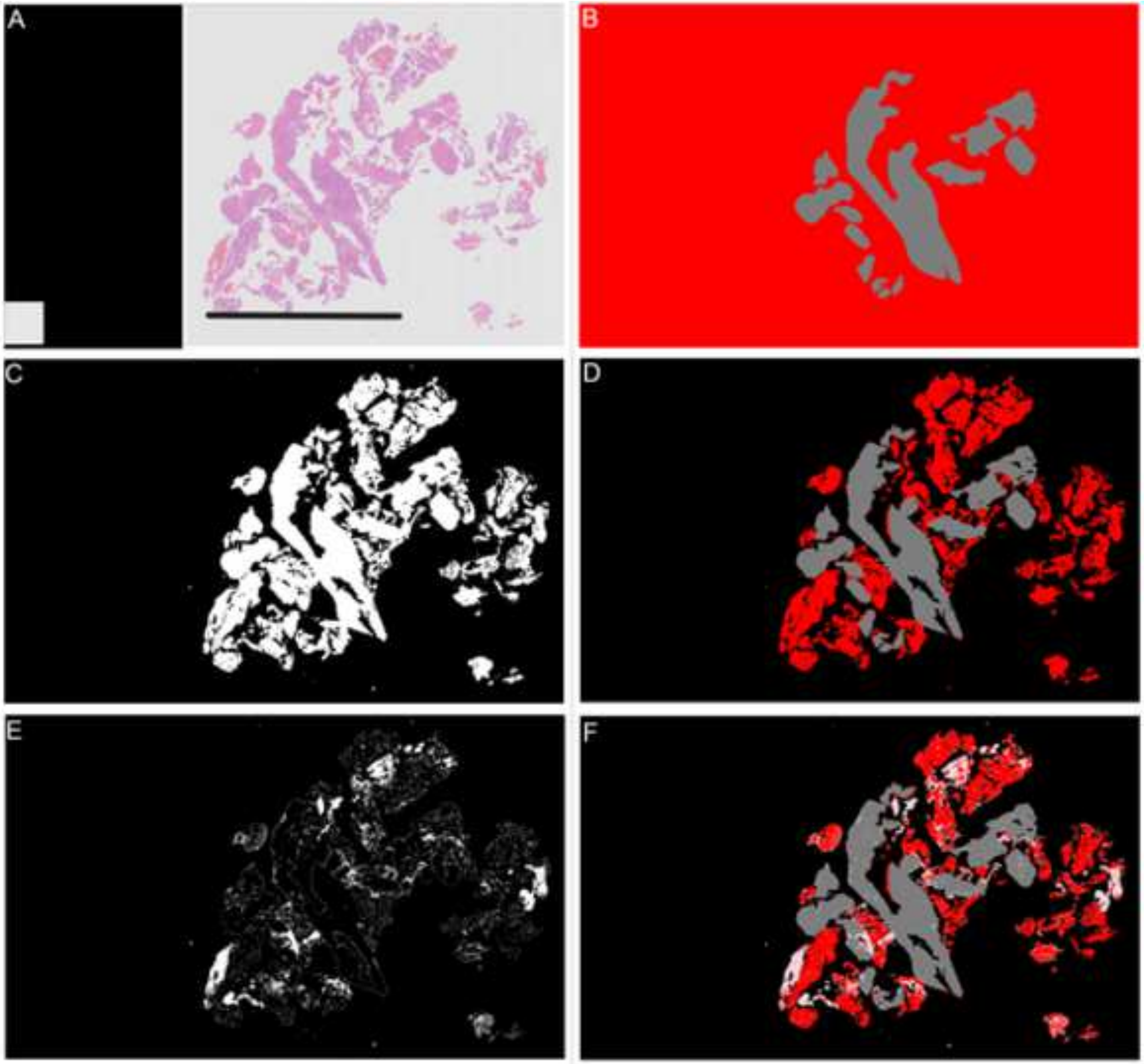

Click here to access/download;Figure;IC-EN-00020-01_thumbnail.png

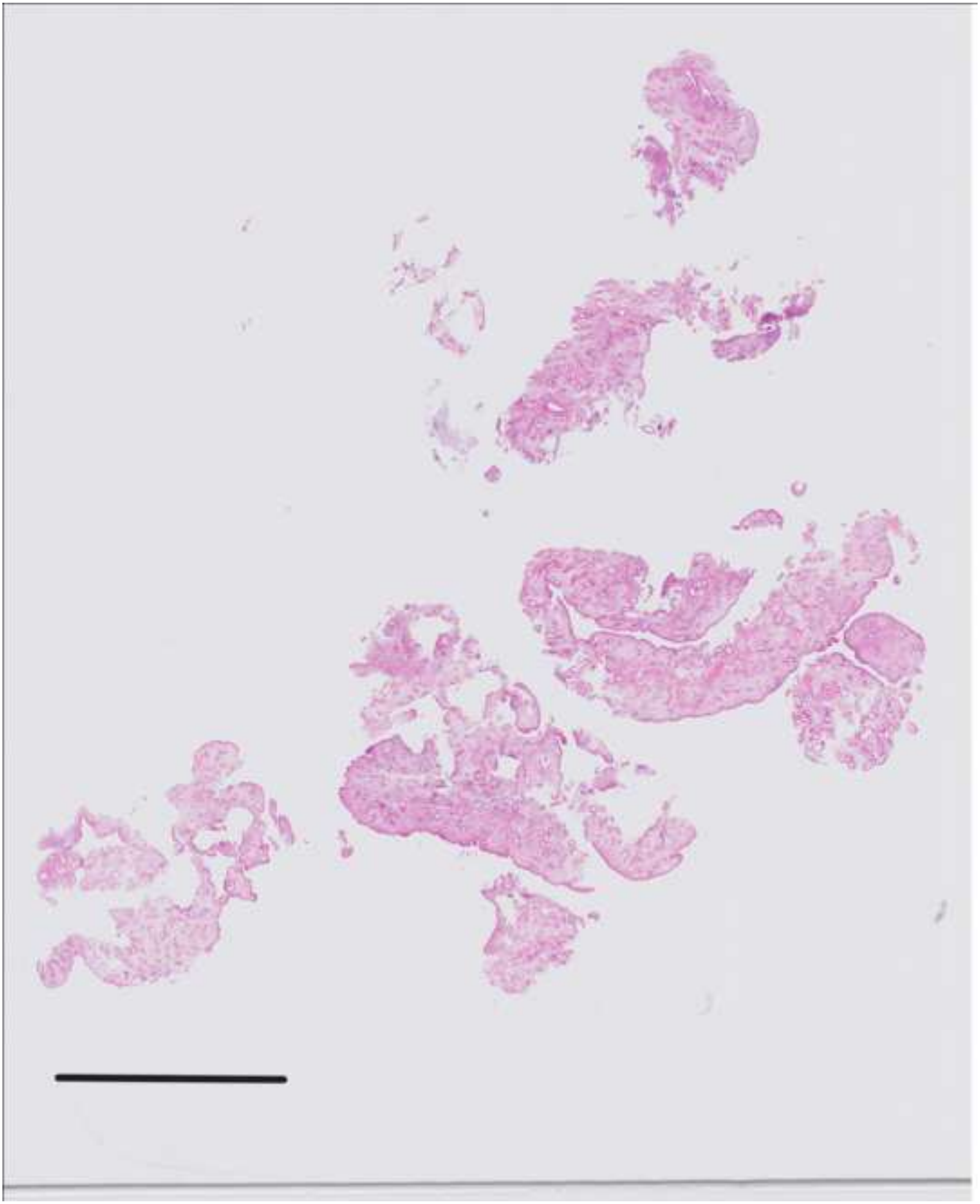

Click here to access/download;Figure;IC-EN-00027-01_thumbnail.png

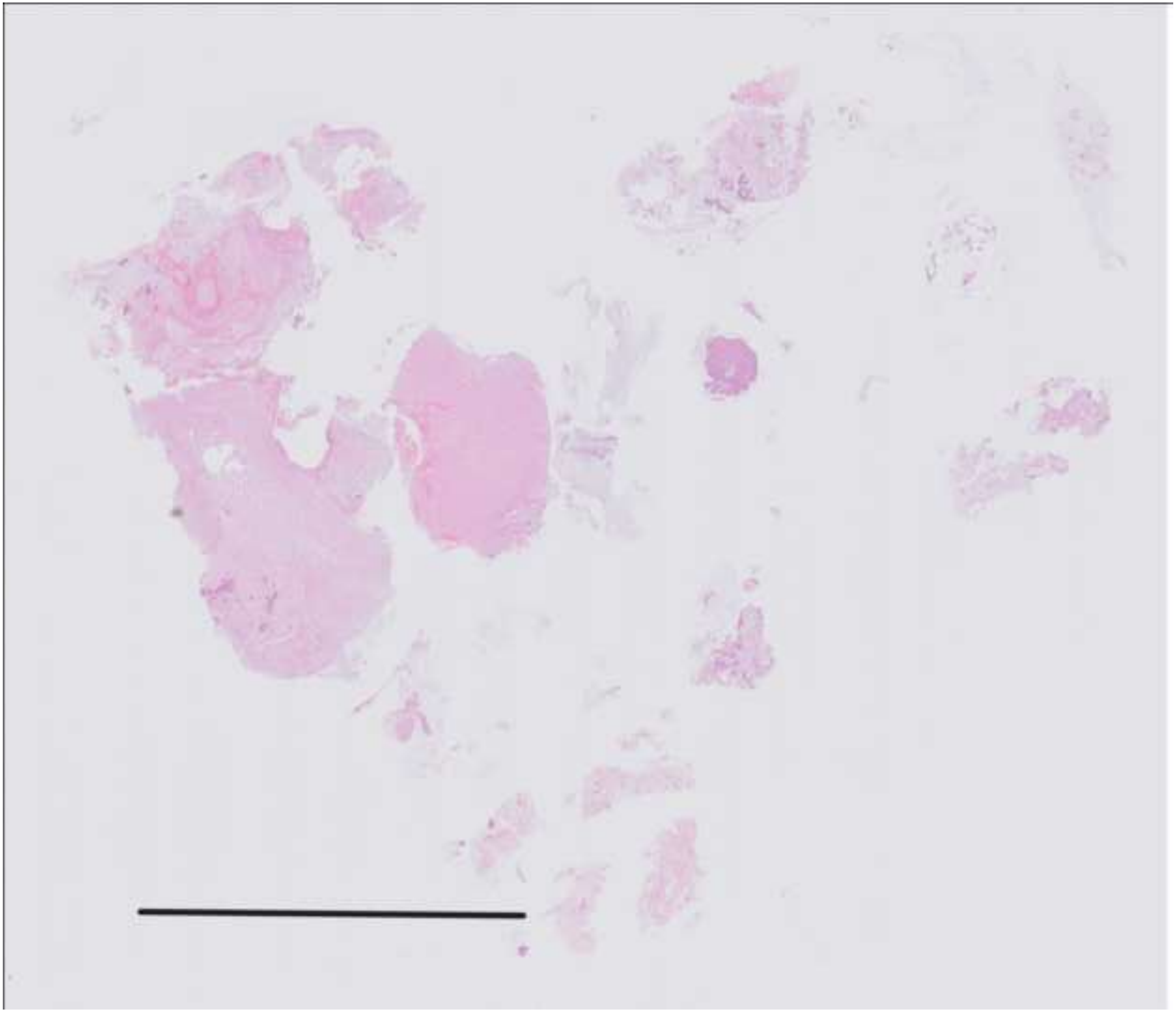

Click here to access/download;Figure;IC-EN-00029-01_thumbnail.png

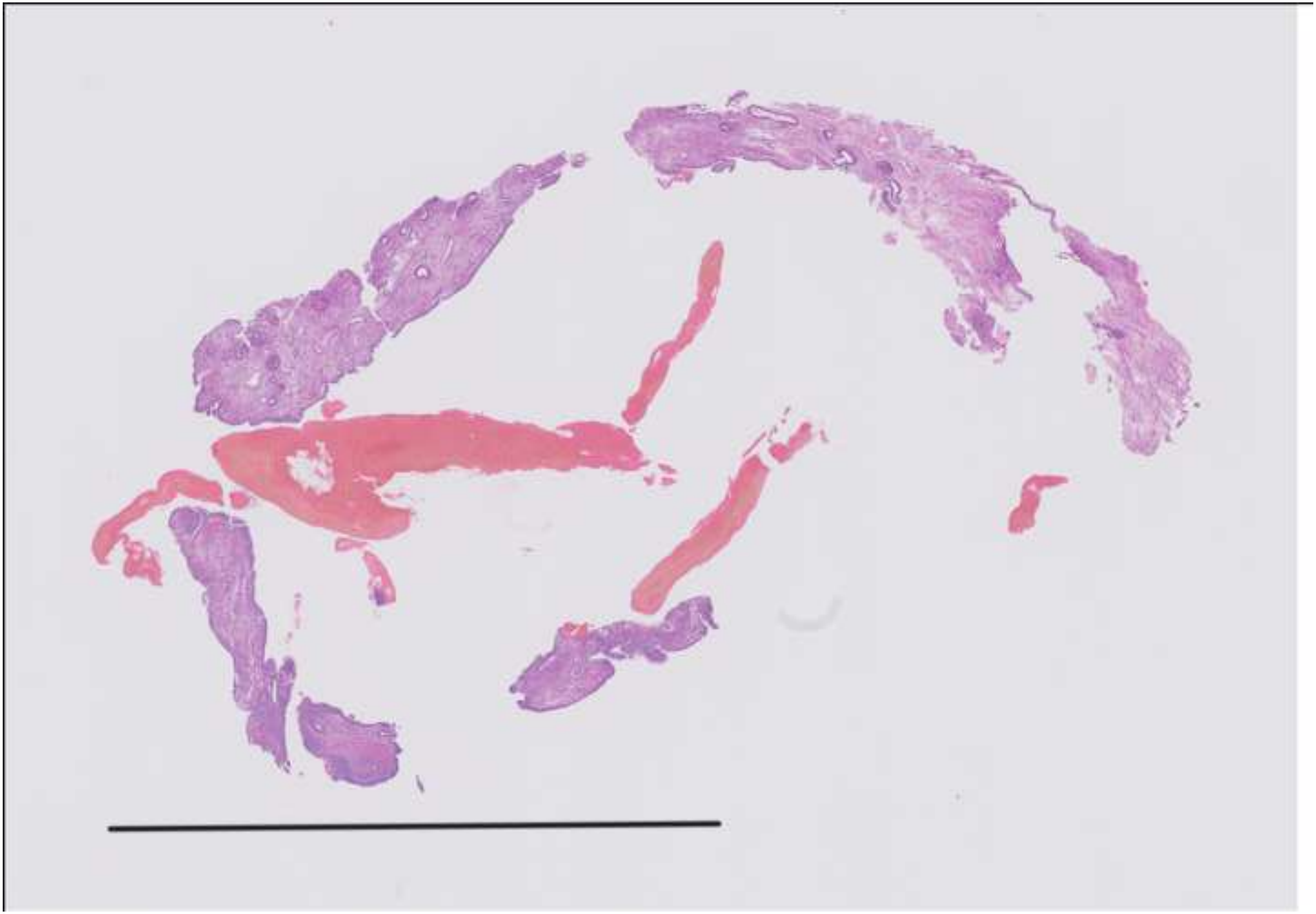

Click here to access/download;Figure;IC-EN-00033-01_thumbnail.png

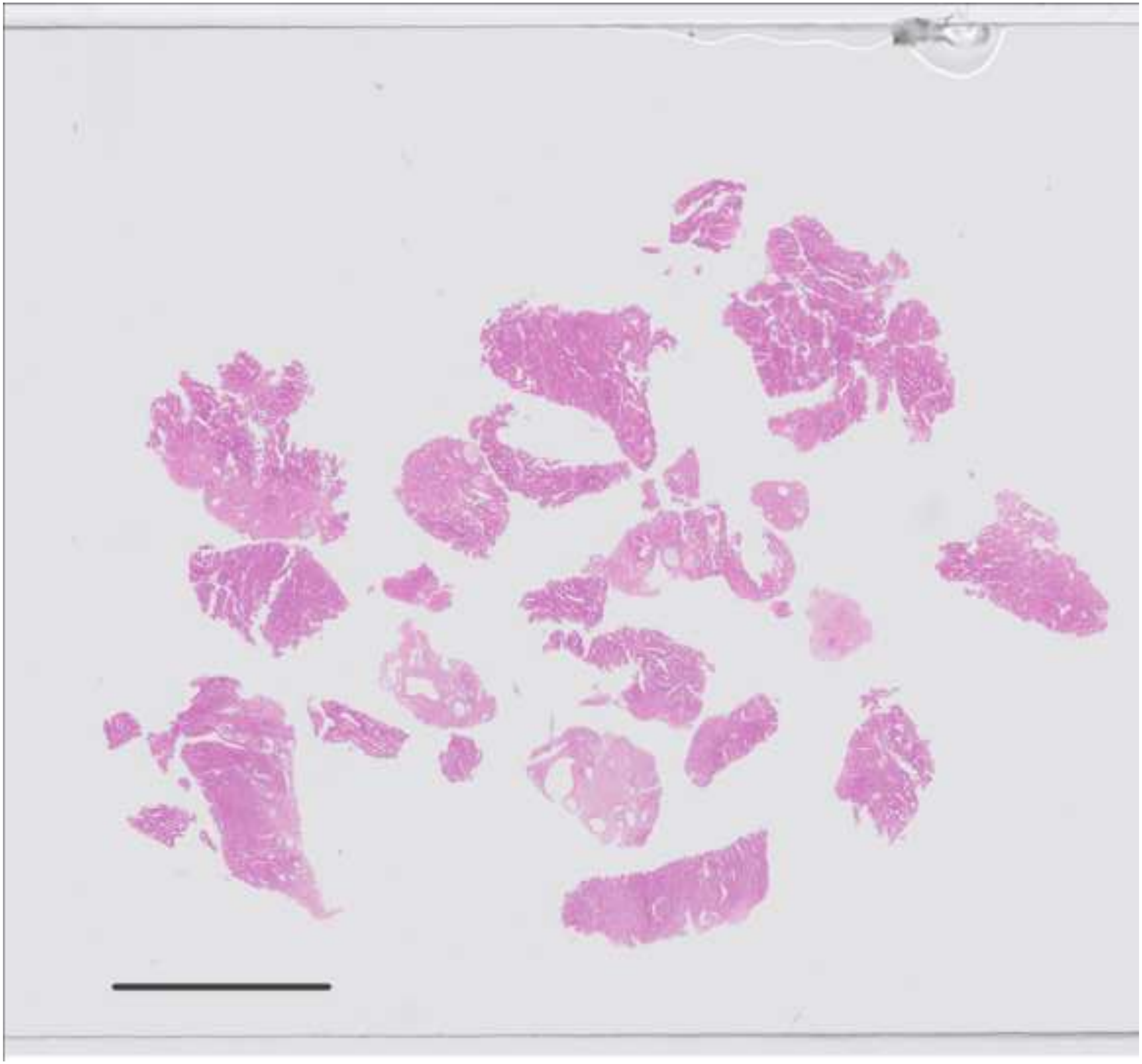

Click here to access/download;Figure;IC-EN-00040-01_thumbnail.png

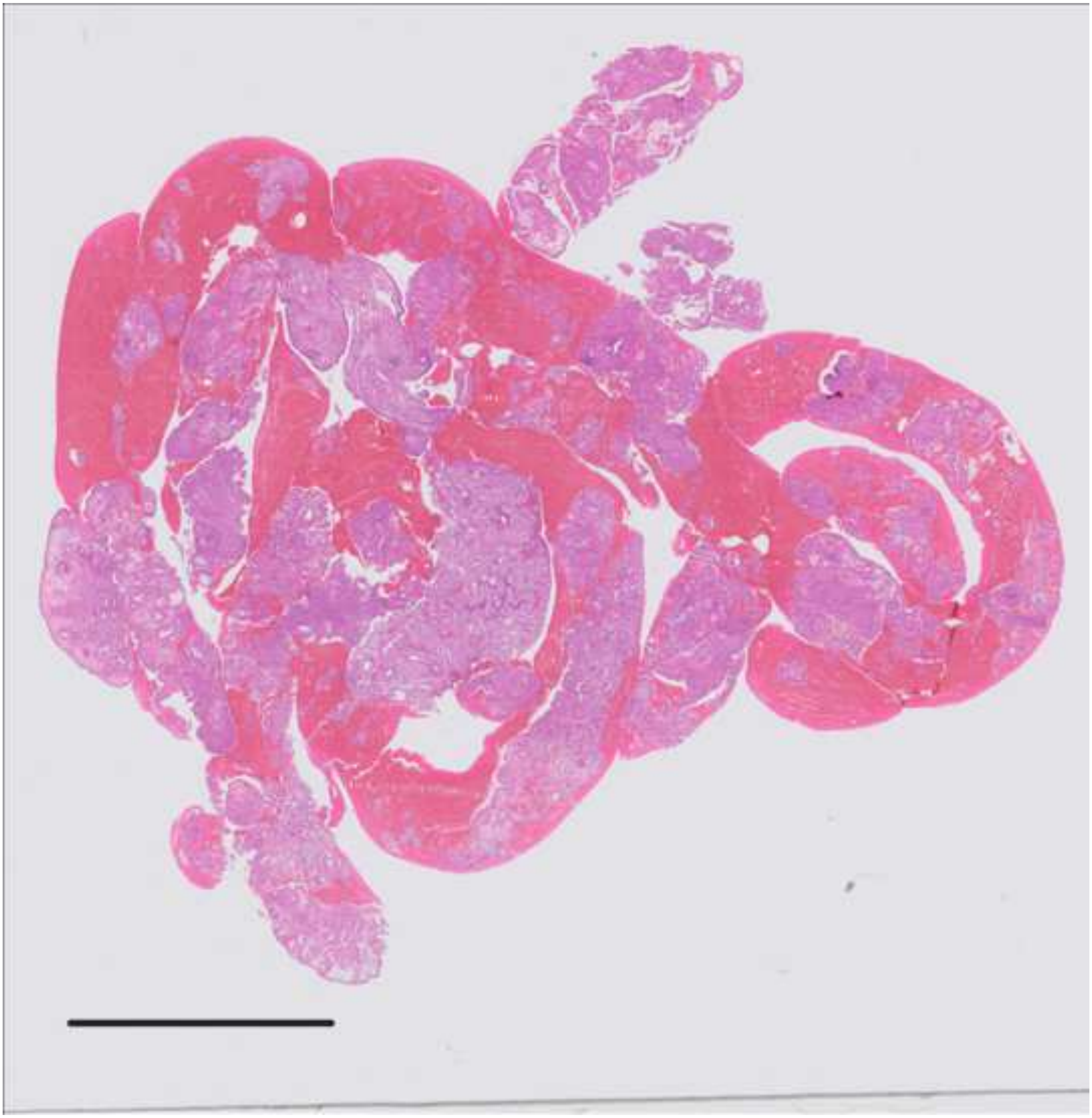

Click here to access/download;Figure;IC-EN-00079-01_thumbnail.png

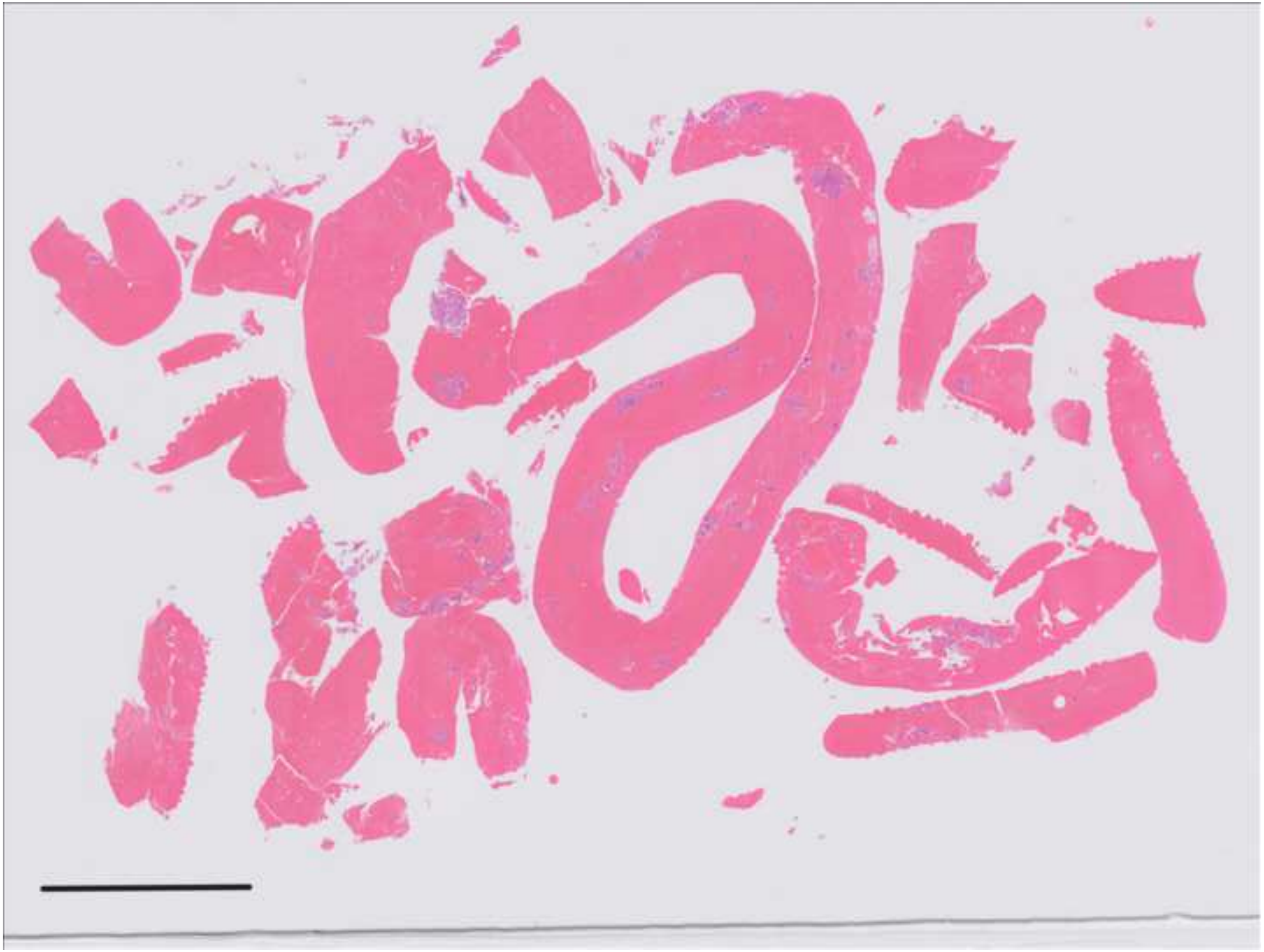

Click here to access/download;Figure;IC-EN-00144-01_thumbnail.png

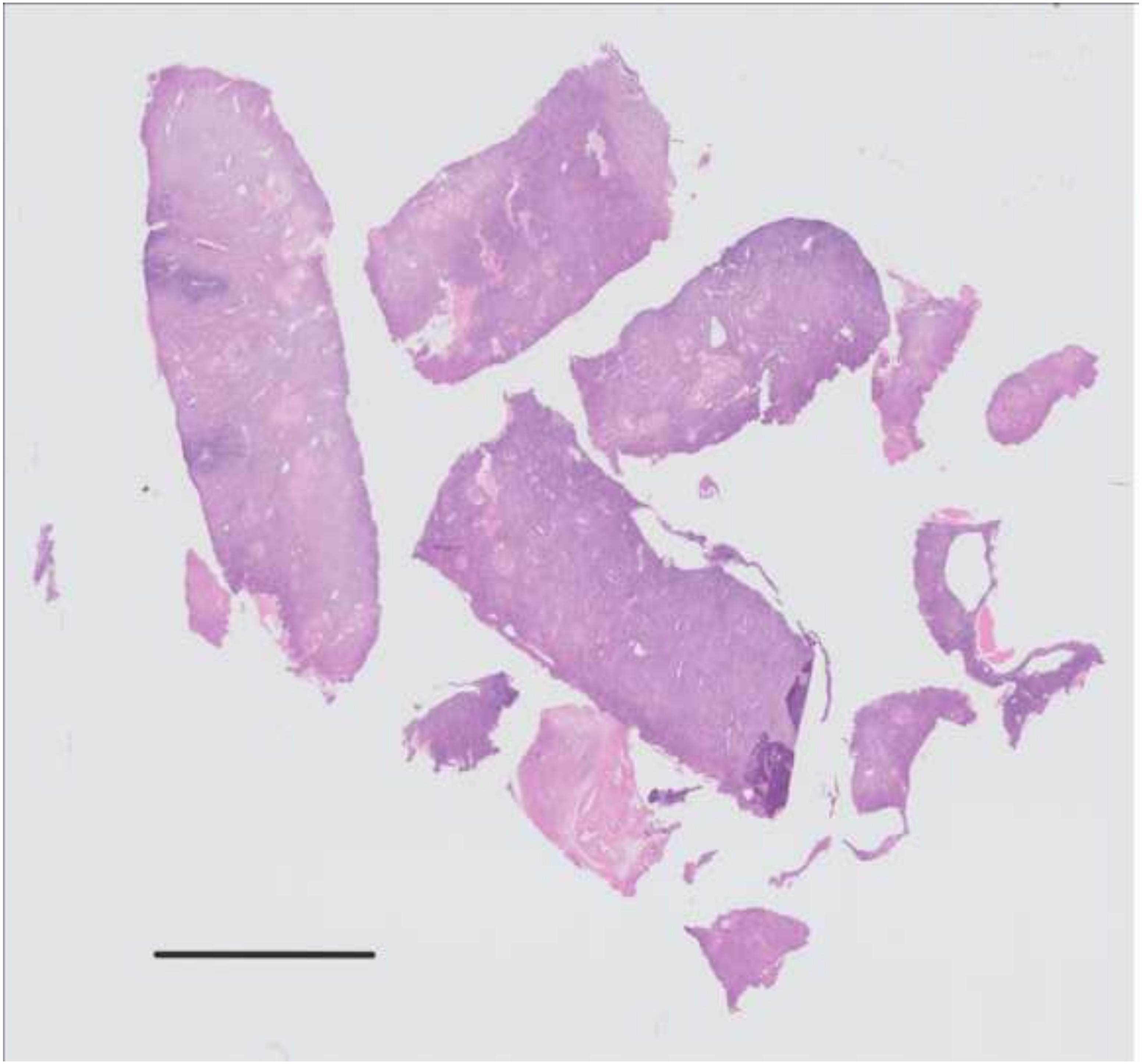

Click here to access/download;Figure;IC-EN-00351-01_thumbnail.png

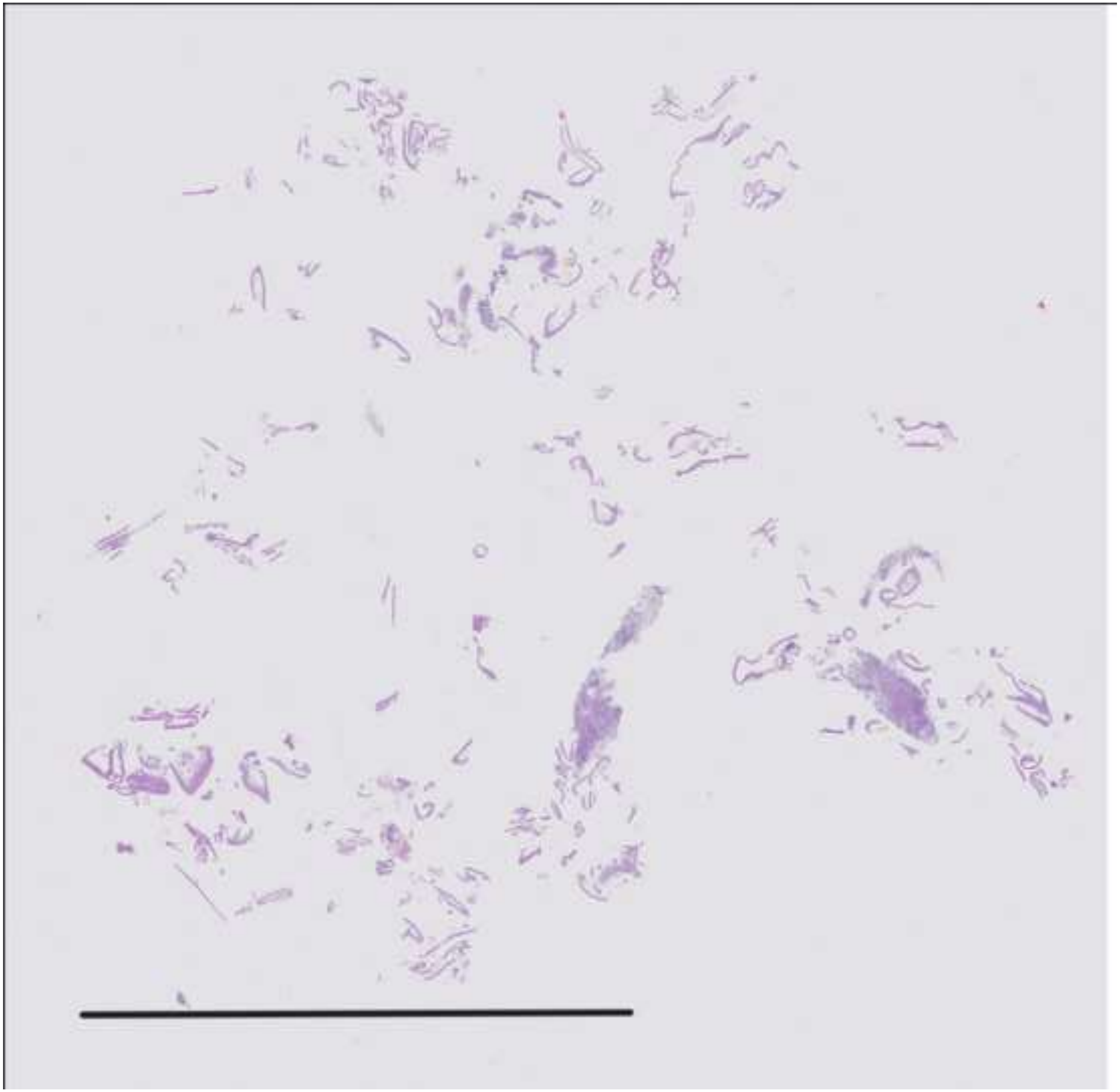

Click here to access/download;Figure;IC-EN-00889-01_thumbnail.png

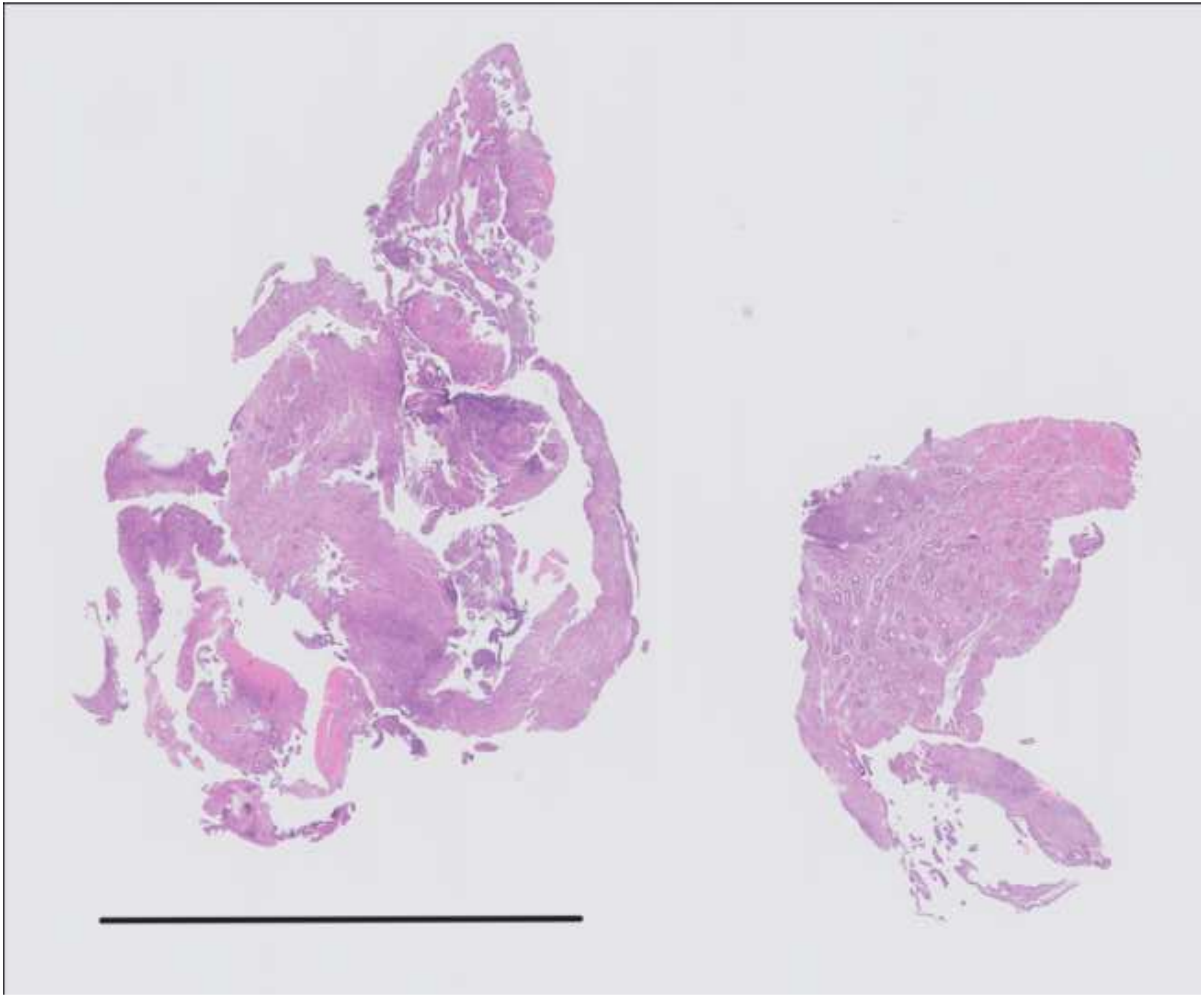

Personal Cover

Click here to access/download;Personal Cover;Giga-Endometrial reviewers comments-13Feb25.docx

Editor GigaScience

School of Medicine
University of St Andrews
North Haugh
St Andrews UK
KY16 9TF

14 February 2025

Dear Editor,

We appreciate the opportunity to revise and resubmit our manuscript, **"Endometrial Whole Slide Images Dataset for Detection of Malignancy in Endometrial Biopsies (ID: GIGA-D-24-00211)".** We are grateful for the reviewers' valuable feedback, which has helped us refine and strengthen our work.

Please find the attached our detailed point-by-point response addressing each of the reviewers' comments, together with a revised version of our manuscript. To enhance the clarity and rigor of our study, we have incorporated the necessary revisions and, in doing so, have added James D. Blackwood as a co-author, reflecting his significant contributions to the manuscript.
We hope that our revisions sufficiently address all concerns and further improve the quality of our work. We are grateful for your time and consideration in reviewing our revised submission and look forward to your feedback.

Yours sincerely,

In Hwa Um

Reviewer #1:

1. ***File format: The most common formats for WSIs are ".svs" and ".tif" and the most common python library used is "OpenSlide". Openslide does not support "isyntax" file format (reference: https://eur01.safelinks.protection.outlook.com/?url=https%3A%2F%2Fopenslide.org%2Fapi%2Fpython%2F&data=05%7C02%7Cihu%40st-andrews.ac.uk%7C0510f47c971843087ded08dcb5449a05%7Cf85626cb0da849d3aa5864ef678ef01a%7C0%7C0%7C638584553449646841%7CUnknown%7CTWFpbGZsb3d8eyJWIjoiMC4wLjAwMDAiLCJQIjoiV2luMzIiLCJBTiI6Ik1haWwiLCJXVCI6Mn0%3D%7C0%7C%7C%7C&sdata=u4y5wFU52ot0TNyDujqU%2BzIinmlKEptlzOQLcH7e9dg%3D&reserved=0). In order to make sure the EC dataset reaches a vast audience, it is recommended to convert to one of the file formats supported by OpenSlide library.***

   Glencoe software was used to convert iSyntax format to ome-tiff, as described in the text. More information on this process can be found at the following link: https://www.glencoesoftware.com/blog/2019/12/09/converting-whole-slide-images-to-OME-TIFF.html
   In preliminary discussions with Dr. Armit before submission, we acknowledged that while the iSYNTAX format can pose certain challenges, it is still possible to convert these images successfully. Additionally, some researchers seek access to Philips-originated images to address and overcome the relative difficulty of working with this format. This highlights the importance of maintaining accessibility to such datasets, as they contribute to the broader goal of improving interoperability and image analysis in the research community.

2. ***Metadata: In order for the community to use this dataset, it would be important to include metadata such as age, survival data, stage, response to treatment, if any treatment given, molecular class or any available metadata. For reference, the authors could use the metadata available for TCGA datasets as a reference.***

   We do not have this information. Sex is obviously female, but in almost every case included the diagnosis would have no consequences for long term clinical outcome. The nature of the conditions being diagnosed and studies means that no molecular genetic testing would be carried out routinely. TCGA has detailed studies of cancer, but many of the cases in our cohort are not cancerous, which is precisely why we believe this is a useful, unique addition to publicly available data.

3. ***Highlighting the utility of datasets: A missing piece in the manuscript is to showcase the real utility of the dataset. This could include comparison with other publicly available EC***

datasets. What does the model performance look like in terms of metrics such as F1 score, precision, recall. If the nuclei features are included, what would the performance look like.

Preliminary results can be found in our cited primary research paper (Fell et al, “Detection of malignancy in whole slide images of endometrial cancer biopsies using artificial intelligence”, https:// doi.org/10.1371/journal.pone.0282577). In this study, we developed and reported an AI model designed to assist in the classification of whole slides images. The model categorises slides into, “malignant, “other or benign” and “insufficient” at the slide level. Additionally, the AI generates heatmaps to highlight specific regions withing the slides that are likely malignant, providing valuable insights for pathologists.
We have not directly compared the performance differences between this AI model and nuclear morphological features. However, we believe that referencing this paper, along with the additional tabular data on nuclear morphological features in reference 14 (https://www.ebi.ac.uk/biostudies/bioimages/studies/S-BIAD1199?query=endometrial), should offer valuable insights since this is the only large study with histological images of endometrial biopsies. These resources can help researchers using the dataset to design their own analyses, formulate hypotheses, and conduct experiments to explore potential relationships between AI-driven classification and nuclear morphology-based assessment. Regarding model performance metrics, they are detailed in the publication. The study provides a baseline evaluation of the model’s diagnostic capabilities, which can serve a foundation for future comparative analyses with other publicly available endometrial cancer (EC) datasets.

4. ***Data distribution: It would be good if authors provided the class label and data split for each of the eight different labs.***

This table shows the data split for eight different laboratories.

StainingSiteID	categopry	train	valid	test
1	other_benign	157	67	26
1	malignant	73	31	10
1	insufficient	12	9	2
2	other_benign	146	74	26
2	malignant	72	30	12
2	insufficient	21	8	0
3	other_benign	144	75	25
3	malignant	61	32	10
3	insufficient	17	5	2
4	other_benign	138	78	23
4	malignant	74	37	12
4	insufficient	14	8	3
5	other_benign	136	70	23
5	malignant	63	33	10
5	insufficient	13	7	2
6	other_benign	0	0	222
6	malignant	0	0	107

6	insufficient	0	0	21
7	other_benign	133	67	22
7	malignant	64	29	11
7	insufficient	16	8	2
8	other_benign	0	0	236
8	malignant	0	0	100
8	insufficient	0	0	18

5. ***In case authors don't already plan to do it, it would be good if the trained blood and mucus detection model is also made available.***

   The complete process is comprehensively described in Fell et al. which we have cited, "Detection of malignancy in whole slide images of endometrial cancer biopsies using artificial intelligence", https:// doi.org/10.1371/journal.pone.0282577. Additionally, the relevant codes supporting this work are publicly available on Zenodo (https://zenodo.org/record/7674764), and this is cited in reference 10.

6. ***Table 1 currently includes the distribution of samples. Since one patient can possibly have more than one sample, it would be good to include distribution of patients as well.***

   There is only one sample per patient.

7. ***Under "annotation process", the authors mention a mix of experts were used for annotations. How many annotators were exactly used for the task?***
   Four histopathologically trained biomedical scientists and four histopathologists annotated biopsies. All histopathologists who annotated biopsies are registered with GMC and Royal College of Pathologists, with subspecialty expertise in gynaecological pathology. Each participating pathologist has a minimum of 5-10 years of post-specialist training experience, with some having over 15 years of expertise in gynaecological pathology. To ensure high-quality and standardised annotations, pathologists reviewed a detailed annotation protocol based on internationally recognised guidelines (WHO classification of tumours, 5th Edition).

8. ***Abstract: The abstract could highlight the class labels and their importance***

   Abstract has been improved as requested.

9. ***It would be good to add "scale" example for 1mm for all images in figures 1-4.***

   It has been done as requested.

Reviewer #2:

10. ***A detailed summary of existing databases related to endometrial cancer should be provided, including but not limited to the TCGA UCEC. Specifically, it is important to highlight the advantages or contributions of the database mentioned in this paper compared to the existing datasets.***

    Our primary research paper (Fell et al, "Detection of malignancy in whole slide images of endometrial cancer biopsies using artificial intelligence", https://doi.org/10.1371/journal.pone.0282577) discusses a publicly available endometrial cancer histology image dataset (https://doi.org/10.7937/k9/tcia.2018.3r3juisw). However, this comprises only 250 cases, whereas our dataset has 2909 cases. There is no comparable dataset covering a wide range of endometrial pathology available as far as we know.

11. ***It would be beneficial to present the data collection procedure and data split details using a flow chart or a table. In the current manuscript, these two sections are not easy to understand.***

    Figure has been added as requested.

12. ***More details about this database should be provided. For instance, include information on the distribution of the proportion of annotated areas in the entire slide, as well as the racial, gender, and age distribution of the whole slide images.***

    The tissue on the slide has been fully annotated by expert pathologists. Patients were obviously female and predominantly Caucasian, but the ethics permission does not include identifiers such as age and race.

13. ***In Table 1, please clarify what the subcategory "Other" refers to. Additionally, explain the potential uses for slides labelled as "insufficient".***

    One problem in diagnostic pathology is how to deal with small samples that may not be representative or provide conclusive evidence for the pathologist. On occasion this may necessitate a report that states "tissue insufficient for diagnosis", which in turn may lead to a further sample being sought. This is a potential distressing and painful procedure for the patient. Being more confident about defining an inadequate sample will help workflow, ensure safety and minimise unnecessary discomfort.

14. ***It would be better to explain what deep learning tasks this database can be utilized for, such as patch classification, whole slide image classification and segmentation. Additionally, briefly describe how to process the annotations for each corresponding task.***

    The database can be used for whole slide image classification or patch classification. Some slides contain a mix of classes and can be used for segmentation.

Section 2.3 of our primary research paper (Fell et al, "Detection of malignancy in whole slide images of endometrial cancer biopsies using artificial intelligence", https://doi.org/10.1371/journal.pone.0282577) which is cited as reference 9 describes how to process annotations.

15. ***To validate the proposed database, benchmark results should be provided. For example, patch classification results using CNN models and WSI classification results using a multiple instance learning model.***

    This was described in the original research paper (Fell et al, "Detection of malignancy in whole slide images of endometrial cancer biopsies using artificial intelligence", https://doi.org/10.1371/journal.pone.0282577).

16. ***It would be better to show typical regions for each subcategory and briefly summarize their differences.***

    Figure has been added as requested.

17. ***In Table 1, the largest number of these subcategory has 518 slides, while the smallest number is only 8. The authors should explain why the number of WSIs in each category is unbalanced.***

    The cases were obtained from a biorepository and reflect the mix of conditions received by the laboratories. Rare things are rare, and so the number of cases is limited! The preponderance of common conditions lends itself to our argument in new abstract that a major challenge in dealing with rising workload in endometrial pathology is filtering out common conditions with confidence rather than necessarily focussing on diagnosing rare conditions.
