# Supplementary material for: Endometrial whole-slide images dataset for detection of malignancy in endometrial biopsies
Source: Gigascience. 2025 Dec 5;14:giaf147. doi: 10.1093/gigascience/giaf147 (PMC12751089; doi:10.1093/gigascience/giaf147)
Supplement: giaf147_GIGA-D-24-00211_Revision_2 [file giaf147_giga-d-24-00211_revision_2.pdf]

GigaScience

Endometrial Whole Slide Images Dataset for Detection of Malignancy in Endometrial Biopsies

--Manuscript Draft--

Manuscript Number:	GIGA-D-24-00211R2
Full Title:	Endometrial Whole Slide Images Dataset for Detection of Malignancy in Endometrial Biopsies
Article Type:	Data Note
Funding Information:	Innovate UK (104690) — Prof David James Harrison
Abstract:	Background: Whole slide imaging (WSI) enables the digitisation of entire histological slides at high resolution, allowing pathologists and researchers to analyse tissue samples digitally rather than through traditional microscopy. This technology has become increasingly valuable in pathology for research, education, and clinical diagnostics. Endometrial biopsy is very common, often being undertaken to exclude non-cancerous disease. This means that most cases do not contain cancer, and the challenge is to accurately and efficiently exclude serious pathology rather than simply make a diagnosis of malignancy. A well-curated, expert-annotated, endometrial whole slide dataset covering a spread of cancer and non-cancer diagnoses will support machine learning applications in automated diagnosis, facilitate research into the pathology of endometrial cancer, and serve as an educational resource for medical professionals. Results: We introduce a newly constructed, large-scale dataset of endometrial biopsies, comprising 2,909 whole slide images in iSyntax format, each accompanied by a corresponding annotation file in JSON format. Each whole slide image is labelled with a primary class label representing its final diagnosis and a sub-category label providing further details within that diagnostic class. These class labels are critical for machine learning applications, as they enable the development of AI models capable of distinguishing between different types of endometrial abnormalities, improving automated classification, and guiding clinical decision-making. Conclusions: Constructing and curating a high-quality endometrial whole slide dataset requires significant effort to ensure accurate annotations, data integrity, and patient privacy protection. However, the availability of a well-annotated dataset with detailed class labels is crucial for advancing digital pathology. Such a resource can enhance diagnostic accuracy, support personalized treatment strategies, and ultimately improve outcomes for patients with endometrial cancer and other endometrial conditions.
Corresponding Author:	In Hwa Um, Ph.D.<br>St Andrews University<br>St Andrews, Scotland UNITED KINGDOM
Corresponding Author Secondary Information:	
Corresponding Author's Institution:	St Andrews University
Corresponding Author's Secondary Institution:	
First Author:	In Hwa Um, Ph.D.
First Author Secondary Information:	
Order of Authors:	In Hwa Um, Ph.D.
	Mahnaz Mohammadi
	Christina Fell
	Sarah Bell
	Gareth Bryson
	Sheeba Syed

Powered by Editorial Manager® and ProduXion Manager® from Aries Systems Corporation

	Prakash Konanahalli
	David Harris-Birtill
	Ognjen Arandjelovic
	Clare Orange
	Prishma Shahi
	David James Harrison
	James D Blackwood
**Order of Authors Secondary Information:**	
**Response to Reviewers:**	Reviewer #2:<br>1.Regarding my previous Comment 11, the authors have indeed provided a chart to present the data collection procedure and data split details. However, the chart is overly simplistic and should include more details. These details should include, but are not limited to, the inclusion and exclusion criteria for patients and the quality control standards for WSIs. Additionally, the data split details should clarify how the consistency of distribution across the training, validation, and test sets is maintained.<br><br>We thank the reviewer for the helpful suggestion. In response, we have revised the manuscript to expand the description of the data collection and dataset splitting procedures and updated the associated chart (Figure 1) to reflect these details more comprehensively.<br>As this Data Note is derived from our previously published study in PLOS ONE (https://doi.org/10.1371/journal.pone.0282577), we provide further clarification regarding the dataset creation and structure. Cases were retrospectively identified by querying the pathology department archives using the topographic term “endometrium.” Only slides that had been formally reported by a diagnostic pathologist were included. This ensured that the material met the quality threshold necessary for issuing a clinical diagnosis. Where required, additional histologic sections were prepared to ensure diagnostic adequacy. Subsequently, the study pathologists independently reviewed the candidate slides and confirmed that the diagnostic label could be reliably established based on material that was technically sufficient for clinical assessment.<br>To ensure the quality of the whole slide images (WSIs), a multi-stage quality control process was followed. Initially, slides underwent quality screening during routine diagnostic workflows. After scanning, each WSI was visually inspected to verify focus, resolution, staining adequacy, and absence of scanning artifacts. WSIs with poor image quality, insufficient tissue, incomplete metadata, or uncertain diagnosis were excluded. This dual-level review, involving both clinical and research pathologists, ensured that only high-quality and diagnostically reliable slides were included in the dataset. The slides were categorized into three diagnostic classes: “malignant,” “other or benign,” and “insufficient.”<br>To ensure the model's ability to generalize across different laboratory protocols, the dataset was divided based on the originating laboratories' staining protocols. Specifically, the test set included all slides from two laboratories not represented in the training and validation sets, accounting for approximately 75% of the test data. Additionally, 10% of slides from the remaining six laboratories were randomly selected and added to the test set, making up the remaining 25%. The remaining 90% of slides from these six laboratories were split into training and validation sets, with two-thirds allocated for training and one-third for validation. This stratified splitting ensured balanced representation across diagnostic categories and subcategories, facilitating robust model training and evaluation.<br>This structured approach to dataset division was designed to assess both in-distribution and out-of-distribution performance, providing insights into the model's generalisability across varying staining protocols and laboratory settings.<br>The updated chart (Figure 1) now reflects the complete case selection pathway, inclusion and exclusion criteria, multi-level quality control, and the principles applied in the dataset split. We believe that these revisions enhance the transparency and reproducibility of our dataset and address the reviewer’s concerns regarding the simplicity of the initial figure.

	2.Regarding my previous Comment 15, benchmark results for this dataset should be provided. The authors argue that these results were presented in a previous paper. I recommend that they summarize those results and present them appropriately in this manuscript. Additionally, the results in the previous paper pertain to the three-class classification of "malignant/other_benign/insufficient." I suggest that the authors also provide benchmark results for sub-category classification and WSI segmentation in this manuscript, as this would help highlight the utility of the dataset.<br><br>In response to the reviewer's request for benchmark results, we have summarized the classification performance reported in our previously published study, which evaluated the dataset using three standard machine learning approaches: Random Forest, XGBoost, and a Convolutional Neural Network (CNN). The models were trained to perform a three-class classification task distinguishing "malignant," "other or benign," and "insufficient" whole slide images (WSIs).<br>Among these, the CNN achieved the highest accuracy in identifying malignant cases, with classification accuracy ranging from 89.8% to 92.1% depending on whether any tissue patches or majority-tissue patches were used. However, its overall accuracy (85.2%–90.8%) was slightly lower than that of the Random Forest model, which yielded the highest overall accuracy but underperformed in correctly identifying malignant cases. XGBoost provided a balanced performance, intermediate between the CNN and Random Forest classifiers. These results, presented in detail in Table 3 and Figure 9 of the original publication (PLOS ONE, https://doi.org/10.1371/journal.pone.0282577), offer a benchmark for future method development and validation.<br>We agree with the reviewer that presenting benchmark results beyond the three-class task would enhance the manuscript. While our initial work did not include experiments on sub-category classification or WSI segmentation, the dataset is structured to support both. Diagnostic subcategories are embedded within the dataset's metadata, allowing for fine-grained classification tasks, such as differentiating specific benign or malignant subtypes. Similarly, the high-resolution WSIs, combined with associated diagnostic labels and annotations, make the dataset well-suited for training segmentation models aimed at identifying regions of diagnostic relevance.<br>We recognize the importance of these tasks for demonstrating the broader utility of the dataset and plan to pursue them in future work. Incorporating benchmark results for sub-category classification and WSI segmentation will help further establish this dataset as a robust resource for computational pathology research. This has been added in 'Re-Use potential' section. We appreciate the reviewer's suggestion and have revised the manuscript to include this summary and contextual discussion.<br><br>3.Although the authors have revised the manuscript, they did not mark the changes, making it difficult for me to follow the specific revision. As a result, it is hard to determine whether the revised manuscript adequately addresses my concerns. I recommend that the authors highlight the changes in the manuscript.<br><br>We apologise for any inconvenience caused by the revision. Unfortunately, the Overleaf template does not allow us to highlight the changes directly.
**Additional Information:**	
**Question**	**Response**
Are you submitting this manuscript to a special series or article collection?	No
**Experimental design and statistics**<br><br>Full details of the experimental design and statistical methods used should be given in the Methods section, as detailed in our Minimum Standards Reporting Checklist. Information essential to interpreting the data presented should be made available	Yes

Powered by Editorial Manager® and ProduXion Manager® from Aries Systems Corporation

in the figure legends.<br><br>Have you included all the information requested in your manuscript?	
**Resources**<br><br>A description of all resources used, including antibodies, cell lines, animals and software tools, with enough information to allow them to be uniquely identified, should be included in the Methods section. Authors are strongly encouraged to cite Research Resource Identifiers (RRIDs) for antibodies, model organisms and tools, where possible.<br><br>Have you included the information requested as detailed in our Minimum Standards Reporting Checklist?	Yes
**Availability of data and materials**<br><br>All datasets and code on which the conclusions of the paper rely must be either included in your submission or deposited in publicly available repositories (where available and ethically appropriate), referencing such data using a unique identifier in the references and in the "Availability of Data and Materials" section of your manuscript.<br><br>Have you have met the above requirement as detailed in our Minimum Standards Reporting Checklist?	Yes

Powered by Editorial Manager® and ProduXion Manager® from Aries Systems Corporation

Manuscript

Click here to access/download;Manuscript;Endometrial_Data_Note_GigaSci

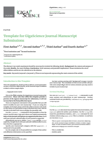

GigaScience, 20xx, 1–9

doi: xx.xxxx/xxxx
Manuscript in Preparation
Data Note

DATA NOTE

Endometrial Whole Slide Images Dataset for Detection of malignancy in endometrial biopsies

Mahnaz Mohammadi[1,*,§], Christina Fell[1,*], Sarah Bell[2,†], Gareth Bryson[2,†], Sheeba Syed[2,†], Prakash Konanahalli[2,†], David Harris-Birtill[4,*], Ognjen Arandjelovic[4,§], Clare Orange[1,2,*], Prishma Shahi[1,*], In Hwa Um[1,3,§], James D Blackwood[1,*] and David J Harrison[1,3,*]

[1]School of Medicine, University of St Andrews, North Haugh, KY16 9TF, United Kingdom and [2]Department of Pathology, Queen Elizabeth University Hospital, Govan Road, G51 4TF, Glasgow, United Kingdom and [3]Pathology, Division of Laboratory Medicine, Royal Infirmary of Edinburgh, Old Dalkeith Road, EH16 4SA, United Kingdom and [4]School of Computer Science, University of St Andrews, North Haugh, KY16 9SX, United Kingdom

[*]mm459, cmf21, dcchb, oa7, celo1, ps289, ihu, jdb20, david.harrison@st-andrews.ac.uk
[†]Sarah.Bell, Gareth.Bryson, sheeba.syed, prakash.konanahalli@ggc.scot.nhs.uk
[§]Corresponding author: mahnaz.mohammadi0@gmail.com, ihu@st-andrews.ac.uk

Abstract

Background: Whole slide imaging (WSI) enables the digitisation of entire histological slides at high resolution, allowing pathologists and researchers to analyse tissue samples digitally rather than through traditional microscopy. This technology has become increasingly valuable in pathology for research, education, and clinical diagnostics. Endometrial biopsy is very common, often being undertaken to exclude non-cancerous disease. This means that most cases do not contain cancer, and the challenge is to accurately and efficiently exclude serious pathology rather than simply make a diagnosis of malignancy. A well-curated, expert-annotated, endometrial whole slide dataset covering a spread of cancer and non-cancer diagnoses will support machine learning applications in automated diagnosis, facilitate research into the pathology of endometrial cancer, and serve as an educational resource for medical professionals. **Results:** We introduce a newly constructed, large-scale dataset of endometrial biopsies, comprising 2,909 whole slide images in iSyntax format, each accompanied by a corresponding annotation file in JSON format. Each whole slide image is labelled with a primary class label representing its final diagnosis and a sub-category label providing further details within that diagnostic class. These class labels are critical for machine learning applications, as they enable the development of AI models capable of distinguishing between different types of endometrial abnormalities, improving automated classification, and guiding clinical decision-making. **Conclusions:** Constructing and curating a high-quality endometrial whole slide dataset requires significant effort to ensure accurate annotations, data integrity, and patient privacy protection. However, the availability of a well-annotated dataset with detailed class labels is crucial for advancing digital pathology. Such a resource can enhance diagnostic accuracy, support personalized treatment strategies, and ultimately improve outcomes for patients with endometrial cancer and other endometrial conditions.

Key words: Endometrium; whole slide imaging; endometrial cancer; endometrial hyperplasia; endometrial carcinoma; digital slide repository; image analysis; image segmentation; histopathology; deep learning; machine learning.

Data Description

The endometrial dataset described in this paper, includes a total of 2909 H&E stained WSIs from NHS Greater Glasgow and

Compiled on: June 27, 2025.
Draft manuscript prepared by the author.

Clyde Biorepository and Pathology with a total of 3.6 TB storage. This dataset was originally created as part of Industrial Centre for iCAIRD [1] with the aim to automatically sort histopathology whole slide images of endometrial biopsies into one of three categories, "malignant", "other or benign" or "insufficient". This would allow prioritisation of malignant slides within the pathologists' workload and reduce the time to diagnosis for patients with cancer.

Context

As the demand for Artificial Intelligence (AI) services continues to grow, so does the need for high-quality datasets. Data is the key component of any Machine Learning (ML) and deep learning projects. The quality of data is as important as the quantity and hence data preparation and understanding is one of the most important and time-consuming tasks of the Machine Learning project life cycle.

Machine learning in healthcare can be used for better diagnosis using ML-enabled tools to analyse medical reports and images. The use of AI in clinical practice aid pathologists in many ways. Techniques like digital image analysis and machine learning are excellent in predicting cancer outcomes. These AI models can help with pathological diagnosis and train pathologists to identify areas of interest in tissue samples.

Endometrial cancer is a type of cancer that originates in the lining of the uterus, which is called the endometrium. It is one of the most common forms of cancer that affects the female reproductive system. The endometrium is the tissue that undergoes changes throughout the menstrual cycle and is shed during menstruation.

Using AI for the detection of endometrial cancer has shown promising results in recent research and clinical applications. AI techniques, such as machine learning and deep learning, can be applied to medical imaging and clinical data to aid in early detection and accurate diagnosis of endometrial cancer.

A recent review of artificial intelligence in gynecological cancers [2] found 13 papers for endometrial cancer, out of which only one paper used H&E WSIs from endometrial biopsies [3]. In this paper a CNN was trained on patches of size 640×640 pixels extracted from the regions annotated by pathologists as normal or malignant. Convolutional Neural Network (CNN) differentiated patches as endometrial adenocarcinoma and 3 benign classes, normal, endometrial polyp, and endometrial hyperplasia and achieved 93.5% accuracy on the binary classification task and 78.0% sensitivity. The results presented in this paper are at the patch level only and no slide level classification has been reported.

A endometrial cancer H&E slides dataset, CPTAC [4] is available on cancer imaging archive, consisting of pathology slides along with genomics data and radiology images. The three studies that used CPTAC aimed to predict the same information as genetic sequencing [5] or illustrate features in H&E slides that could identify different cancer variants [6, 7] and hence allow more personalised treatment.

A weakly supervised learning method used this endometrial dataset for whole slide image diagnosis and interpretability. Interpretability methods including attention heatmapping, feature visualisation, and a novel end-to-end saliency-mapping, were applied to identify distinct morphologies learned by the model and build an understanding of its behaviour [8]. The reported results in this article shows slide level validation and test accuracies over 85% and 87% respectively. This dataset also has been used for detection of malignancy using AI in a recent article [9]. In this article, a fully supervised CNN model was trained to automatically sort endometrial biopsy images into "malignant", "other or benign" or "insufficient" tissue classes with the aim to allow prioritisation of these slides in a queue for pathologist review and hence reduce time to diagnosis for patients with cancer. The final model was able to accurately classify 90% of all slides correctly and 97% of slides in the malignant class; this accuracy is good enough to allow prioritisation of the workload. The code and trained model for this paper is available at [10].

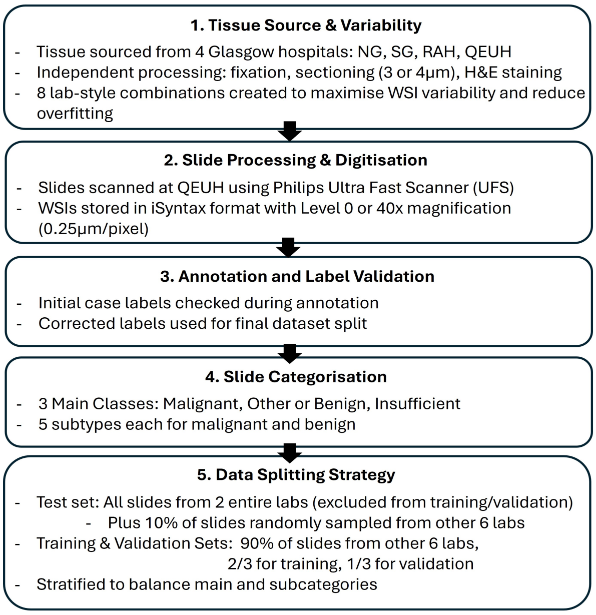

Figure 1. Flow chart of Data Collection and Data Split

Methods

Data collection

The tissue blocks for this study originate from Glasgow Royal Infirmary (NG), Southern General Hospital (SG), Royal Alexandria Hospital (RAH) and Queen Elizabeth University Hospital (QEUH) (all in Glasgow, Scotland) each with independent tissue handling including fixation and tissue processing. New tissue sections were cut from the tissue blocks at one of two different thicknesses (3 or 4 microns) and then stained with one of four different H&E protocols. Together, these combinations gave eight different labs maximising WSI variance and thereby decreasing the likelihood of overfitting to any one lab (combination of tissue processing, cutting and staining protocol) ((Figure 1).

Data split to train and test sets

The slides were split into training, validation, and test sets. The samples had examples of five "malignant" subcategories, five "other or benign" subcategories, and a category "insufficient", where there was insufficient tissue to make a diagnosis. Hyperplasia with atypia was included in the "malignant" category as it is a high risk pre-invasive lesion which it is important to detect (Figure 2).

The test set contained the complete groups of slides for two of the labs and these slides were not part of the training and validation sets. The test set then also contained a randomly selected 10% of the slides from the other 6 labs. The remaining 90% of the slides, from the other 6 labs were used for the training and validation sets. Two thirds of these slides were selected randomly for the training set and the rest were used for the validation set. The splits into the test, validation, and training sets were checked to see that there was a balance of the categories and subcategories across the sets. These splits were calculated based on the case labels associated with the samples recorded in the system. During the annotation process these labels were doubled checked and in approximately 5% of the cases the final label associated with the scanned slide was different. This could be because the new slice taken from the sample did not show the same pathology as the original or that the original label

Table 1. Distribution of samples in training, validation, and test sets for endometrial dataset.

Category	SubCategory	Training	Validation	Test	Total
Malignant	- Adenocarcinoma	243	113	162	518
	- Carcinosarcoma	37	18	28	83
	- Sarcoma	11	6	8	25
	- Hyperplasia with atypia	106	53	67	226
	- Other	4	1	3	8
	Total	401	191	268	860
Other or benign	- Hormonal	158	79	115	352
	- Inactive atrophic	170	90	133	393
	- Proliferative	184	81	116	381
	- Secretory	176	91	116	383
	- Menstrual	159	84	111	354
	Total	847	425	595	1867
Insufficient	- Insufficient	90	44	48	182

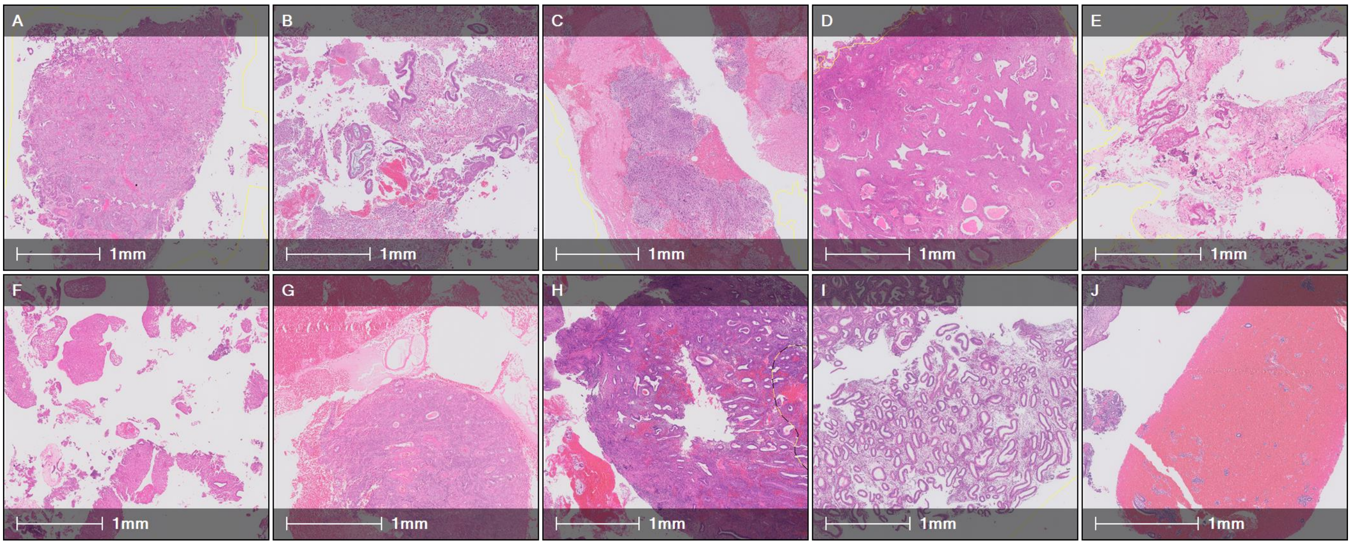

Figure 2. Example of subcategory of malignant and other or benign cases
A. Malignant-Adenocarcinoma, B. Malignant-Carcinosarcoma, C. Malignant-Sarcoma, D. Malignant-Hyperplasia with atypia, E. Malignant-Other, F. Other or Benign-Hormonal, G. Other or Benign-Inactive atrophic, H. Other or Benign-Proliferative, I. Other or Benign-Secretory, J. Other or Benign-Menstrual

was incorrectly recorded. The corrected labels post annotation were the labels that were used for training and testing. This means the final numbers of slides of each type may not match the original percentages described above. The distribution of data over train, validation and test sets is shown in table 1.

All slides were then scanned at QEUH and saved as Whole Slide Images (WSIs). The WSIs are hundreds of thousands of pixels in height and width at the highest magnification and are too large to read into memory. Dedicated WSI formats allow access to either small parts of the image at the highest magnification or the whole image at lower magnifications. For this study, slides were scanned using a Phillips Ultra Fast Scanner (UFS) and stored in the iSyntax file format. The most detailed view in the WSI is level 0, or 40x magnification where the length of a side of 1 pixel in the image is 0.25μm. Higher levels represent lower magnifications in a pyramid where each level is a power of 2 smaller than the previous (Figure 1).

Annotation process

The scanned slides were annotated by a mix of experienced biomedical scientists and pathologists from NHS Greater Glasgow and Clyde. The work of the biomedical scientists was reviewed and approved by a pathologist before use. The annotations took place using the QuPath software [11] the isyntax [12] files were converted to OME-Tiff files using a Glencoe software converter [13] prior to annotation. The code utilised for this conversion is publicly available from Zenodo [10].

Annotation endometrial slides is complicated due the structure of the tissue present on the slides. Some of the slides contained a small number of large contiguous pieces of tissue (Fig 3a), where only annotating the malignant areas is straight forward. However, some of the slides contained a very large number of small fragments of tissue (Fig 3b). These slides would require the pathologists to annotate separately many small bits of tissue on slides where nearly all the tissue was malignant. In addition, some slides contained a very large amount of blood or mucus with no diagnostic value (Fig 3c). Therefore, it was decided that annotating blood and mucus either as a separate class or as part of the "other or benign" class would be time prohibitive and an alternative approach was needed. The widely used method for annotating H&E slides takes the approach that only the area of interest is annotated and the rest of tissue is considered as normal tissue and therefore is not annotated. Due to the structures complexity of the endometrial slides mentioned above, it was decided to take a different approach for annotating these slides.

The annotation approach taken for endometrial WSIs gives an overall class to the slide, and then to only annotate parts of the slide that differed from the overall class. The classes used for annotation were "malignant" and "other or benign". Although there are slides categorised as "insufficient", these slides are characterised by a lack of tissue rather than a specific type of tissue, so "insufficient" was not used as an annotation class. Annotators were not required to denote the areas of tissue on the slide as tissue detection was

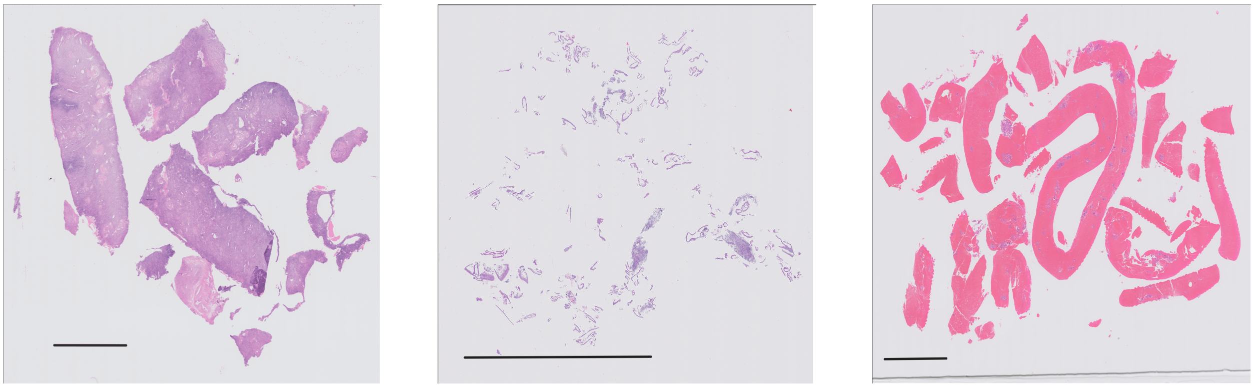

(a) Slide with a few large pieces of tissue. **(b)** Slide with many small fragments of tissue. **(c)** Slide with large amounts of blood clot.

Figure 3. Examples of slides with different amounts and presentation of tissue (Scale bar = 5mm).

applied as part of the pre-processing algorithm. Hence, a large number of the annotation files were blank as everything on the slide was from the overall class with no other annotation required.

Fig 4 shows examples of endometrial slides where all the tissue on the slide is of the overall category assigned to that slide and therefore the annotation files for them are blank as no annotation was needed for them. Tissue detection or background separation and blood and mucus detection are then applied to the slide in preprocessing stage. To detect the tissue and separate it from the background, a thumbnail image of the slide at level 5 is created. Fig 3c shows how multiple tissue areas are saved as separate images in iSyntax format to reduce the file size. In the thumbnails the missing areas between these images are pure black pixels. Any pixels in the thumbnail that are pure black are converted to pure white. The image was then converted to greyscale and as the background is predominately white any values of greater than 0.85 were considered to be background. Next a closing transform and a hole filling morphological operation are applied, the operations improve the amount of tissue captured around edges and holes. The mask created by the tissue detection algorithm for the slide shown in Fig 4a is shown in Fig 4c when when tissue detection is combined with the annotation (Fig 4b) it gives the areas of the slide as "malignant" or "other or benign" tissue as shown in Fig 4d.

The second stage is to identify any blood or mucus on the slide. Blood and mucus detection is carried out on a pixel by pixel basis. Each of the red, green and blue (RGB) channels are considered separately. A Gaussian filter with a kernel size of 2 is applied. Then a texture filter is applied to each channel both with and without the Gaussian filter to give a total of 12 different features for each pixel (raw pixel value, Gaussian filtered value, texture filter on raw, texture filter on Gaussian filter, for each of 3 channels). A random forest model was trained using a small subset of images with detailed annotations to determine the difference between "blood or mucus" and "tissue" pixels. The trained blood and mucus detection model was then applied to each image to identify "blood or mucus". For the slide shown in Fig 5A the areas detected as "blood or mucus" are shown in Fig 5E when this is combined with the tissue detection and annotations it gives the areas of the slide as "malignant" or "other or benign" as shown in Fig 5.

Fig 6 shows examples of slides where different categories are present on the slide. In these examples the background and blood and mucus area are detected later in tissue detection and blood or mucus detection stages.

Inter-observer variability in annotation

Disagreements can occur among pathologists when categorizing slides. To assess this variability, three pathologists independently annotated a subset of 295 test slides. Their agreement was measured using Cohen's kappa statistic, with the arithmetic mean of all observer pairs reported [9]. Despite strong concordance, some inconsistencies were observed. The most frequent category-level disagreements were between "insufficient" and "other benign", while at the subcategory level, differences arose mainly between "insufficient" and "inactive/atrophic", as well as "hyperplasia with atypia" and "adenocarcinoma" within the malignant class. These disagreements are visualized in the confusion matrices [9].

Data Validation and quality control

The images submitted were obtained directly from cases undergoing clinical histopathological diagnosis and were subject to rigorous scrutiny by the specialist team of diagnostic histopathologists who undertook the manual annotations of selected features. The annotations were added afterwards as a separate exercise, not linked to clinical diagnosis. The gold standard was the pathologists' diagnosis and where there was discrepancies, by consensus review.

Using H&E endometrial WSI dataset and their annotations, ML algorithms can be applied to assist in various aspects of cervical health analysis. Data collection and preprocessing is the first step in illustrating how ML algorithms can utilise this data.

Extracting nuclear morphological features using Indica Halo AI

WSI images were imported into Indica HALO and HALO AI (v.3.6.4134), along with corresponding annotation files created in QuPath by pathologists. A nuclei segmentation classifier, underpinned by advanced deep learning neural network algorithms, was trained with examples from multiple different cases 7. An analysis algorithm, Multiplex IHC v.3.2.3 was utilised to segment individual nuclei to extract nuclear morphological features such as area, perimeter, and roundness within the annotation (Figure 5). The tabular data from the individual nuclear morphological features, along with their x and y coordinates, was exported into CSV file format.

Re-use potential

The endometrium can display a wide range of histological appearances with overlapping features which makes the diagnosis of various lesions complex and specifically distinguishing between premalignant and malignant conditions challenging. The diverse presentation of symptoms of endometrial abnormalities may be attributed to different underlying conditions, making accurate diagnosis based solely on clinical presentation impossible. This dataset includes a wide range of endometrial whole slide images containing a wide spectrum of histological conditions that have been annotated by pathologists and can be used for training AI based algorithms to identify the endometrial abnormalities and to detect slides with

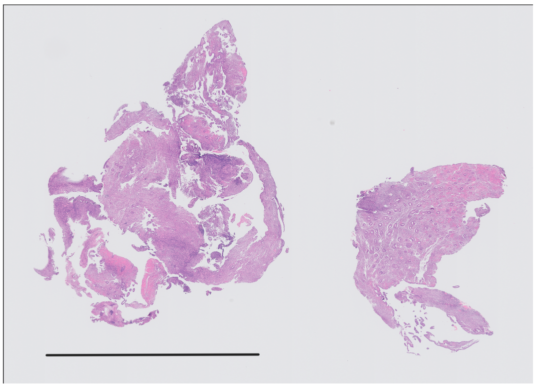

(a) Malignant slide

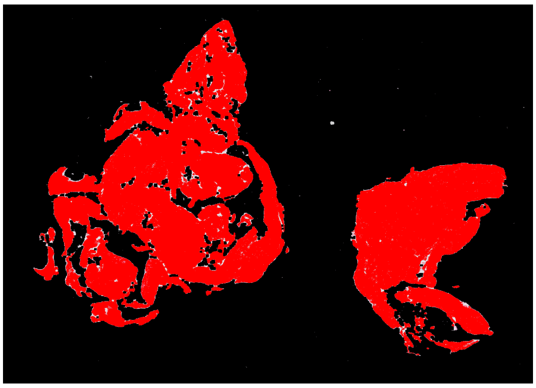

(b) Overlayed annotations on the malignant slide

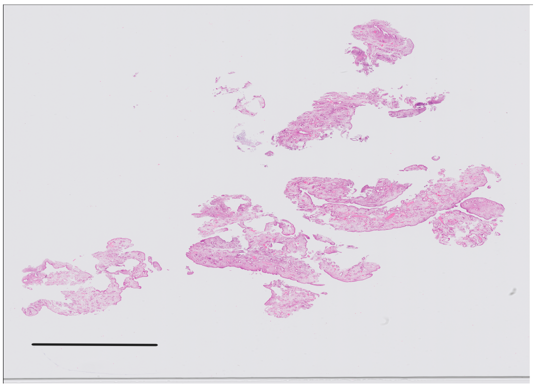

(c) Normal slide

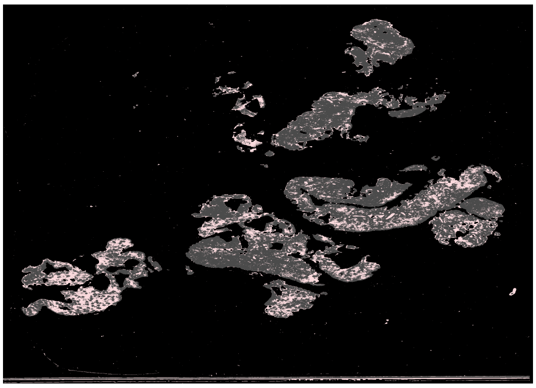

(d) Overlayed annotations on the normal slide

Figure 4. Examples of slides where all tissue on the slide is of the same category. (Scale bar = 5mm)

Malignant, blood or mucus, Normal Tissue, Background

malignant tissue to allow prioritisation of these slides in a queue for pathologist review and hence reduce time to diagnosis for patients with cancer. Moreover, nuclear morphological features such as area, perimeter, and roundness may enhance accuracy in distinguishing between pre-malignant and malignant. Furthermore, one problem in diagnostic pathology is how to deal with small samples that may not be representative nor provide conclusive evidence for the pathologist. On occasion this may necessitate a report that states "tissue insufficient for diagnosis", which in turn may lead to a further sample being sought. This is a potential distressing and painful procedure for the patient. Being more confident about defining an inadequate sample will help workflow, ensure safety and minimise unnecessary discomfort to the patient.

This dataset was utilised to generate three models distinguishing three-class classification , "malignant," "other or benign," and "insufficient" whole slide images (WSIs) [9]. Among these, the CNN achieved the highest accuracy in identifying malignant cases, with classification accuracy ranging from 89.8% to 92.1% depending on whether any tissue patches or majority-tissue patches were used. However, its overall accuracy (85.2%–90.8%) was slightly lower than that of the Random Forest model, which yielded the highest overall accuracy but underperformed in correctly identifying malignant cases. XGBoost provided a balanced performance, intermediate between the CNN and Random Forest classifiers. These results, presented in detail in the original publication [9] , offer a benchmark for future method development and validation.

Ethical Approval

- Ethics approval for the study was granted by NHS Greater Glasgow and Clyde Biorepository and Pathology Tissue Resource (REC reference 16/WS/0207) on 4th April 2019.
- Biorepository approval was obtained (application number 511)
- Local approval was obtained from the School of Computer Science Ethics Committee, acting on behalf of the University Teaching and Research Ethics Committee (UTREC) [Approval code-CS15840].

Data availability

All endometrial whole slide images, their annotation files, binary masks and a metadata file (2909 images in iSyntax format, 2909 annotation files in JSON format, 2909 binary masks in PNG format and a metadata file in CSV format) and the morphological features extracted from them in Halo are openly available in the GigaScience repository, GigaDB [S-BIAD1199] [14].

Declarations

List of abbreviations

- Artificial Intelligence (AI)
- Machine Learning (ML)
- Whole Slide Image (WSI)
- Convolutional Neural Network (CNN)
- Clinical Proteomic Tumor Analysis Consortium (CPTAC)
- Teta Bytes (TB)
- Industrial Centre for Artificial Intelligence Research in Digital Diagnostics (iCAIRD)
- Quantitative Pathology (QuPath)

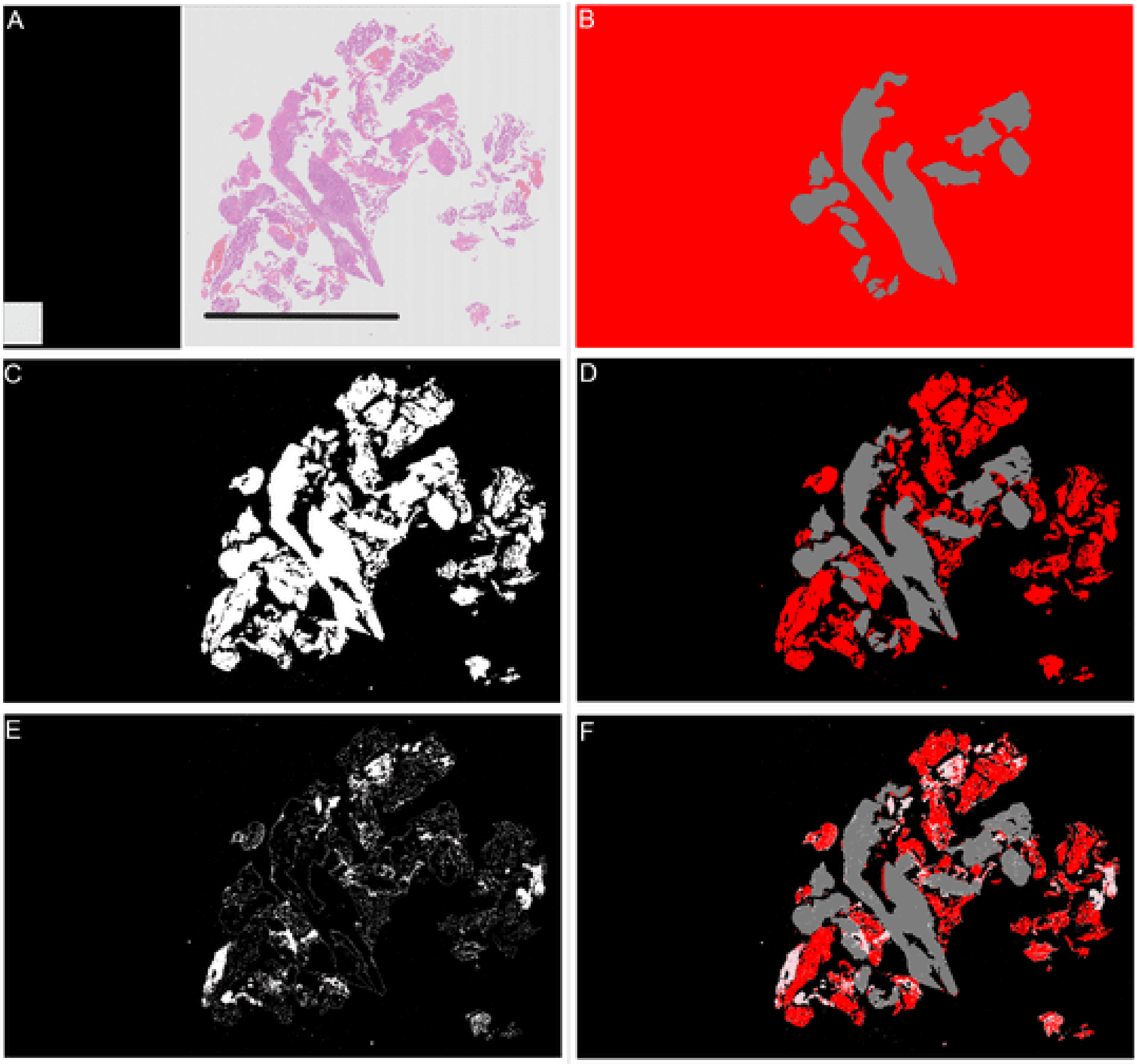

Figure 5. Examples of all stages in slide annotation and detection of tissue. (Scale bar = 5mm)
(A) Thumbnail of "malignant" slide where some tissue is "other or benign". (B) Annotation for "malignant" slide where some tissue is "other or benign". (C) Mask showing areas detected as tissue in white, background is shown in black. (D) Combined annotation and tissue detection. (E) Calculated mask showing areas detected as "blood or mucus" in white, anything that is not "blood or mucus" is shown as black. (F) Combined annotation, tissue and "blood or mucus" detection.
Malignant blood or mucus Normal Tissue Background

Consent for publication

Not applicable.

Competing Interests

The authors declare that they have no competing interests.

Funding

This work is supported by the Industrial Centre for AI Research in digital Diagnostics (iCAIRD) which is funded by Innovate UK on behalf of UK Research and Innovation (UKRI) [project number: 104690], and in part by Chief Scientist Office, Scotland.

Author's Contributions

Mahnaz Mohammadi wrote the manuscript and supervised data preprocessing, together with Christina Fell and In Hwa Um.
Prishma Shahi imported annotations in Indica Halo AI platform and measured nuclear morphological features.
Gareth Bryson initiated the project, and Sarah Bell, Sheeba Syed, and Prakash Konanahalli annotated the whole slide images. David Harris Birtill and Ognjen Arandjelovic supervised machine learning experiments.
Clare Orange arranged data release from Glasgow Biorepository. James Blackwood oversaw governance procedures, established digital pathology services and supervised data de-identification and release. David Harrison is Director of iCAIRD, obtained funding, reviewed results and helped to draft the manuscript. All authors have seen and approved the manuscript.

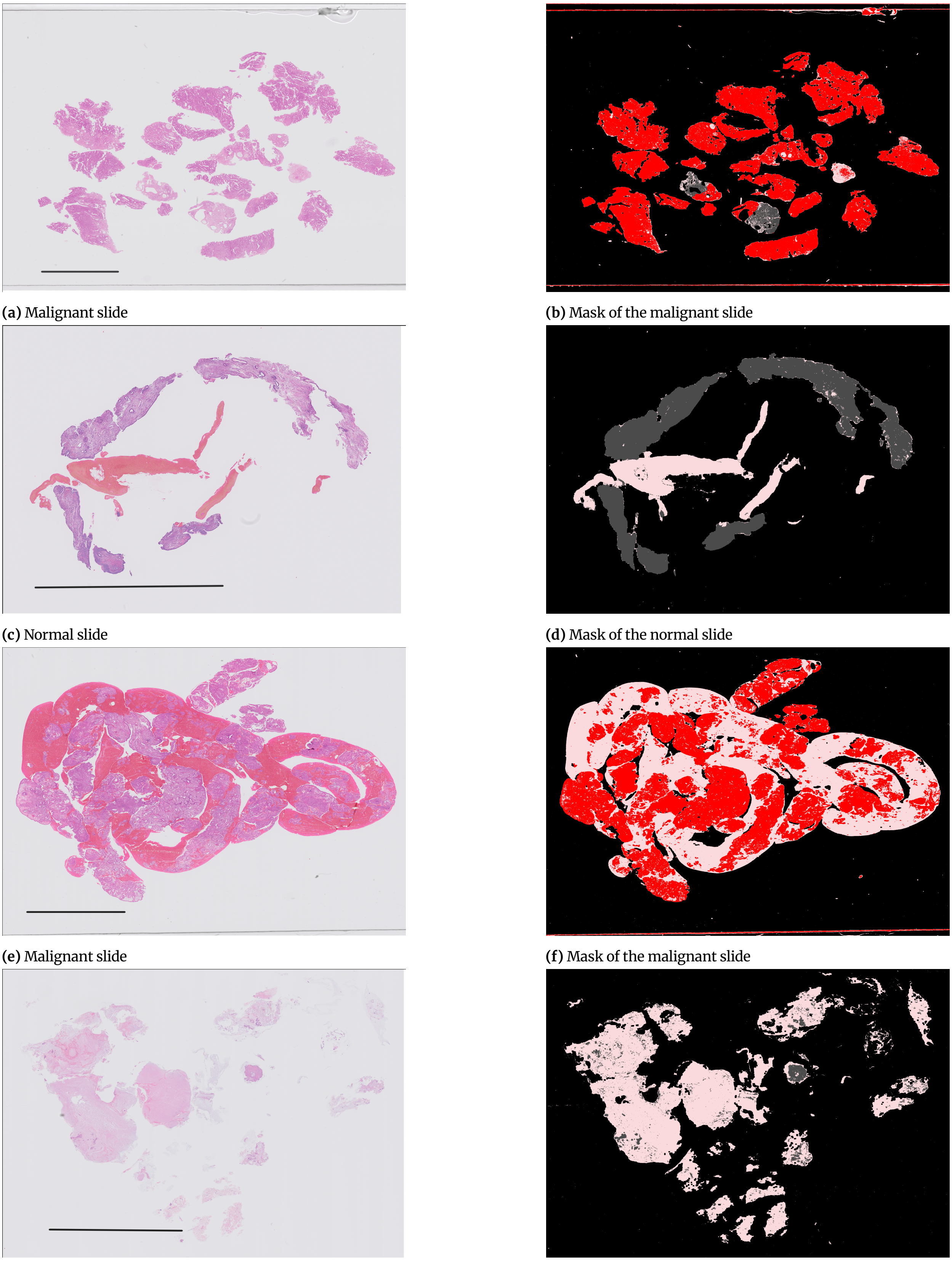

(a) Malignant slide
(b) Mask of the malignant slide
(c) Normal slide
(d) Mask of the normal slide
(e) Malignant slide
(f) Mask of the malignant slide
(g) Insufficient slide
(h) Mask of the insufficient slide

Figure 6. Examples of slides and their masks after applying overlaying annotations and applying tissue and blood or mucus detection stages to the slide. (Scale bar = 5mm)

Malignant, blood or mucus, Normal Tissue, Background

Acknowledgements

We acknowledge the support of NHS Research Scotland (NRS) Greater Glasgow and Clyde Biorepository. We acknowledge the sup-

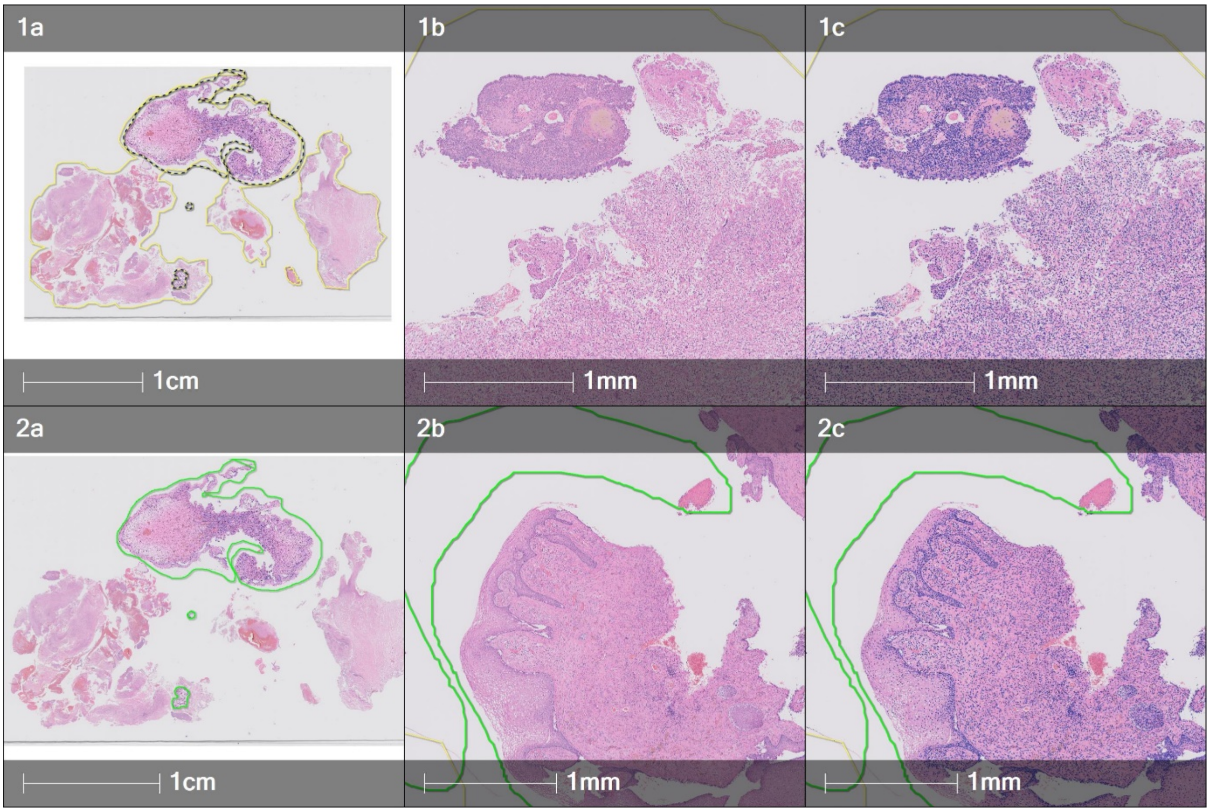

Figure 7. Example of segmented nuclei (colored blue) in two different annotations in the same patient using Indica HALO AI platform
(1a) Annotation of the malignant area (yellow line), having excluded the normal cervix (dotted yellow annotation). (2a) Annotation of normal cervix (green line). (1b, 2b) Higher magnification of 1a and 2a, respectively. (1c, 2c) Multiplex IHC analysis algorithm was used to segment individual nuclei (Blue nuclei mask) and to extract their morphological features within annotations.

port of the biomedical scientists, Tim Prosser, Lucy Irving, Jennifer Campbell and Jennifer Faulkner, from the Pathology Department, NHS Greater Glasgow and Clyde for technical support.

Authors' information

MM and CF hold a PhD degree and are data scientists in the School of Medicine, University of St Andrews.
PS is a research technician at the School of Medicine, University of St Andrews.
GB is a Consultant Pathologist and Clinical Director for Laboratory Medicine at the Queen Elizabeth University Hospital, Glasgow. SB, PK and SS are consultant gynaecological pathologists at Queen Elizabeth University Hospital, NHS Greater Glasgow and Clyde, UK. OA and DHB are Reader and Senior Lecturer respectively in Computer Science, University of St Andrews.
IHU is a postdoctoral research fellow in pathology AI in the University of St Andrews. CO is Biorepository Manager in NHS Greater Glasgow and Clyde, and a doctoral candidate in the University of St Andrews.
JDB is Chief Technical Officer of iCAIRD and Innovation Fellow in University of St Andrews. DJH is Professor of Pathology at the University of St Andrews, and Director of iCAIRD.

References

1. Gynaecological Cancer AI.;. https://icaird.com/wp9-gynaecological-cancers/.
2. Akazawa M, Hashimoto K. Artificial intelligence in gynecologic cancers: Current status and future challenges–A systematic review. Artificial Intelligence in Medicine 2021;120:102164.
3. Sun H, Zeng X, Xu T, Peng G, Ma Y. Computer-aided diagnosis in histopathological images of the endometrium using a convolutional neural network and attention mechanisms. IEEE journal of biomedical and health informatics 2019;24(6):1664–1676.
4. Consortium NCICPTA, et al. Radiology data from the clinical proteomic tumor analysis consortium lung squamous cell carcinoma [cptac-lscc] collection [data set]. Cancer Imaging Archive 2018;.
5. Hong R, Liu W, DeLair D, Razavian N, Fenyö D. Predicting endometrial cancer subtypes and molecular features from histopathology images using multi-resolution deep learning models. Cell Reports Medicine 2021;2(9).
6. Wang T, Lu W, Yang F, Liu L, Dong Z, Tang W, et al. Microsatellite instability prediction of uterine corpus endometrial carcinoma based on H&E histology whole-slide imaging. In: 2020 IEEE 17th international symposium on biomedical imaging (ISBI) IEEE; 2020. p. 1289–1292.
7. Fremond S, Andani S, Koelzer VH, et al. Interpretable deep learning predicts the molecular endometrial cancer classification from H&E images: a combined analysis of the PORTEC randomized clinical trials. SSRN 2022;(4144537).
8. Mohammadi M, Cooper J, Arandelović O, Fell C, Morrison D, Syed S, et al. Weakly supervised learning and interpretability for endometrial whole slide image diagnosis. Experimental Biology and Medicine 2022;247(22):2025–2037.
9. Fell C, Mohammadi M, Morrison D, Arandjelović O, Syed S, Konanahalli P, et al. Detection of malignancy in whole slide images of endometrial cancer biopsies using artificial intelligence. Plos one 2023;18(3):e0282577.
10. Fell C, Mohammadi M, Morrison D. StAndrewsMedTech/icairdpath-public: Release for pub-

lication 2023 Feburary;https://zenodo.org/record/7674764.

11. Bankhead P, Loughrey MB, Fernández JA, Dombrowski Y, McArt DG, Dunne PD, et al. QuPath: Open source software for digital pathology image analysis. Scientific reports 2017;7(1):1–7.
12. Hulsken DB, iSyntax – your format for pathology images; 2016. https://www.openpathology.philips.com/isyntax.
13. Mellisa Linkert, Chris Allan, Converting Whole Slide Images to OME-TIFF: A New Workflow; 2019. https://www.glencoesoftware.com/blog/2019/12/09/converting-whole-slide-images-to-OME-TIFF.html, Last accessed on 2022-08-12.
14. In Hwa Um CFDMSBGBSSPKCOPSDHDHB Mahnaz Mohammadi, Arandelovic O, Endometrial Whole Slide Images Dataset; 2024. https://www.ebi.ac.uk/biostudies/bioimages/studies/S-BIAD1199.

Click here to access/download;Figure;Fig1.png

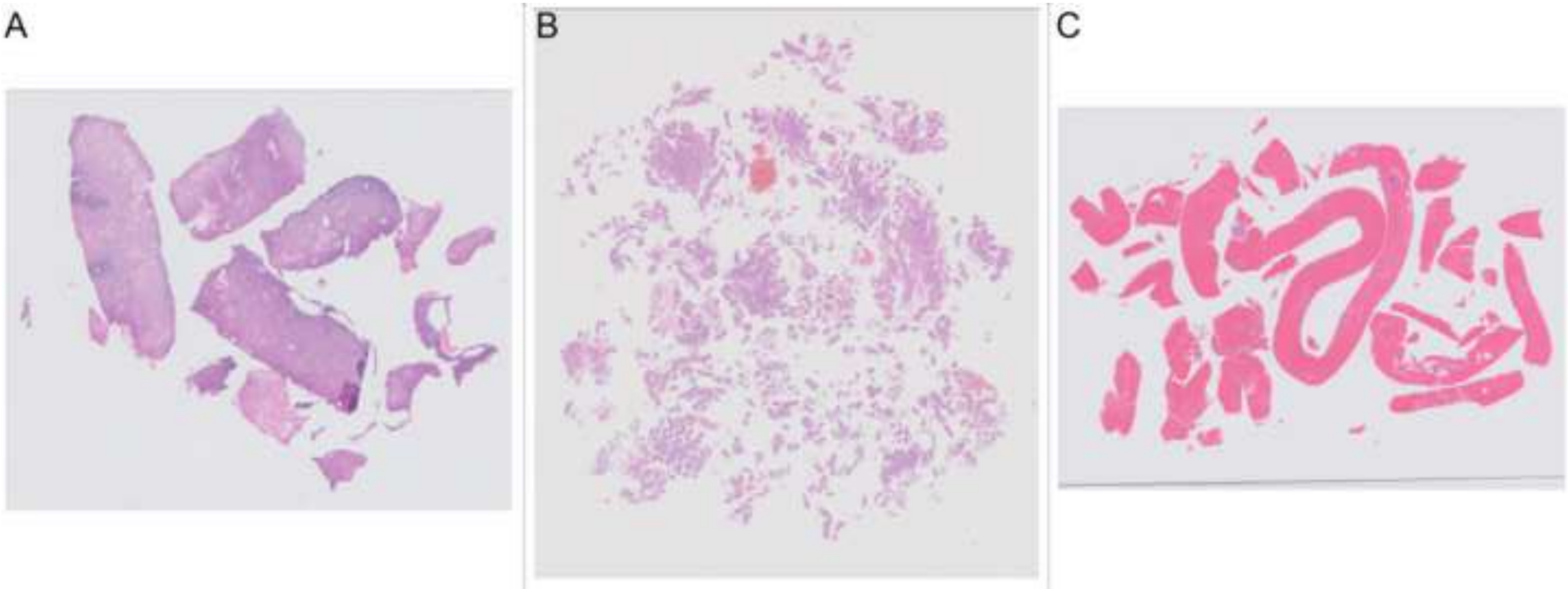

Click here to access/download;Figure;halo_endo_features.jpg

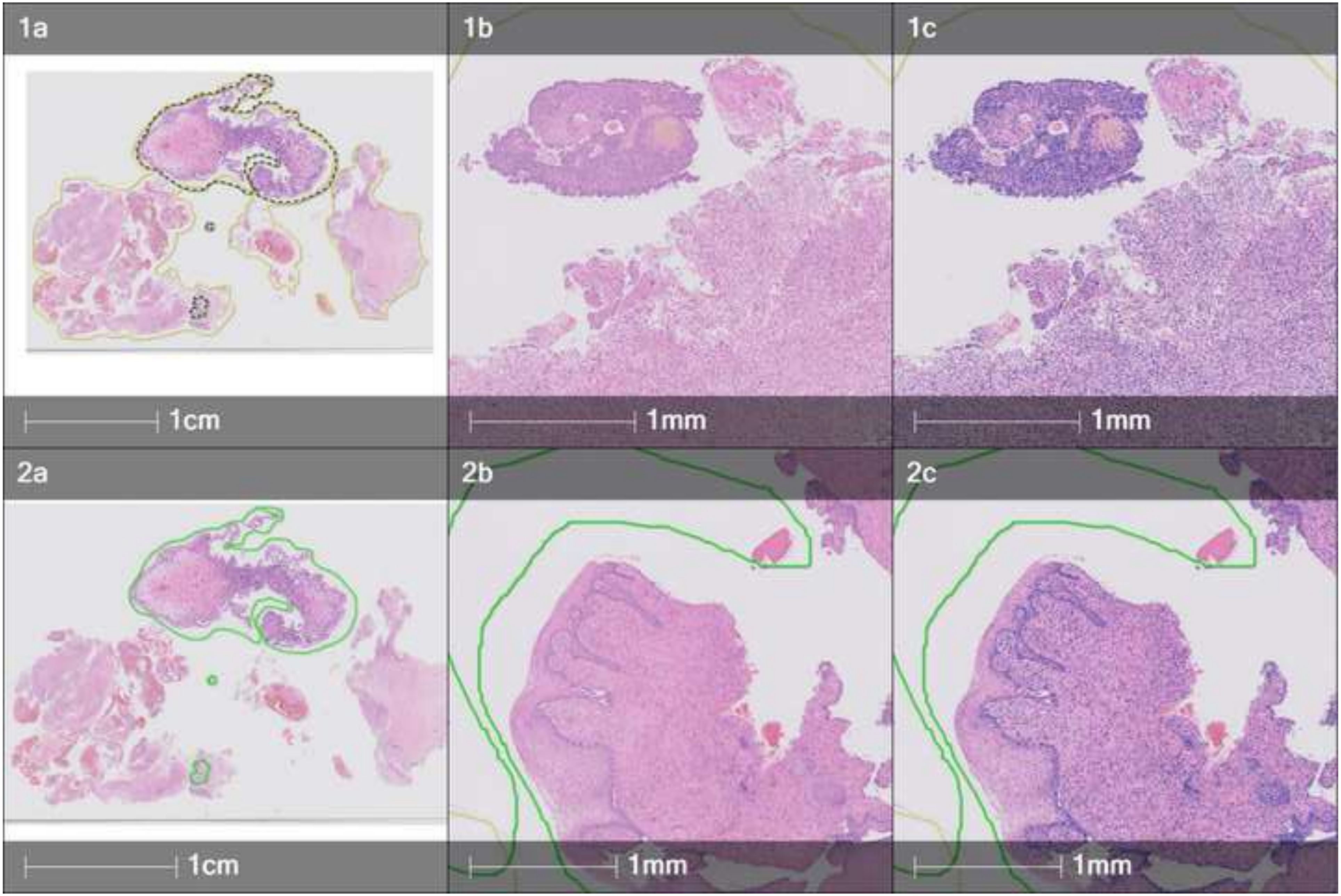

Click here to access/download;Figure;IC-EN-00020-01_mask.png

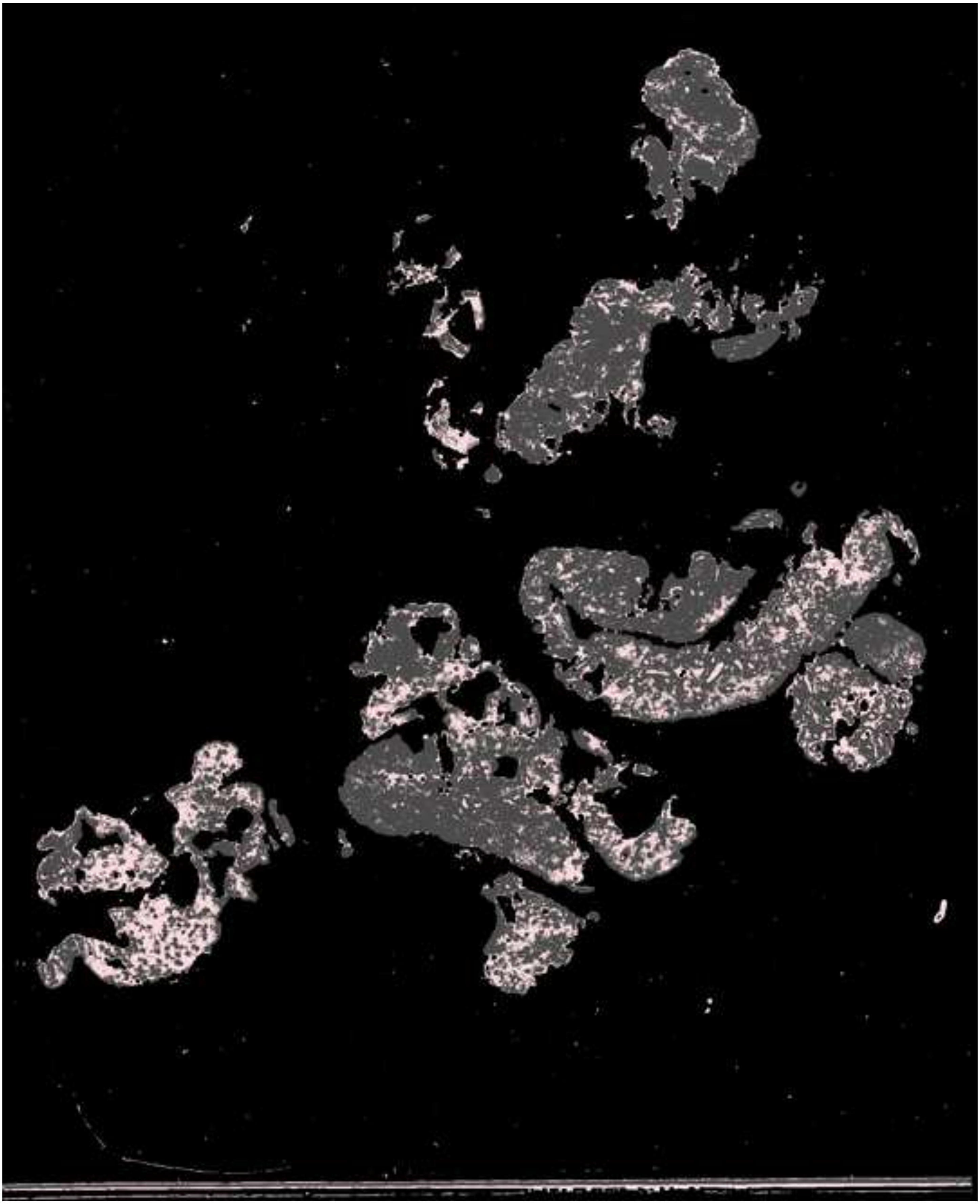

Click here to access/download;Figure;IC-EN-00027-01_mask.png

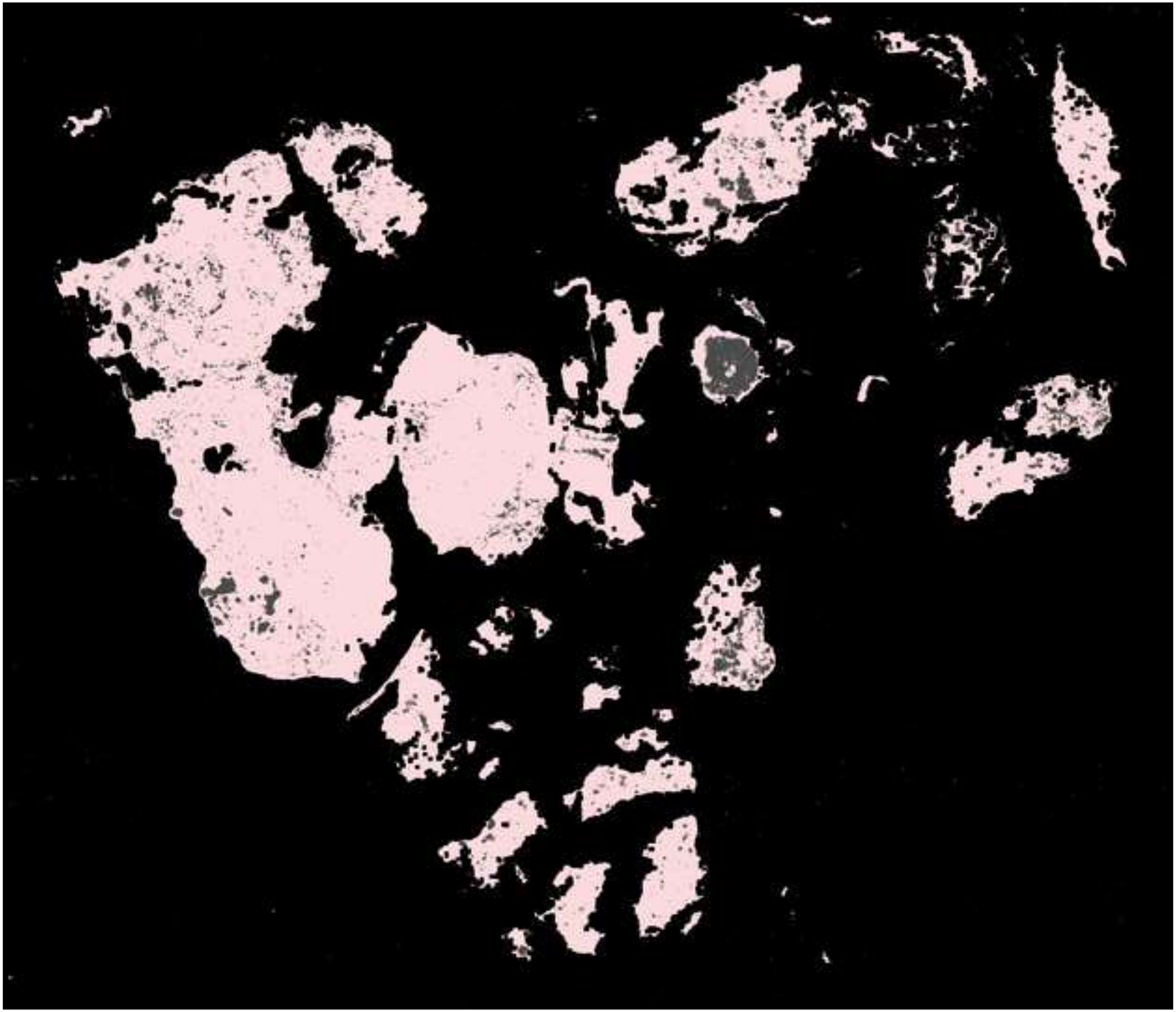

Click here to access/download;Figure;IC-EN-00029-01_mask.png

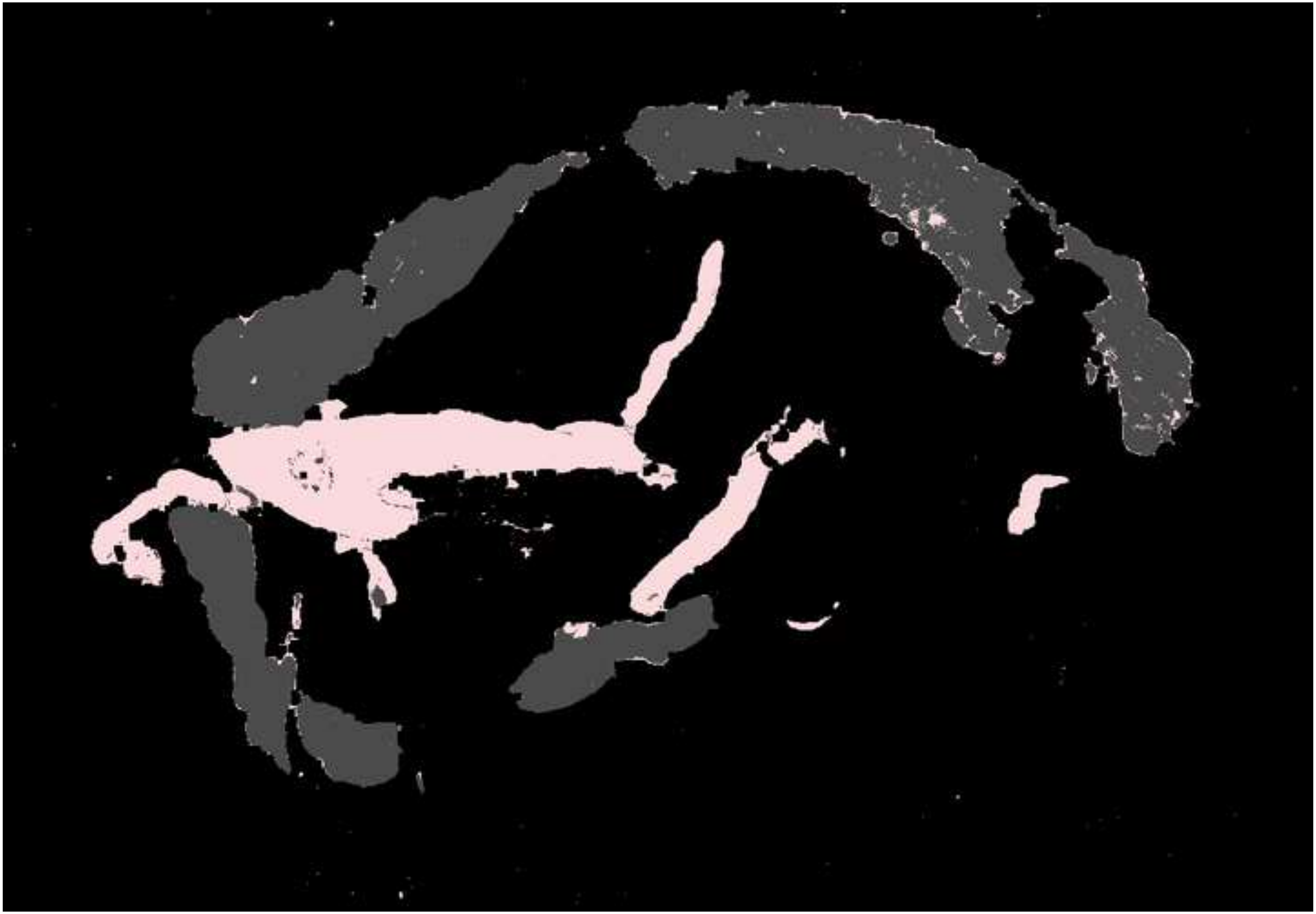

Click here to access/download;Figure;IC-EN-00033-01_mask.png

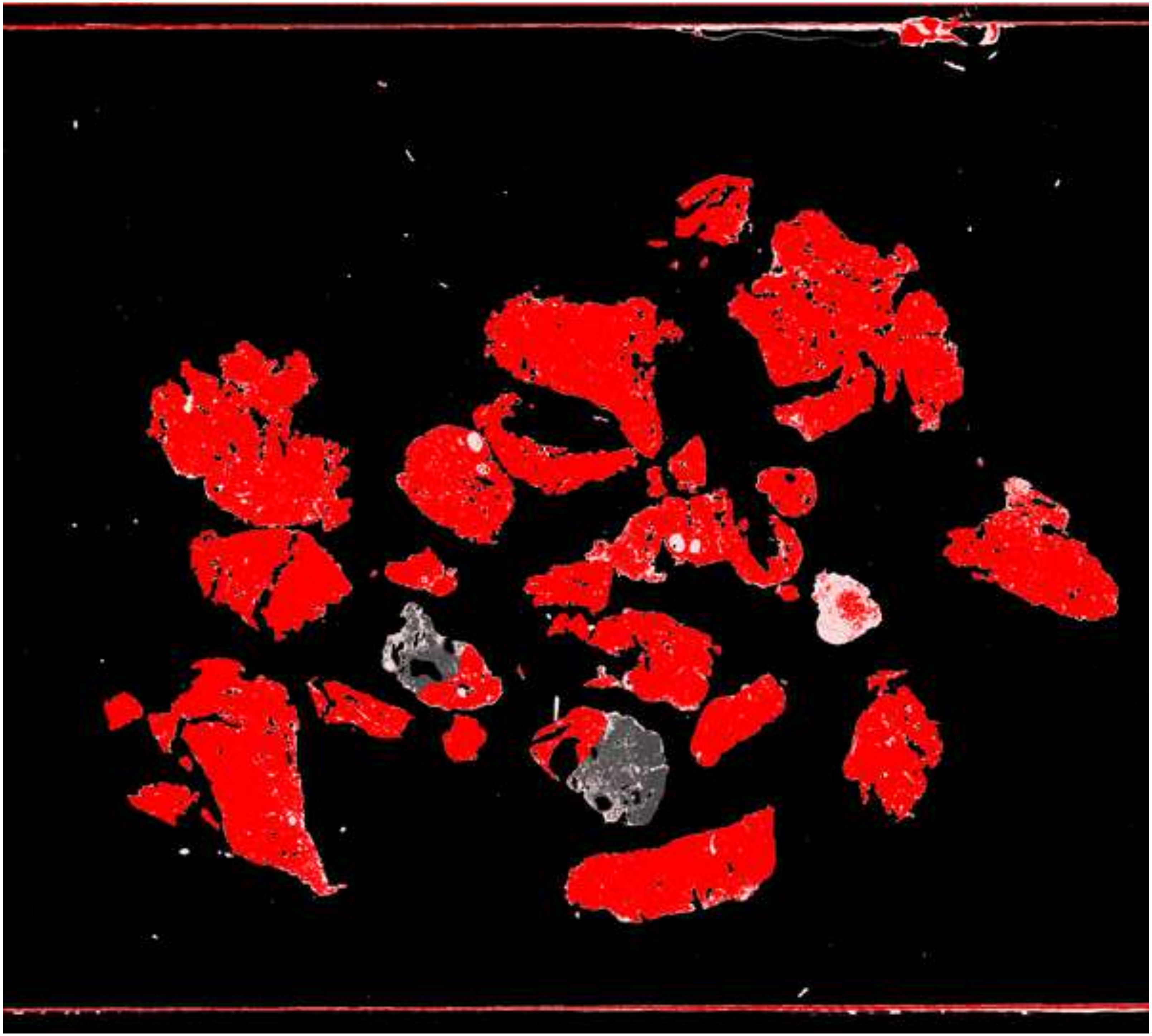

Click here to access/download;Figure;IC-EN-00040-01_mask.png

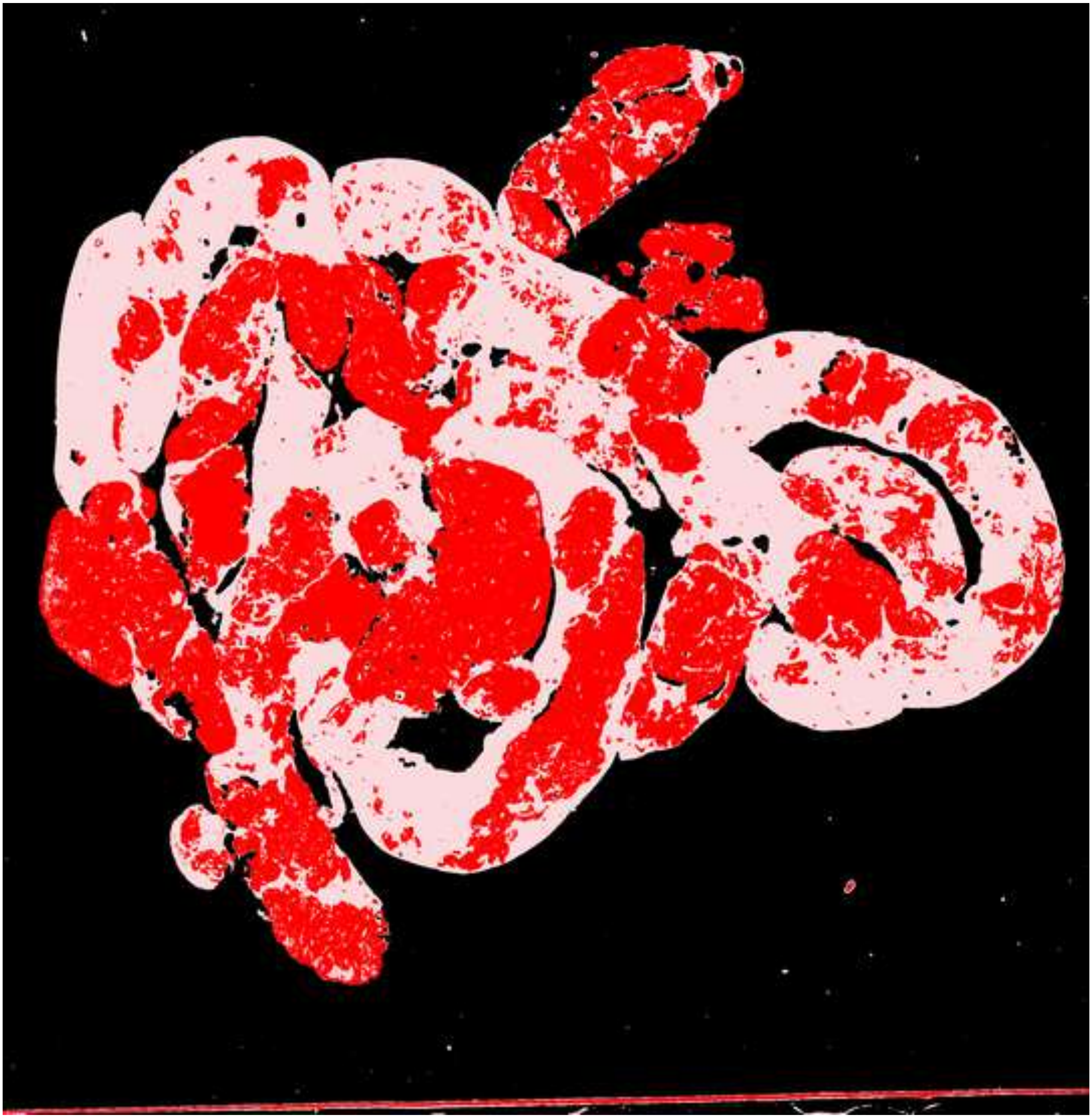

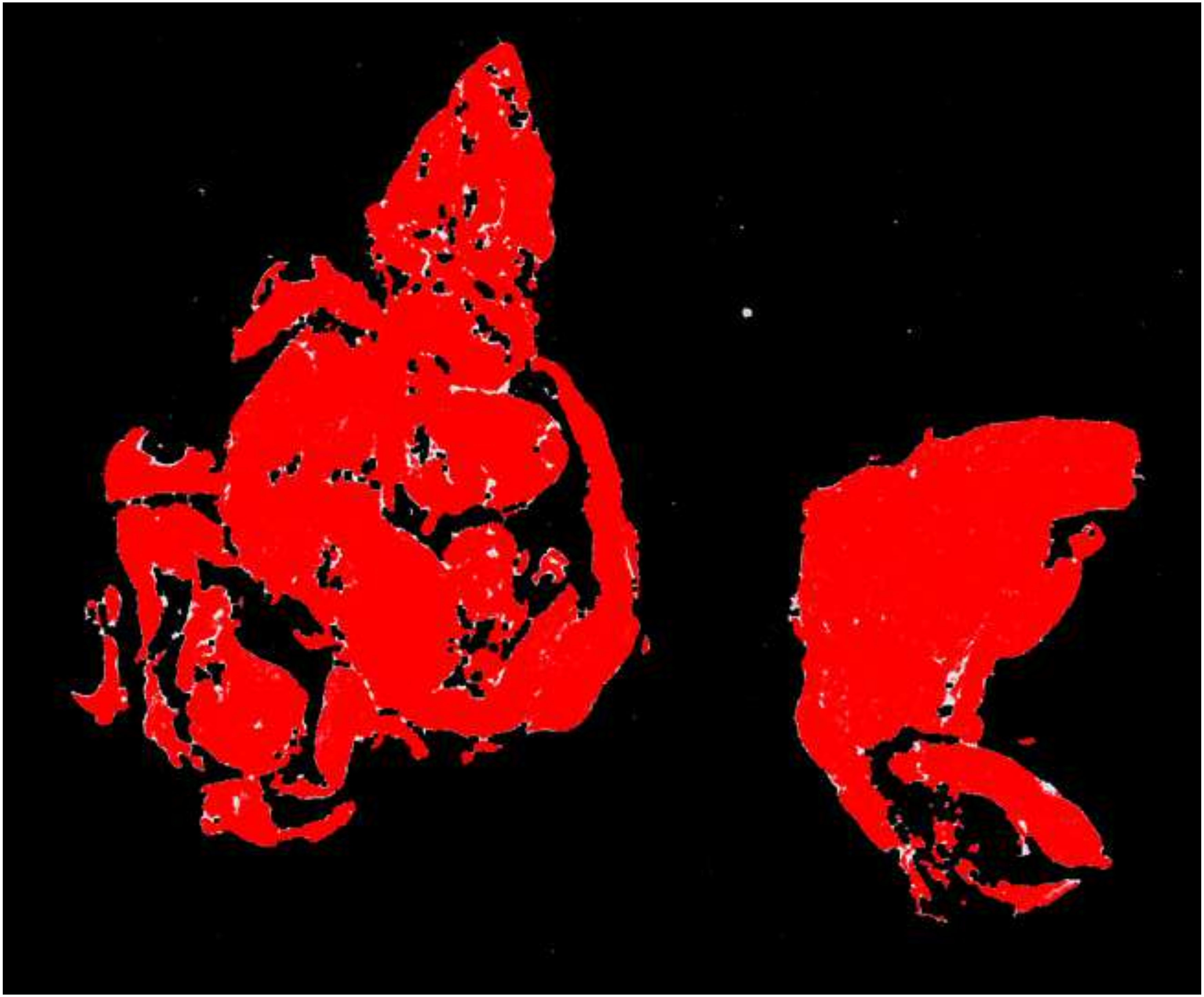

Click here to access/download;Figure;endometrial_subcategorry-smaller7.jpg

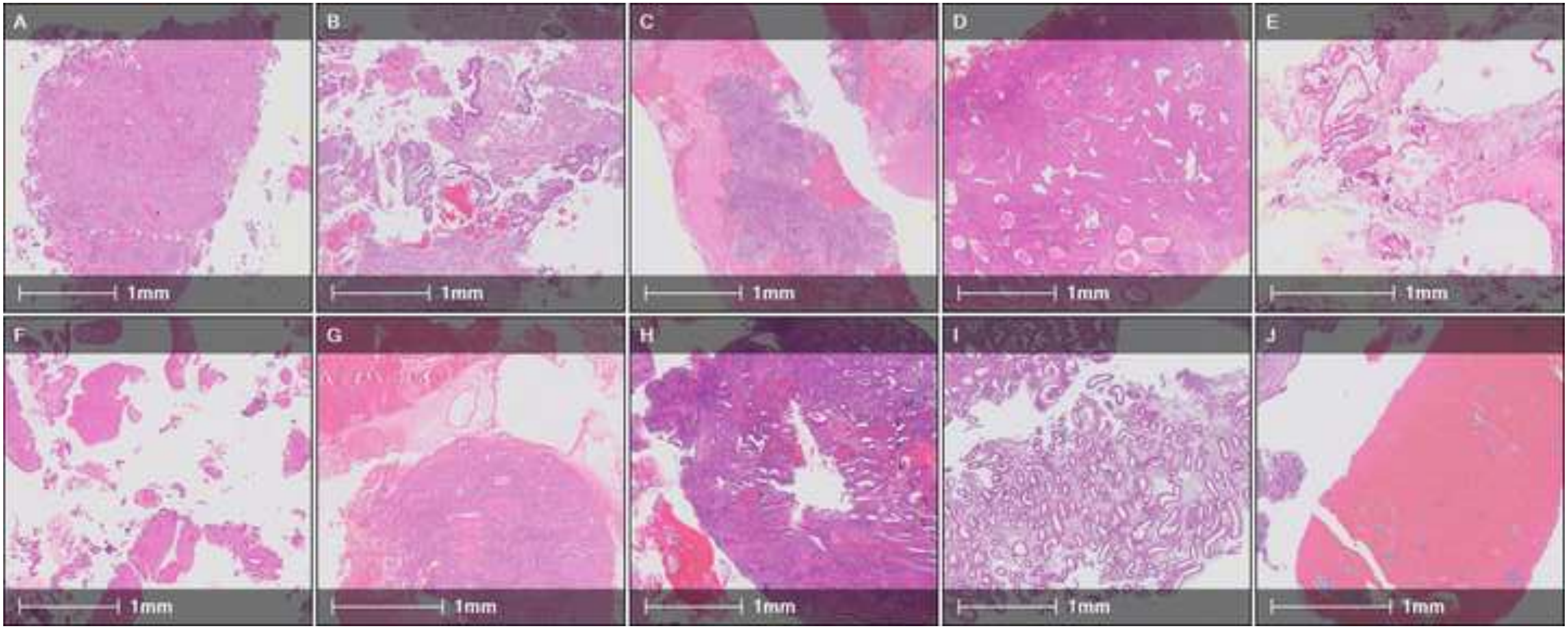

Click here to access/download;Figure;Fig3.png

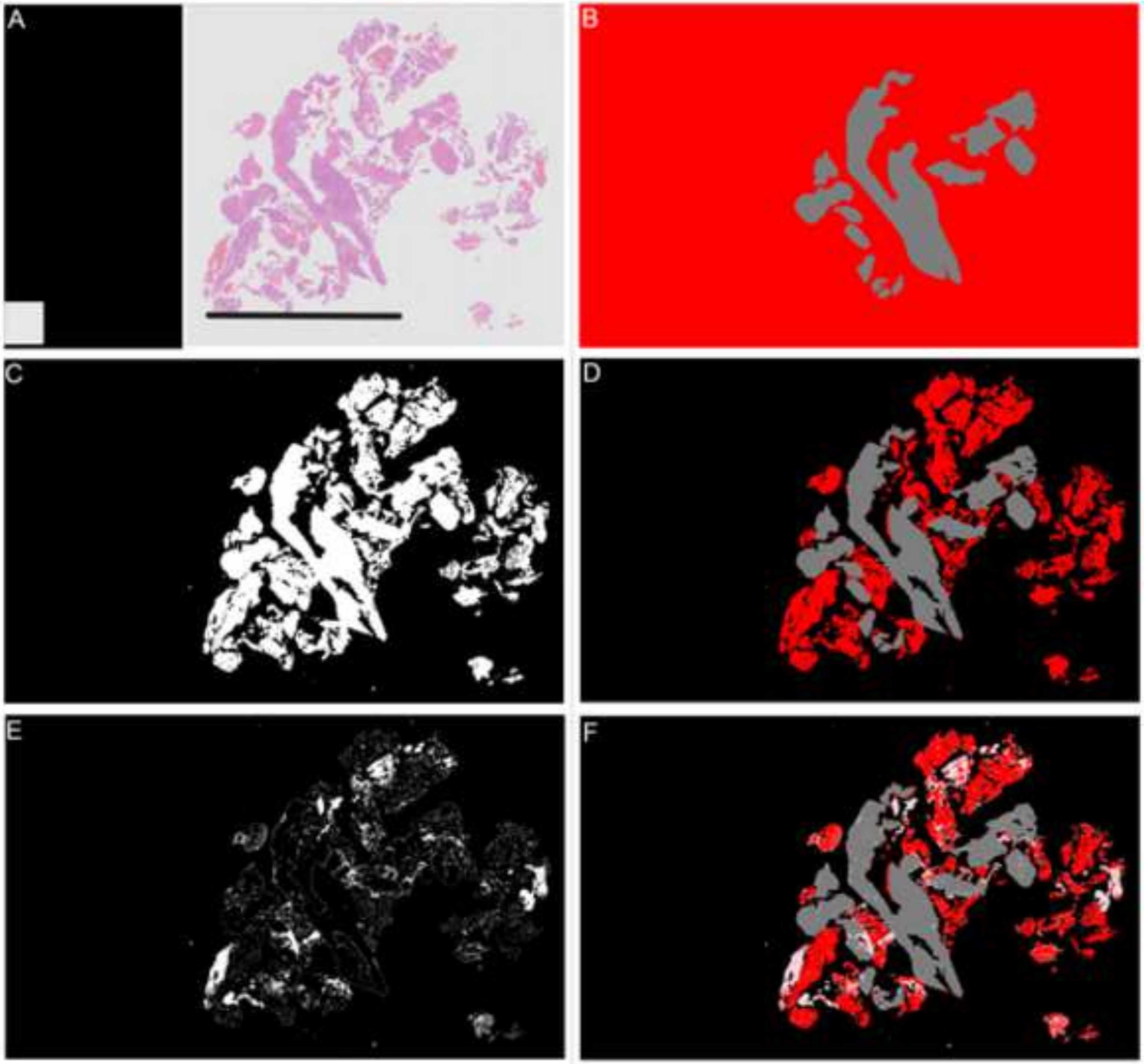

Click here to access/download;Figure;IC-EN-00020-01_thumbnail.png

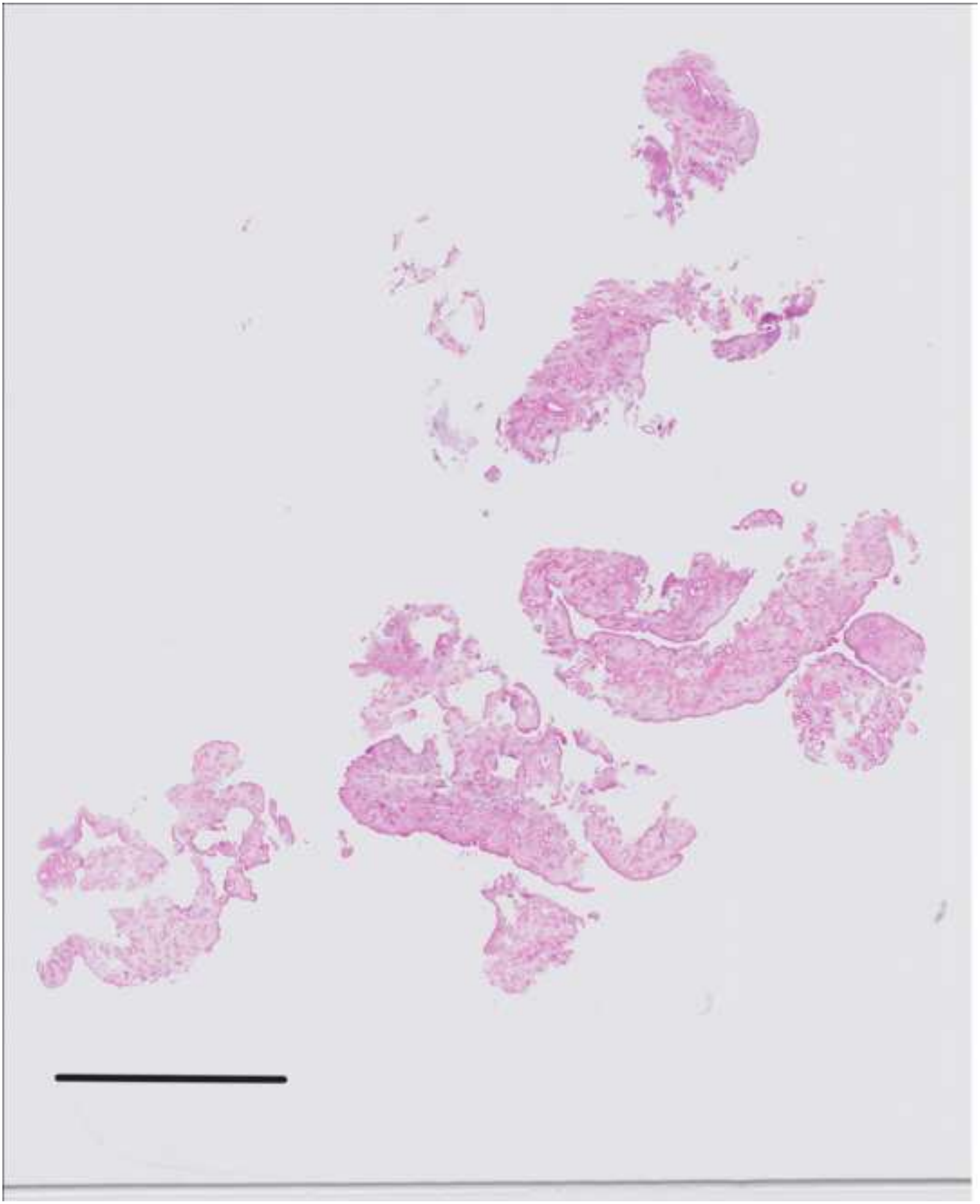

Click here to access/download;Figure;IC-EN-00027-01_thumbnail.png

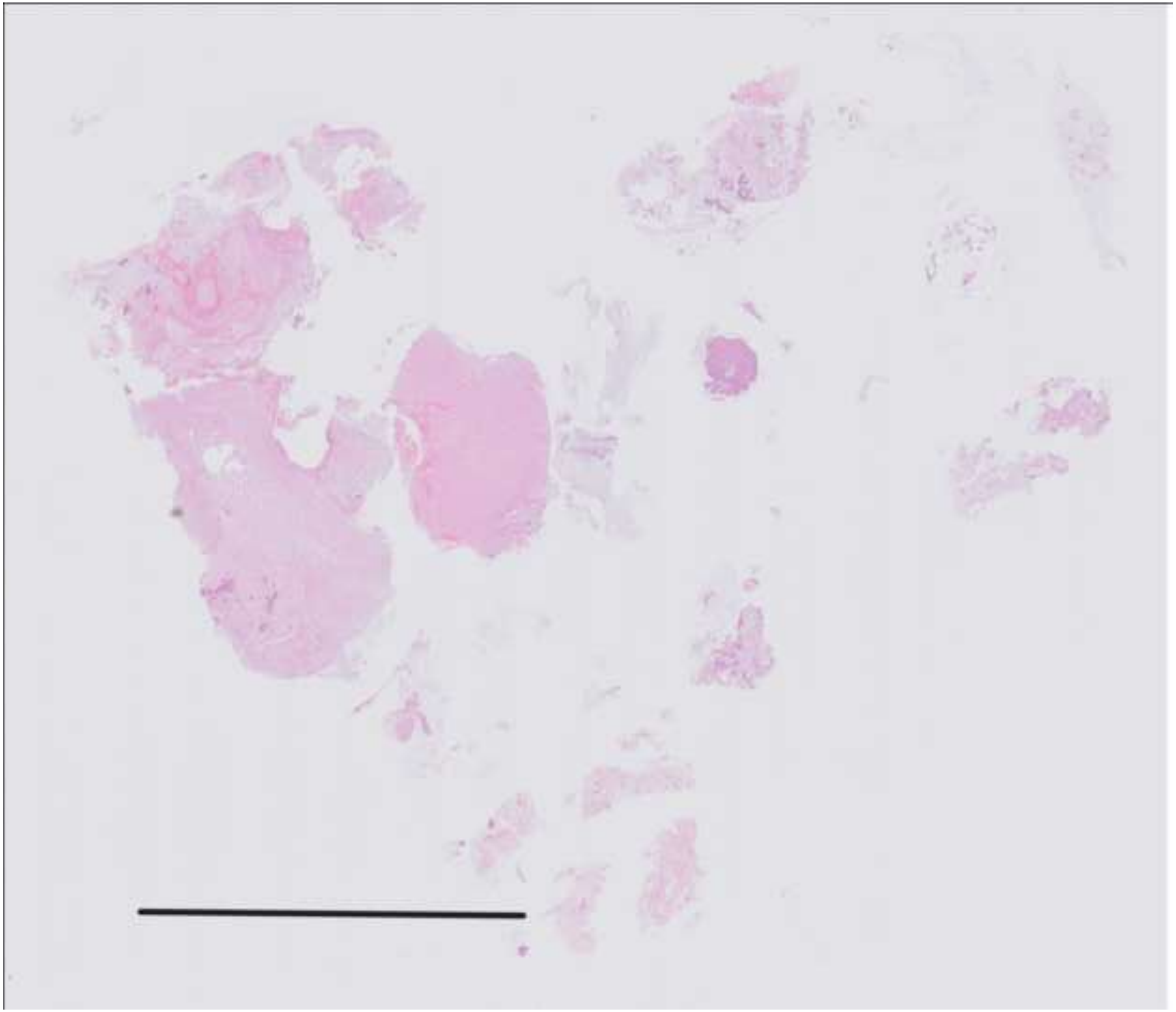

Click here to access/download;Figure;IC-EN-00029-01_thumbnail.png

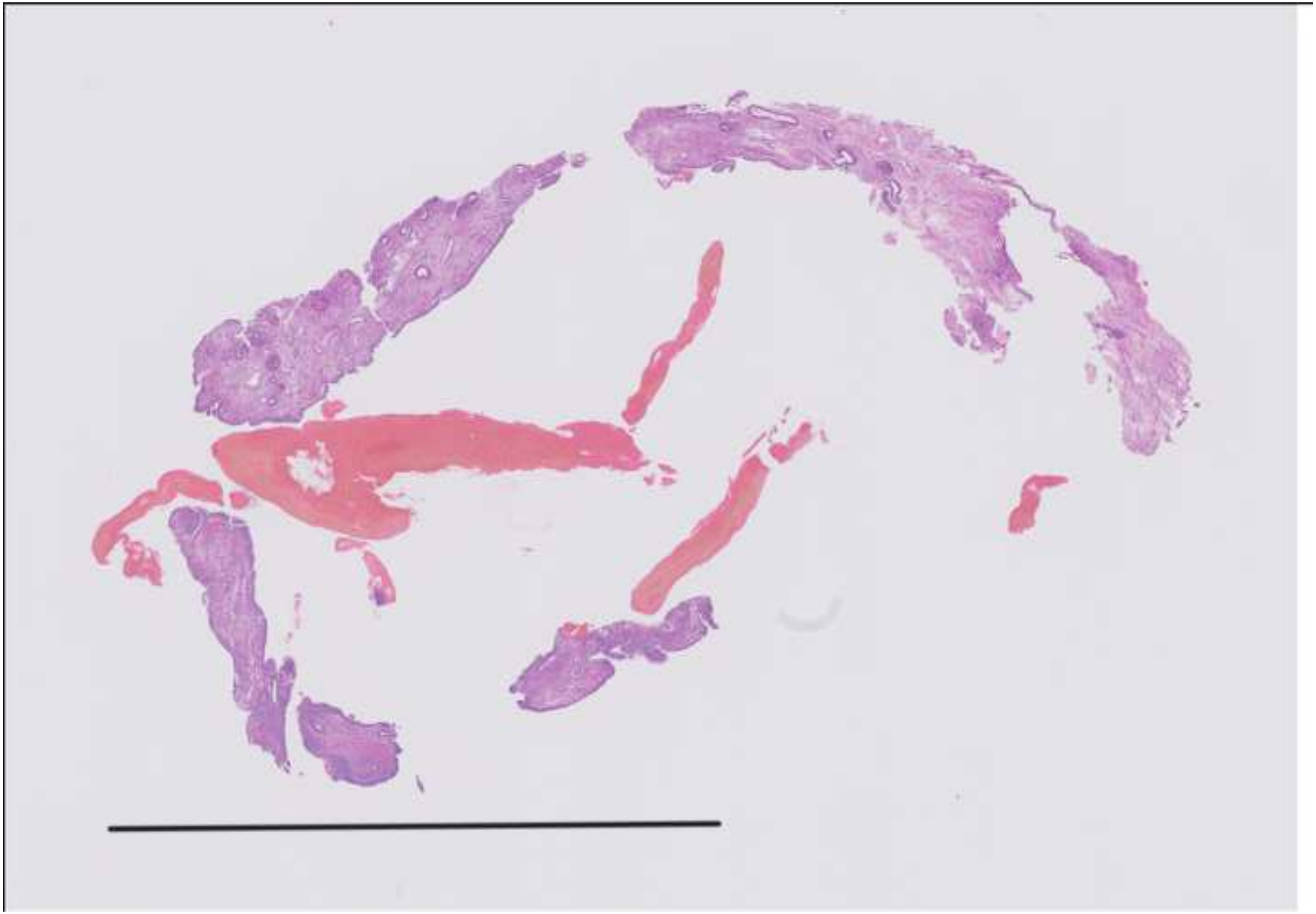

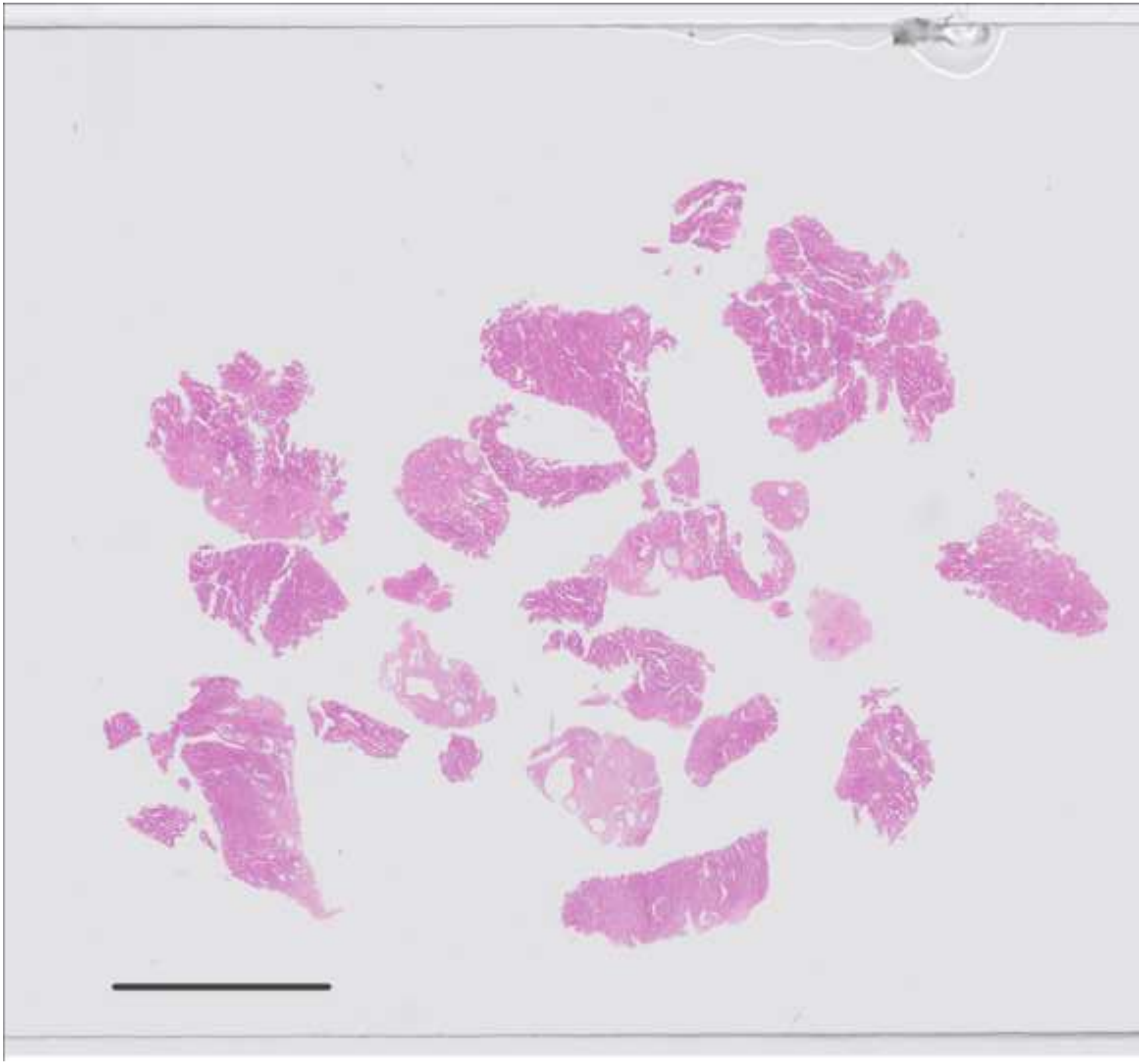

Click here to access/download;Figure;IC-EN-00040-01_thumbnail.png

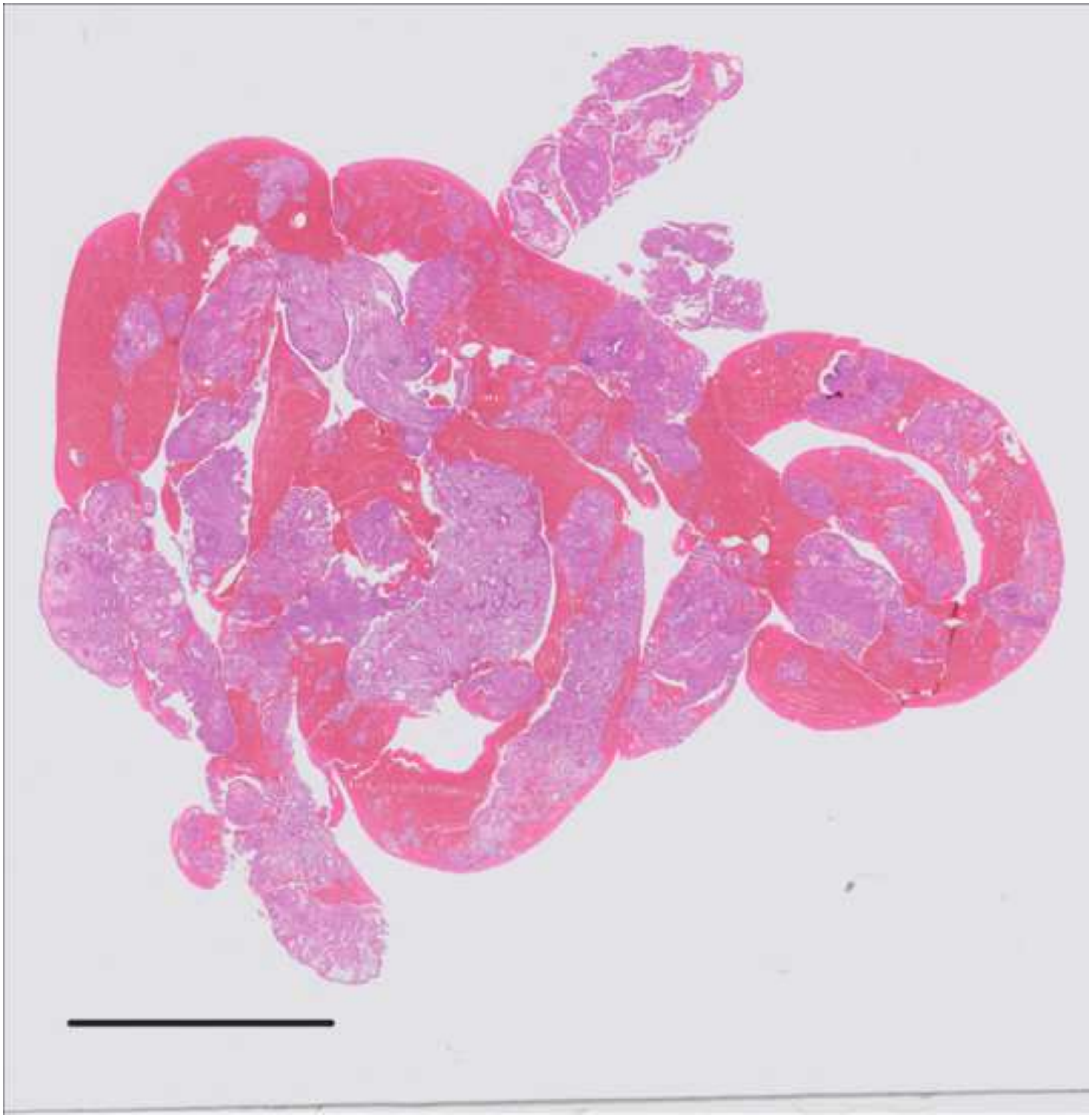

Click here to access/download;Figure;IC-EN-00079-01_thumbnail.png

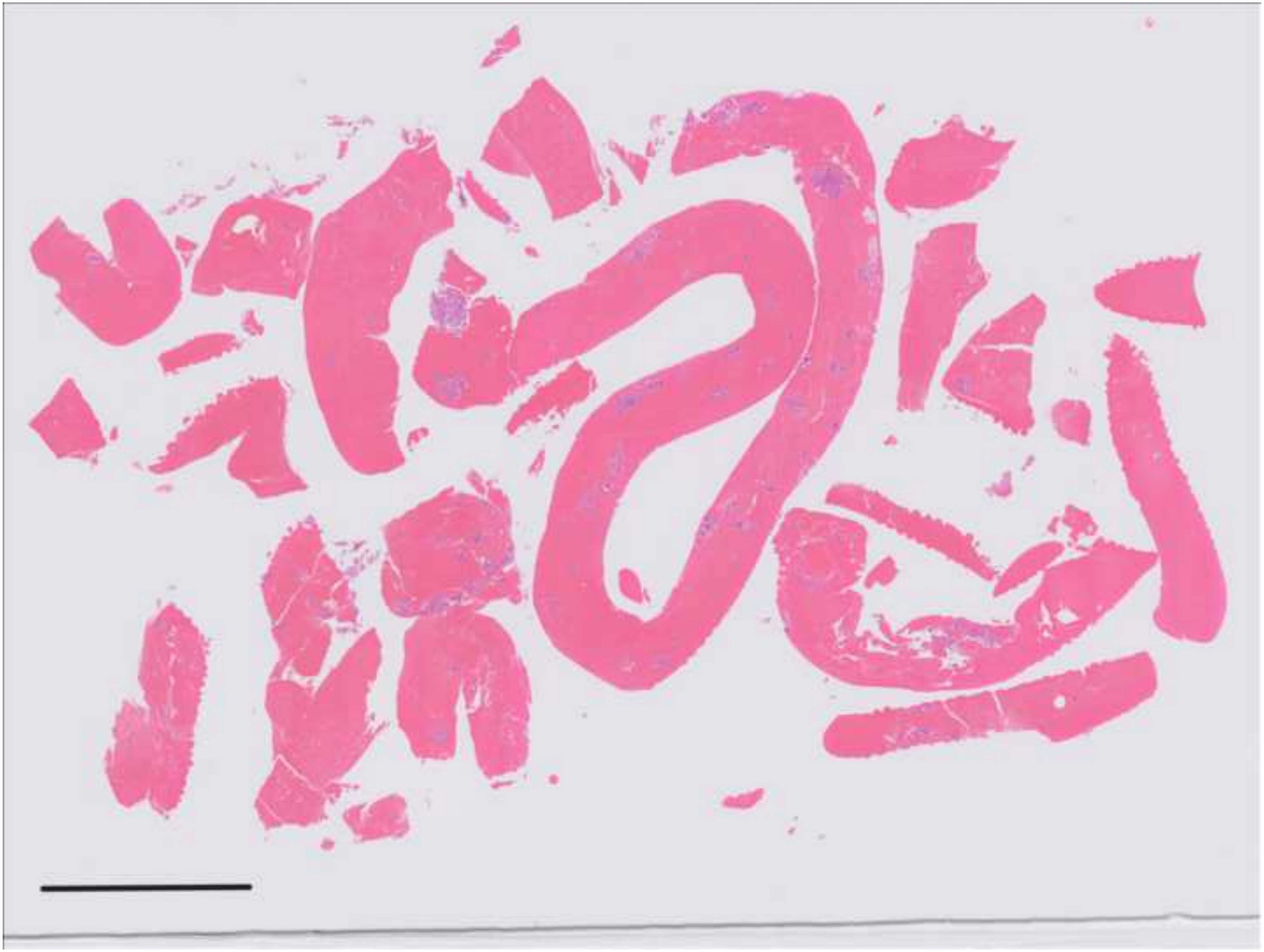

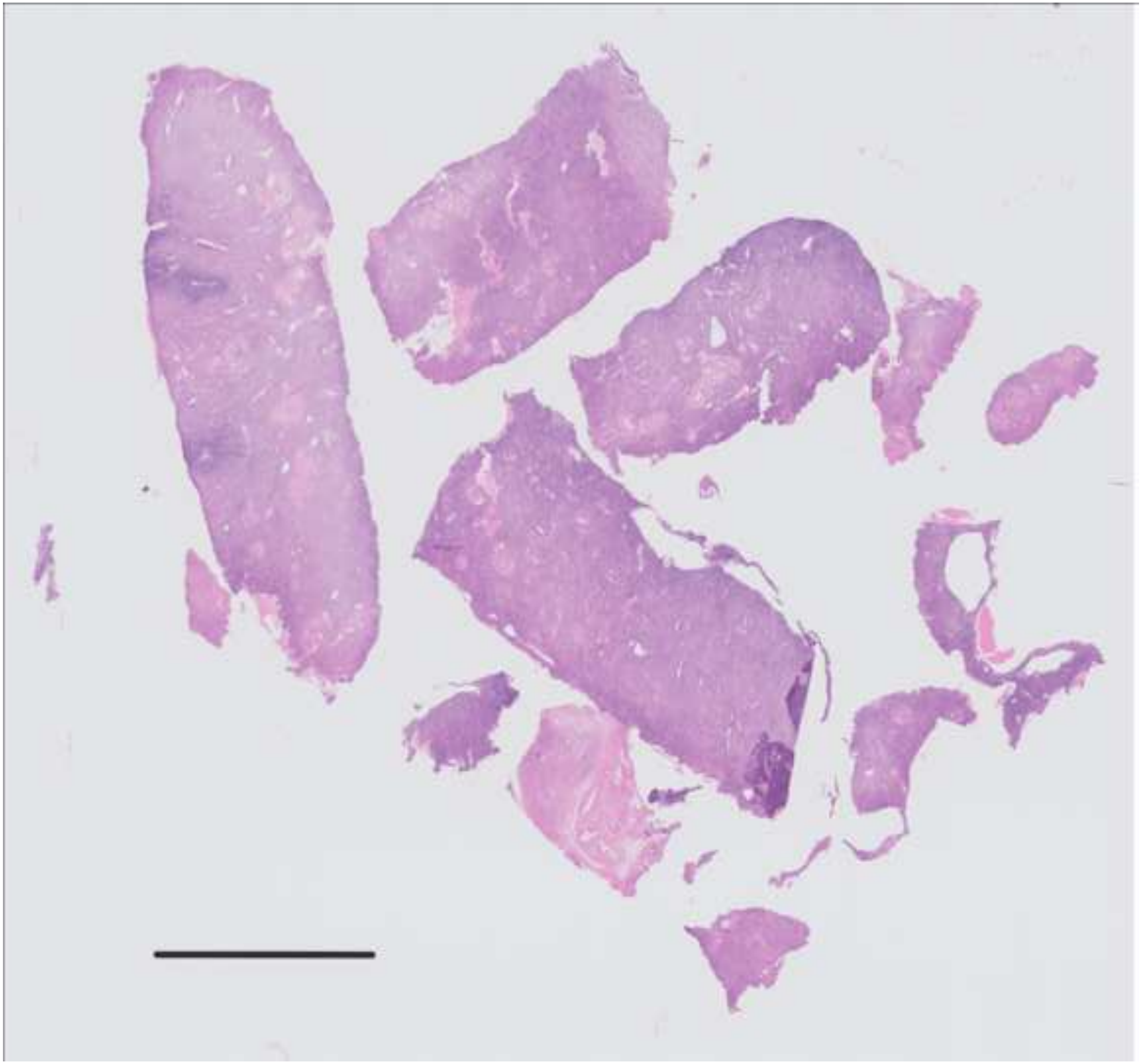

Click here to access/download;Figure;IC-EN-00351-01_thumbnail.png

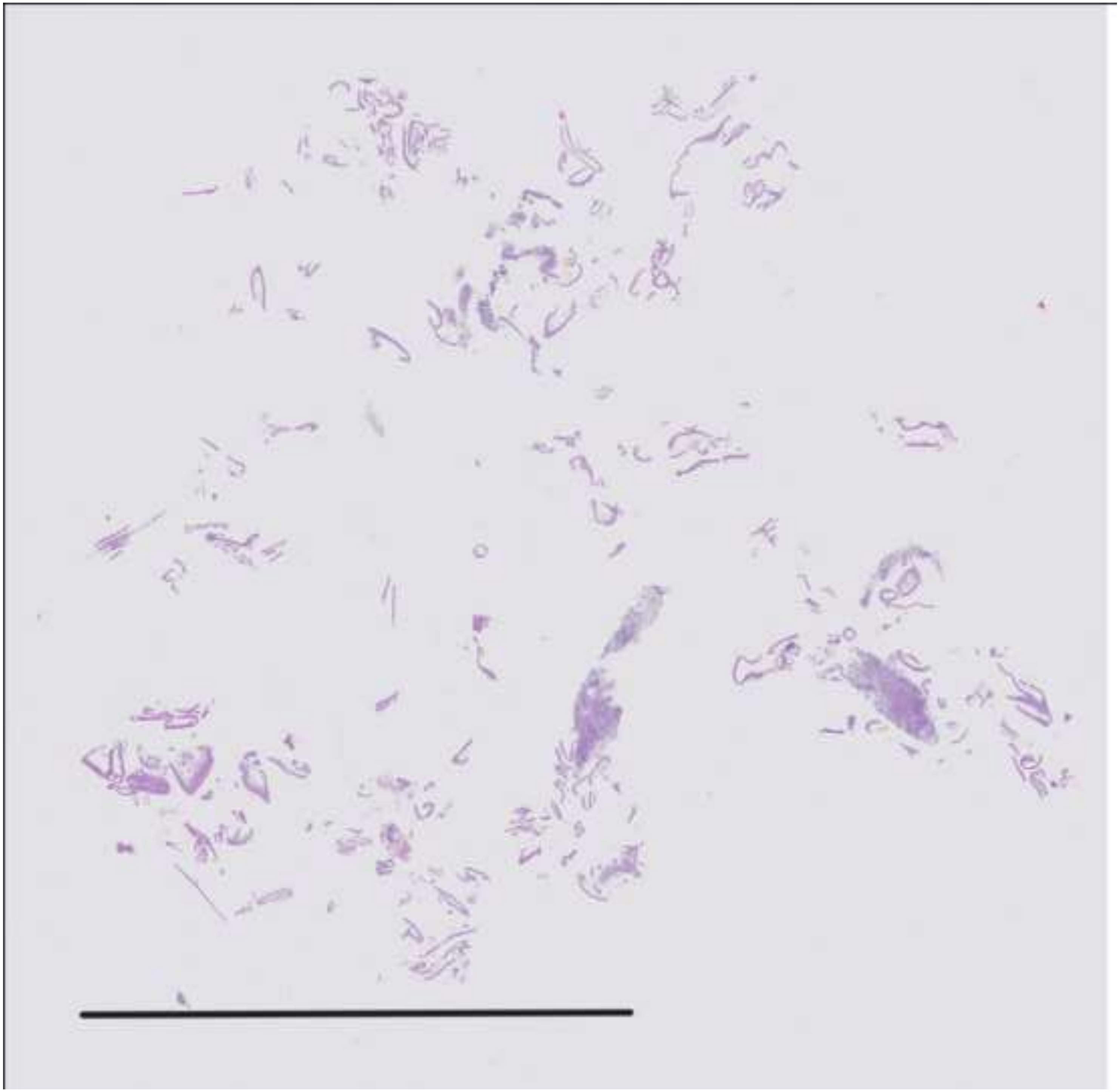

Click here to access/download;Figure;IC-EN-00889-01_thumbnail.png

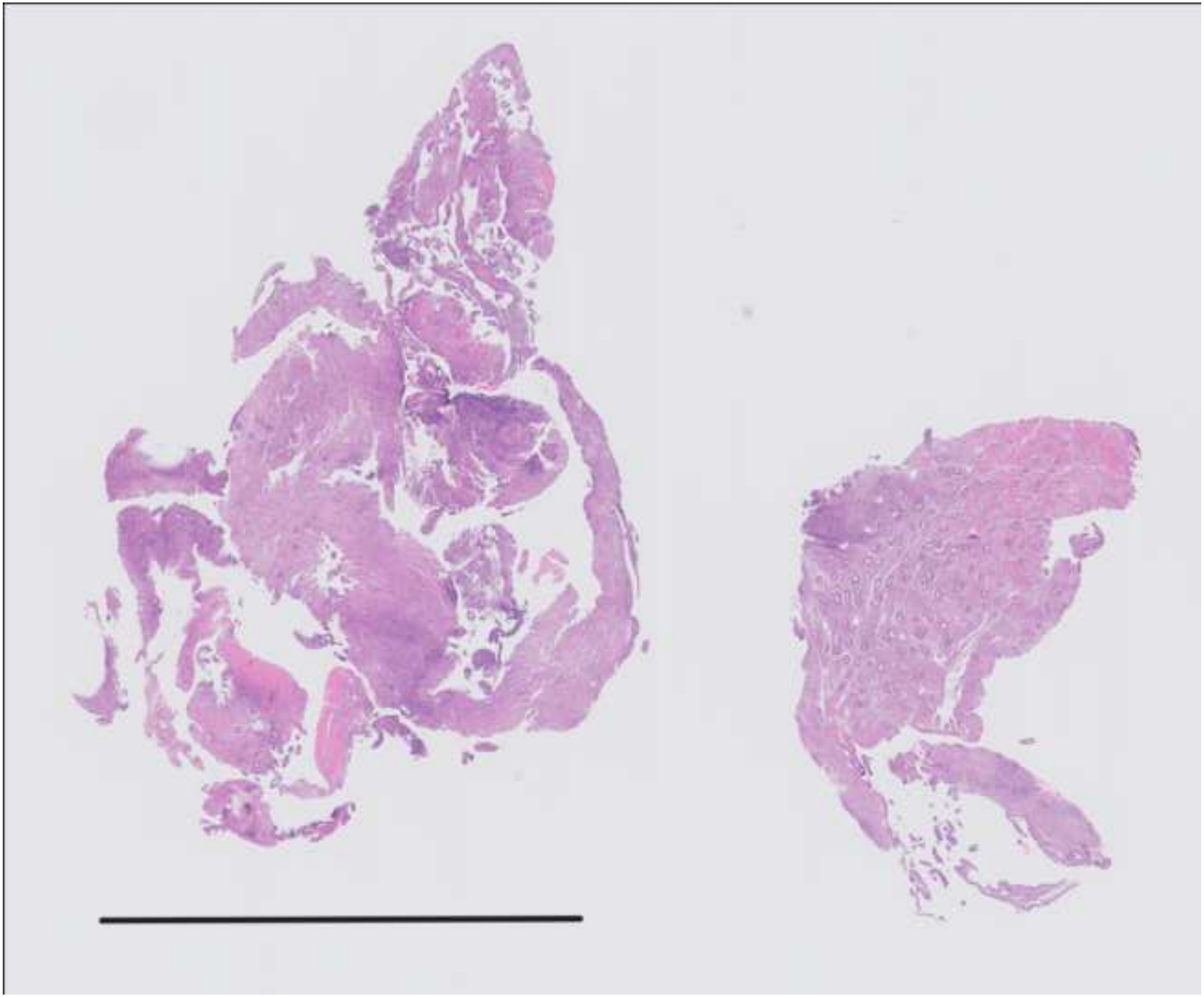

 Click here to access/download;Figure;New endo flow chart.jpg

1. Tissue Source & Variability

- Tissue sourced from 4 Glasgow hospitals: NG, SG, RAH, QEUH
- Independent processing: fixation, sectioning (3 or 4μm), H&E staining
- 8 lab-style combinations created to maximise WSI variability and reduce overfitting

↓

2. Slide Processing & Digitisation

- Slides scanned at QEUH using Philips Ultra Fast Scanner (UFS)
- WSIs stored in iSyntax format with Level 0 or 40x magnification (0.25μm/pixel)

↓

3. Annotation and Label Validation

- Initial case labels checked during annotation
- Corrected labels used for final dataset split

↓

4. Slide Categorisation

- 3 Main Classes: Malignant, Other or Benign, Insufficient
- 5 subtypes each for malignant and benign

↓

5. Data Splitting Strategy

- Test set: All slides from 2 entire labs (excluded from training/validation)
    - Plus 10% of slides randomly sampled from other 6 labs
- Training & Validation Sets: 90% of slides from other 6 labs,
  2/3 for training, 1/3 for validation
- Stratified to balance main and subcategories

Personal Cover

Click here to access/download;Personal Cover;2nd comments-Giga-Endometrial-27Jun25.docx

Editor GigaScience

School of Medicine
University of St Andrews
North Haugh
St Andrews UK
KY16 9TF

27 Jun 2025

Dear Editor,

We sincerely thank you for the opportunity to revise and resubmit our manuscript titled, **“Endometrial Whole Slide Images Dataset for Detection of Malignancy in Endometrial Biopsies (ID: GIGA-D-24-00211)”.** We are grateful to the reviewers for their thoughtful and constructive feedback during the second round of review.

In response to the reviewer’s comments, we have carefully revised the manuscript and prepared a detailed, point-by-point response outlining the changes made.

We hope that our responses and the updated manuscript meet the expectations of the reviewers and editorial team. We are grateful for your time and consideration in reviewing our revised submission and look forward to your feedback.

Yours sincerely,

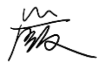

In Hwa Um

Reviewer #2:

1. ***Regarding my previous Comment 11, the authors have indeed provided a chart to present the data collection procedure and data split details. However, the chart is overly simplistic and should include more details. These details should include, but are not limited to, the inclusion and exclusion criteria for patients and the quality control standards for WSIs. Additionally, the data split details should clarify how the consistency of distribution across the training, validation, and test sets is maintained.***

We thank the reviewer for the helpful suggestion. In response, we have revised the manuscript to expand the description of the data collection and dataset splitting procedures and updated the associated chart (Figure 1) to reflect these details more comprehensively.

As this Data Note is derived from our previously published study in PLOS ONE (https://doi.org/10.1371/journal.pone.0282577), we provide further clarification regarding the dataset creation and structure. Cases were retrospectively identified by querying the pathology department archives using the topographic term "endometrium." Only slides that had been formally reported by a diagnostic pathologist were included. This ensured that the material met the quality threshold necessary for issuing a clinical diagnosis. Where required, additional histologic sections were prepared to ensure diagnostic adequacy. Subsequently, the study pathologists independently reviewed the candidate slides and confirmed that the diagnostic label could be reliably established based on material that was technically sufficient for clinical assessment.

To ensure the quality of the whole slide images (WSIs), a multi-stage quality control process was followed. Initially, slides underwent quality screening during routine diagnostic workflows. After scanning, each WSI was visually inspected to verify focus, resolution, staining adequacy, and absence of scanning artifacts. WSIs with poor image quality, insufficient tissue, incomplete metadata, or uncertain diagnosis were excluded. This dual-level review, involving both clinical and research pathologists, ensured that only high-quality and diagnostically reliable slides were included in the dataset. The slides were categorized into three diagnostic classes: "malignant," "other or benign," and "insufficient."

To ensure the model's ability to generalize across different laboratory protocols, the dataset was divided based on the originating laboratories' staining protocols. Specifically, the test set included all slides from two laboratories not represented in the training and validation sets, accounting for approximately 75% of the test data. Additionally, 10% of slides from the remaining six laboratories were randomly selected and added to the test set, making up the remaining 25%. The remaining 90% of slides from these six laboratories were split into training and validation sets, with two-thirds allocated for training and one-third for validation. This stratified

splitting ensured balanced representation across diagnostic categories and subcategories, facilitating robust model training and evaluation.

This structured approach to dataset division was designed to assess both in-distribution and out-of-distribution performance, providing insights into the model's generalisability across varying staining protocols and laboratory settings.

The updated chart (Figure 1) now reflects the complete case selection pathway, inclusion and exclusion criteria, multi-level quality control, and the principles applied in the dataset split. We believe that these revisions enhance the transparency and reproducibility of our dataset and address the reviewer's concerns regarding the simplicity of the initial figure.

2. ***Regarding my previous Comment 15, benchmark results for this dataset should be provided. The authors argue that these results were presented in a previous paper. I recommend that they summarize those results and present them appropriately in this manuscript. Additionally, the results in the previous paper pertain to the three-class classification of "malignant/other_benign/insufficient." I suggest that the authors also provide benchmark results for sub-category classification and WSI segmentation in this manuscript, as this would help highlight the utility of the dataset.***

In response to the reviewer's request for benchmark results, we have summarized the classification performance reported in our previously published study, which evaluated the dataset using three standard machine learning approaches: Random Forest, XGBoost, and a Convolutional Neural Network (CNN). The models were trained to perform a three-class classification task distinguishing "malignant," "other or benign," and "insufficient" whole slide images (WSIs).

Among these, the CNN achieved the highest accuracy in identifying malignant cases, with classification accuracy ranging from 89.8% to 92.1% depending on whether any tissue patches or majority-tissue patches were used. However, its overall accuracy (85.2%–90.8%) was slightly lower than that of the Random Forest model, which yielded the highest overall accuracy but underperformed in correctly identifying malignant cases. XGBoost provided a balanced performance, intermediate between the CNN and Random Forest classifiers. These results, presented in detail in Table 3 and Figure 9 of the original publication (PLOS ONE, https://doi.org/10.1371/journal.pone.0282577), offer a benchmark for future method development and validation.

We agree with the reviewer that presenting benchmark results beyond the three-class task would enhance the manuscript. While our initial work did not include experiments on sub-category classification or WSI segmentation, the dataset is structured to support both. Diagnostic subcategories are embedded within the dataset's metadata, allowing for fine-grained classification tasks, such as differentiating specific benign or malignant subtypes. Similarly, the high-resolution WSIs, combined with associated diagnostic labels and annotations, make the

dataset well-suited for training segmentation models aimed at identifying regions of diagnostic relevance.

We recognize the importance of these tasks for demonstrating the broader utility of the dataset and plan to pursue them in future work. Incorporating benchmark results for sub-category classification and WSI segmentation will help further establish this dataset as a robust resource for computational pathology research. This has been added in ‘Re-Use potential’ section. We appreciate the reviewer’s suggestion and have revised the manuscript to include this summary and contextual discussion.

3. ***Although the authors have revised the manuscript, they did not mark the changes, making it difficult for me to follow the specific revision. As a result, it is hard to determine whether the revised manuscript adequately addresses my concerns. I recommend that the authors highlight the changes in the manuscript.***

   We apologise for any inconvenience caused by the revision. Unfortunately, the Overleaf template does not allow us to highlight the changes directly.
