# Supplementary material for: Endometrial whole-slide images dataset for detection of malignancy in endometrial biopsies
Source: Gigascience. 2025 Dec 5;14:giaf147. doi: 10.1093/gigascience/giaf147 (PMC12751089; doi:10.1093/gigascience/giaf147)
Supplement: giaf147_Authors_Response_To_Reviewer_Comments_Original_Submission [file giaf147_authors_response_to_reviewer_comments_original_submission.pdf]

Author's Response To Reviewer Comments

Reviewer #1:

1. File format: The most common formats for WSIs are ".svs" and ".tif" and the most common python library used is "OpenSl
andrews.ac.uk%7C0510f47c971843087ded08dcb5449a05%7Cf85626cb0da849d3aa5864ef678ef01a%7C0%7C0%7C638584
In order to make sure the EC dataset reaches a vast audience, it is recommended to convert to one of the file formats suppor

Glencoe software was used to convert iSyntax format to ome-tiff, as described in the text. More information on this process c
In preliminary discussions with Dr. Armit before submission, we acknowledged that while the iSYNTAX format can pose certai
highlights the importance of maintaining accessibility to such datasets, as they contribute to the broader goal of improving in

2. Metadata: In order for the community to use this dataset, it would be important to include metadata such as age, survival

We do not have this information. Sex is obviously female, but in almost every case included the diagnosis would have no con
cases in our cohort are not cancerous, which is precisely why we believe this is a useful, unique addition to publicly available

3. Highlighting the utility of datasets: A missing piece in the manuscript is to showcase the real utility of the dataset. This cou
look like.

Preliminary results can be found in our cited primary research paper (Fell et al, "Detection of malignancy in whole slide image
The model categorises slides into, "malignant, "other or benign" and "insufficient" at the slide level. Additionally, the AI gener
We have not directly compared the performance differences between this AI model and nuclear morphological features. Howe
offer valuable insights since this is the only large study with histological images of endometrial biopsies. These resources can
Regarding model performance metrics, they are detailed in the publication. The study provides a baseline evaluation of the m

4. Data distribution: It would be good if authors provided the class label and data split for each of the eight different labs.
This table shows the data split for eight different laboratories.

StainingSiteID categopry train valid test
1 other_benign 157 67 26
1 malignant 73 31 10
1 insufficient 12 9 2
2 other_benign 146 74 26
2 malignant 72 30 12
2 insufficient 21 8 0
3 other_benign 144 75 25
3 malignant 61 32 10
3 insufficient 17 5 2
4 other_benign 138 78 23
4 malignant 74 37 12
4 insufficient 14 8 3
5 other_benign 136 70 23
5 malignant 63 33 10
5 insufficient 13 7 2
6 other_benign 0 0 222
6 malignant 0 0 107
6 insufficient 0 0 21
7 other_benign 133 67 22
7 malignant 64 29 11
7 insufficient 16 8 2
8 other_benign 0 0 236
8 malignant 0 0 100
8 insufficient 0 0 18

5. In case authors don't already plan to do it, it would be good if the trained blood and mucus detection model is also made a

The complete process is comprehensively described in Fell et al. which we have cited, “Detection of malignancy in whole slide
(https://zenodo.org/record/7674764), and this is cited in reference 10.

6. Table 1 currently includes the distribution of samples. Since one patient can possibly have more than one sample, it would

There is only one sample per patient.

7. Under "annotation process", the authors mention a mix of experts were used for annotations. How many annotators were

Four histopathologically trained biomedical scientists and four histopathologists annotated biopsies. All histopathologists who
experience, with some having over 15 years of expertise in gynaecological pathology. To ensure high-quality and standardise

8. Abstract: The abstract could highlight the class labels and their importance

Abstract has been improved as requested.

9. It would be good to add "scale" example for 1mm for all images in figures 1-4.
It has been done as requested.

Reviewer #2:

10. A detailed summary of existing databases related to endometrial cancer should be provided, including but not limited to t
Our primary research paper (Fell et al, “Detection of malignancy in whole slide images of endometrial cancer biopsies using a
cases, whereas our dataset has 2909 cases. There is no comparable dataset covering a wide range of endometrial pathology
11. It would be beneficial to present the data collection procedure and data split details using a flow chart or a table. In the c

Figure has been added as requested.

12. More details about this database should be provided. For instance, include information on the distribution of the proportio
The tissue on the slide has been fully annotated by expert pathologists. Patients were obviously female and predominantly Ca
13. In Table 1, please clarify what the subcategory “Other” refers to. Additionally, explain the potential uses for slides labelle
One problem in diagnostic pathology is how to deal with small samples that may not be representative or provide conclusive
the patient. Being more confident about defining an inadequate sample will help workflow, ensure safety and minimise unnec
14. It would be better to explain what deep learning tasks this database can be utilized for, such as patch classification, whol
The database can be used for whole slide image classification or patch classification. Some slides contain a mix of classes and
Section 2.3 of our primary research paper (Fell et al, “Detection of malignancy in whole slide images of endometrial cancer bi
15. To validate the proposed database, benchmark results should be provided. For example, patch classification results using
This was described in the original research paper (Fell et al, “Detection of malignancy in whole slide images of endometrial ca

16. It would be better to show typical regions for each subcategory and briefly summarize their differences.

Figure has been added as requested.

17. In Table 1, the largest number of these subcategory has 518 slides, while the smallest number is only 8. The authors sho
The cases were obtained from a biorepository and reflect the mix of conditions received by the laboratories. Rare things are r
common conditions with confidence rather than necessarily focussing on diagnosing rare conditions.

Cookie Preference Center

We use cookies which are necessary to make our site work. We may also use additional cookies to analyze, improve and personalize our content and your digital experience. For more information, see our Cookie Policy.

You may choose not to allow some types of cookies. However, blocking some types may impact your experience of our site and the services we are able to offer. See the different category headings below to find out more or change your settings.

Allow all

Manage Consent Preferences

Strictly Necessary Cookies

Always active

These cookies are necessary for the website to function and cannot be switched off in our systems. They are usually only set in response to actions made by you which amount to a request for services, such as setting your privacy preferences, logging in or filling in forms. You can set your browser to block or alert you about these cookies, but some parts of the site will not then work.

Functional Cookies

These cookies enable the website to provide enhanced functionality and personalisation. They may be set by us or by third party providers whose services we have added to our pages. If you do not allow these cookies then some or all of these services may not function properly.

Performance Cookies

These cookies allow us to count visits and traffic sources so we can measure and improve the performance of our site. They help us to know which pages are the most and least popular and see how visitors move around the site.

Targeting Cookies

These cookies may be set through our site by our advertising partners. They may be used by those companies to build a profile of your interests and show you relevant adverts on other sites. If you do not allow these cookies, you will experience less targeted advertising.

Cookie List

Clear

Apply Cancel
Consent Leg.Interest

Confirm my choices

Powered by onetrust
