# Supplementary material for: Endometrial whole-slide images dataset for detection of malignancy in endometrial biopsies
Source: Gigascience. 2025 Dec 5;14:giaf147. doi: 10.1093/gigascience/giaf147 (PMC12751089; doi:10.1093/gigascience/giaf147)
Supplement: giaf147_Authors_Response_To_Reviewer_Comments_Revision_1 [file giaf147_authors_response_to_reviewer_comments_revision_1.pdf]

Author's Response To Reviewer Comments

Reviewer #2:
1. Regarding my previous Comment 11, the authors have indeed provided a chart to present the data collection procedure and data split details. However, the chart is overly simplistic and should include more details. These details should include, but are not limited to, the inclusion and exclusion criteria for patients and the quality control standards for WSIs. Additionally, the data split details should clarify how the consistency of distribution across the training, validation, and test sets is maintained.

We thank the reviewer for the helpful suggestion. In response, we have revised the manuscript to expand the description of the data collection and dataset splitting procedures and updated the associated chart (Figure 1) to reflect these details more comprehensively.
As this Data Note is derived from our previously published study in PLOS ONE (https://doi.org/10.1371/journal.pone.0282577), we provide further clarification regarding the dataset creation and structure. Cases were retrospectively identified by querying the pathology department archives using the topographic term "endometrium." Only slides that had been formally reported by a diagnostic pathologist were included. This ensured that the material met the quality threshold necessary for issuing a clinical diagnosis. Where required, additional histologic sections were prepared to ensure diagnostic adequacy. Subsequently, the study pathologists independently reviewed the candidate slides and confirmed that the diagnostic label could be reliably established based on material that was technically sufficient for clinical assessment.
To ensure the quality of the whole slide images (WSIs), a multi-stage quality control process was followed. Initially, slides underwent quality screening during routine diagnostic workflows. After scanning, each WSI was visually inspected to verify focus, resolution, staining adequacy, and absence of scanning artifacts. WSIs with poor image quality, insufficient tissue, incomplete metadata, or uncertain diagnosis were excluded. This dual-level review, involving both clinical and research pathologists, ensured that only high-quality and diagnostically reliable slides were included in the dataset. The slides were categorized into three diagnostic classes: "malignant," "other or benign," and "insufficient."
To ensure the model's ability to generalize across different laboratory protocols, the dataset was divided based on the originating laboratories' staining protocols. Specifically, the test set included all slides from two laboratories not represented in the training and validation sets, accounting for approximately 75% of the test data. Additionally, 10% of slides from the remaining six laboratories were randomly selected and added to the test set, making up the remaining 25%. The remaining 90% of slides from these six laboratories were split into training and validation sets, with two-thirds allocated for training and one-third for validation. This stratified splitting ensured balanced representation across diagnostic categories and subcategories, facilitating robust model training and evaluation.
This structured approach to dataset division was designed to assess both in-distribution and out-of-distribution performance, providing insights into the model's generalisability across varying staining protocols and laboratory settings.
The updated chart (Figure 1) now reflects the complete case selection pathway, inclusion and exclusion criteria, multi-level quality control, and the principles applied in the dataset split. We believe that these revisions enhance the transparency and reproducibility of our dataset and address the reviewer's concerns regarding the simplicity of the initial figure.
2. Regarding my previous Comment 15, benchmark results for this dataset should be provided. The authors argue that these results were presented in a previous paper. I recommend that they summarize those results and present them appropriately in this manuscript. Additionally, the results in the previous paper pertain to the three-class classification of "malignant/other_benign/insufficient." I suggest that the authors also provide benchmark results for sub-category classification and WSI segmentation in this manuscript, as this would help highlight the utility of the dataset.

In response to the reviewer's request for benchmark results, we have summarized the classification performance reported in our previously published study, which evaluated the dataset using three standard machine learning approaches: Random Forest, XGBoost, and a Convolutional Neural Network (CNN). The models were trained to perform a three-class classification task distinguishing "malignant," "other or

benign," and "insufficient" whole slide images (WSIs).
Among these, the CNN achieved the highest accuracy in identifying malignant cases, with classification accuracy ranging from 89.8% to 92.1% depending on whether any tissue patches or majority-tissue patches were used. However, its overall accuracy (85.2%–90.8%) was slightly lower than that of the Random Forest model, which yielded the highest overall accuracy but underperformed in correctly identifying malignant cases. XGBoost provided a balanced performance, intermediate between the CNN and Random Forest classifiers. These results, presented in detail in Table 3 and Figure 9 of the original publication (PLOS ONE, https://doi.org/10.1371/journal.pone.0282577), offer a benchmark for future method development and validation.
We agree with the reviewer that presenting benchmark results beyond the three-class task would enhance the manuscript. While our initial work did not include experiments on sub-category classification or WSI segmentation, the dataset is structured to support both. Diagnostic subcategories are embedded within the dataset's metadata, allowing for fine-grained classification tasks, such as differentiating specific benign or malignant subtypes. Similarly, the high-resolution WSIs, combined with associated diagnostic labels and annotations, make the dataset well-suited for training segmentation models aimed at identifying regions of diagnostic relevance.
We recognize the importance of these tasks for demonstrating the broader utility of the dataset and plan to pursue them in future work. Incorporating benchmark results for sub-category classification and WSI segmentation will help further establish this dataset as a robust resource for computational pathology research. This has been added in 'Re-Use potential' section. We appreciate the reviewer's suggestion and have revised the manuscript to include this summary and contextual discussion.

3. Although the authors have revised the manuscript, they did not mark the changes, making it difficult for me to follow the specific revision. As a result, it is hard to determine whether the revised manuscript adequately addresses my concerns. I recommend that the authors highlight the changes in the manuscript.

We apologise for any inconvenience caused by the revision. Unfortunately, the Overleaf template does not allow us to highlight the changes directly.

Editorial Manager®

Cookie Preference Center

We use cookies which are necessary to make our site work. We may also use additional cookies to analyze, improve and personalize our content and your digital experience. For more information, see our Cookie Policy.

You may choose not to allow some types of cookies. However, blocking some types may impact your experience of our site and the services we are able to offer. See the different category headings below to find out more or change your settings.

Allow all

Manage Consent Preferences

Strictly Necessary Cookies

Always active

These cookies are necessary for the website to function and cannot be switched off in our systems. They are usually only set in response to actions made by you which amount to a request for services, such as setting your privacy preferences, logging in or filling in forms. You can set your browser to block or alert you about these cookies, but some parts of the site will not then work.

Functional Cookies

These cookies enable the website to provide enhanced functionality and personalisation. They may be set by us or by third party providers whose services we have added to our pages. If you do not allow these cookies then some or all of these services may not function properly.

Performance Cookies

These cookies allow us to count visits and traffic sources so we can measure and improve the performance of our site. They help us to know which pages are the most and least popular and see how visitors move around the site.

Targeting Cookies

These cookies may be set through our site by our advertising partners. They may be used by those companies to build a profile of your interests and show you relevant adverts on other sites. If you do not allow these cookies, you will experience less targeted advertising.

Cookie List

Clear

Apply Cancel

Consent Leg.Interest

Confirm my choices

Powered by onetrust
