# Supplementary material for: Endometrial whole-slide images dataset for detection of malignancy in endometrial biopsies
Source: Gigascience. 2025 Dec 5;14:giaf147. doi: 10.1093/gigascience/giaf147 (PMC12751089; doi:10.1093/gigascience/giaf147)
Supplement: giaf147_Reviewer_1_Report_Original_Submission — Sonali Andani -- 7/8/2024 [file giaf147_reviewer_1_report_original_submission.pdf]

Reviewer Report

Title: Endometrial Whole Slide Images Dataset for Detection of Malignancy in Endometrial Biopsies

Version: Original Submission **Date:** 7/8/2024

Reviewer name: Sonali Andani

Reviewer Comments to Author:

The paper reads well and the data seems promising. The authors could improve the work based on following suggestions:1. File format: The most common formats for WSIs are ".svs" and ".tif" and the most common python library used is "OpenSlide". Openslide does not support "isyntax" file format (reference: https://openslide.org/api/python/). In order to make sure the EC dataset reaches a vast audience, it is recommended to convert to one of the file formats supported by OpenSlide library.2. Metadata: In order for the community to use this dataset, it would be important to include metadata such as age, survival data, stage, response to treatment, if any treatment given, molecular class or any available metadata. For reference, the authors could use the metadata available for TCGA datasets as a reference.3. Highlighting the utility of datasets: A missing piece in the manuscript is to showcase the real utility of the dataset. This could include comparison with other publicly available EC datasets. What does the model performance look like in terms of metrics such as F1 score, precision, recall. If the nuclei features are included, what would the performance look like.4. Data distribution: It would be good if authors provided the class label and data split for each of the eight different labs.5. In case authors don't already plan to do it, it would be good if the trained blood and mucus detection model is also made available.Minor changes:1. Table 1 currently includes the distribution of samples. Since one patient can possibly have more than one sample, it would be good to include distribution of patients as well.2. Under "annotation process", the authors mention a mix of experts were used for annotations. How many annotators were exactly used for the task?3. Abstract: The abstract could highlight the class labels and their importance.4. It would be good to add "scale" example for 1mm for all images in figures 1-4.

Level of Interest

Please indicate how interesting you found the manuscript: Choose an item.

Quality of Written English

Please indicate the quality of language in the manuscript: Choose an item.

Declaration of Competing Interests

Please complete a declaration of competing interests, considering the following questions:

- Have you in the past five years received reimbursements, fees, funding, or salary from an organisation that may in any way gain or lose financially from the publication of this manuscript, either now or in the future?
- Do you hold any stocks or shares in an organisation that may in any way gain or lose financially from the publication of this manuscript, either now or in the future?
- Do you hold or are you currently applying for any patents relating to the content of the manuscript?
- Have you received reimbursements, fees, funding, or salary from an organization that holds or has applied for patents relating to the content of the manuscript?
- Do you have any other financial competing interests?
- Do you have any non-financial competing interests in relation to this paper?

If you can answer no to all of the above, write 'I declare that I have no competing interests' below. If your reply is yes to any, please give details below.

I declare that I have no competing interests

I agree to the open peer review policy of the journal. I understand that my name will be included on my report to the authors and, if the manuscript is accepted for publication, my named report including any attachments I upload will be posted on the website along with the authors' responses. I agree for my report to be made available under an Open Access Creative Commons CC-BY license (http://creativecommons.org/licenses/by/4.0/). I understand that any comments which I do not wish to be included in my named report can be included as confidential comments to the editors, which will not be published.

Choose an item.

To further support our reviewers, we have joined with Publons, where you can gain additional credit to further highlight your hard work (see: https://publons.com/journal/530/gigascience). On publication of this paper, your review will be automatically added to Publons, you can then choose whether or not to claim your Publons credit. I understand this statement.

Yes Choose an item.
