# Supplementary material for: Endometrial whole-slide images dataset for detection of malignancy in endometrial biopsies
Source: Gigascience. 2025 Dec 5;14:giaf147. doi: 10.1093/gigascience/giaf147 (PMC12751089; doi:10.1093/gigascience/giaf147)
Supplement: giaf147_Reviewer_2_Report_Original_Submission — Liansheng Wang -- 8/3/2024 [file giaf147_reviewer_2_report_original_submission.pdf]

Reviewer Report

Title: Endometrial Whole Slide Images Dataset for Detection of Malignancy in Endometrial Biopsies

Version: Original Submission **Date:** 8/3/2024

Reviewer name: Liansheng Wang

Reviewer Comments to Author:

Summary:This paper introduces a new database of endometrial biopsies, comprising 2909 whole slide images. Both slide-level labels and fine-grained annotations are meticulously provided. To some extent, this database has the potential to contribute to the advancement of AI-based diagnostic research and applications for endometrial cancer. However, this manuscript still contains several issues that need to be addressed.Major:1.A detailed summary of existing databases related to endometrial cancer should be provided, including but not limited to the TCGA UCEC. Specifically, it is important to highlight the advantages or contributions of the database mentioned in this paper compared to the existing datasets.2.It would be beneficial to present the data collection procedure and data split details using a flow chart or a table. In the current manuscript, these two sections are not easy to understand.3.More details about this database should be provided. For instance, include information on the distribution of the proportion of annotated areas in the entire slide, as well as the racial, gender, and age distribution of the whole slide images.4.In Table 1, please clarify what the subcategory "Other" refers to. Additionally, explain the potential uses for slides labeled as "insufficient".5.It would be better to explain what deep learning tasks this database can be utilized for, such as patch classification, whole slide image classification and segmentation. Additionally, briefly describe how to process the annotations for each corresponding task.6.To validate the proposed database, benchmark results should be provided. For example, patch classification results using CNN models and WSI classification results using a multiple instance learning model.Minor:1.It would be better to show typical regions for each subcategory and briefly summarize their differences.2.In Table 1, the largest number of these subcategory has 518 slides, while the smallest number is only 8. The authors should explain why the number of WSIs in each category is unbalanced.

Level of Interest

Please indicate how interesting you found the manuscript: Choose an item.

Quality of Written English

Please indicate the quality of language in the manuscript: Choose an item.

Declaration of Competing Interests

Please complete a declaration of competing interests, considering the following questions:

- Have you in the past five years received reimbursements, fees, funding, or salary from an organisation that may in any way gain or lose financially from the publication of this manuscript, either now or in the future?
- Do you hold any stocks or shares in an organisation that may in any way gain or lose financially from the publication of this manuscript, either now or in the future?
- Do you hold or are you currently applying for any patents relating to the content of the manuscript?
- Have you received reimbursements, fees, funding, or salary from an organization that holds or has applied for patents relating to the content of the manuscript?
- Do you have any other financial competing interests?
- Do you have any non-financial competing interests in relation to this paper?

If you can answer no to all of the above, write 'I declare that I have no competing interests' below. If your reply is yes to any, please give details below.

I declare that I have no competing interests

I agree to the open peer review policy of the journal. I understand that my name will be included on my report to the authors and, if the manuscript is accepted for publication, my named report including any attachments I upload will be posted on the website along with the authors' responses. I agree for my report to be made available under an Open Access Creative Commons CC-BY license (http://creativecommons.org/licenses/by/4.0/). I understand that any comments which I do not wish to be included in my named report can be included as confidential comments to the editors, which will not be published.

Choose an item.

To further support our reviewers, we have joined with Publons, where you can gain additional credit to further highlight your hard work (see: https://publons.com/journal/530/gigascience). On publication of this paper, your review will be automatically added to Publons, you can then choose whether or not to claim your Publons credit. I understand this statement.

Yes Choose an item.
