# Supplementary material for: Endometrial whole-slide images dataset for detection of malignancy in endometrial biopsies
Source: Gigascience. 2025 Dec 5;14:giaf147. doi: 10.1093/gigascience/giaf147 (PMC12751089; doi:10.1093/gigascience/giaf147)
Supplement: giaf147_Reviewer_2_Report_Revision_1 — Liansheng Wang -- 4/4/2025 [file giaf147_reviewer_2_report_revision_1.pdf]

Reviewer Report

Title: Endometrial Whole Slide Images Dataset for Detection of Malignancy in Endometrial Biopsies

Version: Revision 1 **Date:** 4/4/2025

Reviewer name: Liansheng Wang

Reviewer Comments to Author:

The point-by-point responses have addressed most of my concerns. However, I believe the following issues still need to be resolved before the paper is ready for publication.(1) Regarding my previous Comment 11, the authors have indeed provided a chart to present the data collection procedure and data split details. However, the chart is overly simplistic and should include more details. These details should include, but are not limited to, the inclusion and exclusion criteria for patients and the quality control standards for WSIs. Additionally, the data split details should clarify how the consistency of distribution across the training, validation, and test sets is maintained.(2) Regarding my previous Comment 15, benchmark results for this dataset should be provided. The authors argue that these results were presented in a previous paper. I recommend that they summarize those results and present them appropriately in this manuscript. Additionally, the results in the previous paper pertain to the three-class classification of "malignant/other_benign/insufficient." I suggest that the authors also provide benchmark results for sub-category classification and WSI segmentation in this manuscript, as this would help highlight the utility of the dataset.(3) Although the authors have revised the manuscript, they did not mark the changes, making it difficult for me to follow the specific revision. As a result, it is hard to determine whether the revised manuscript adequately addresses my concerns. I recommend that the authors highlight the changes in the manuscript.

Level of Interest

Please indicate how interesting you found the manuscript: Choose an item.

Quality of Written English

Please indicate the quality of language in the manuscript: Choose an item.

Declaration of Competing Interests

Please complete a declaration of competing interests, considering the following questions:

- Have you in the past five years received reimbursements, fees, funding, or salary from an organisation that may in any way gain or lose financially from the publication of this manuscript, either now or in the future?
- Do you hold any stocks or shares in an organisation that may in any way gain or lose financially from the publication of this manuscript, either now or in the future?

- Do you hold or are you currently applying for any patents relating to the content of the manuscript?
- Have you received reimbursements, fees, funding, or salary from an organization that holds or has applied for patents relating to the content of the manuscript?
- Do you have any other financial competing interests?
- Do you have any non-financial competing interests in relation to this paper?

If you can answer no to all of the above, write 'I declare that I have no competing interests' below. If your reply is yes to any, please give details below.

I declare that I have no competing interests

I agree to the open peer review policy of the journal. I understand that my name will be included on my report to the authors and, if the manuscript is accepted for publication, my named report including any attachments I upload will be posted on the website along with the authors' responses. I agree for my report to be made available under an Open Access Creative Commons CC-BY license (http://creativecommons.org/licenses/by/4.0/). I understand that any comments which I do not wish to be included in my named report can be included as confidential comments to the editors, which will not be published.

Choose an item.

To further support our reviewers, we have joined with Publons, where you can gain additional credit to further highlight your hard work (see: https://publons.com/journal/530/gigascience). On publication of this paper, your review will be automatically added to Publons, you can then choose whether or not to claim your Publons credit. I understand this statement.

Yes Choose an item.
